# AtEAF1 is a potential platform protein for Arabidopsis NuA4 acetyltransferase complex

**DOI:** 10.1186/s12870-015-0461-1

**Published:** 2015-03-05

**Authors:** Tomasz Bieluszewski, Lukasz Galganski, Weronika Sura, Anna Bieluszewska, Mateusz Abram, Agnieszka Ludwikow, Piotr Andrzej Ziolkowski, Jan Sadowski

**Affiliations:** Department of Biotechnology, Institute of Molecular Biology and Biotechnology, Adam Mickiewicz University, Umultowska 89, 61-614 Poznań, Poland; Department of Molecular Virology, Institute of Experimental Biology, Adam Mickiewicz University, Umultowska 89, 61-614 Poznań, Poland; Department of Plant Sciences, University of Cambridge, Downing Street, CB2 3EA, Cambridge, UK

**Keywords:** NuA4, EAF1, YAF9, *Arabidopsis thaliana*, histone acetylation, PIE1

## Abstract

**Background:**

Histone acetyltransferase complex NuA4 and histone variant exchanging complex SWR1 are two chromatin modifying complexes which act cooperatively in yeast and share some intriguing structural similarities. Protein subunits of NuA4 and SWR1-C are highly conserved across eukaryotes, but form different multiprotein arrangements. For example, the human TIP60-p400 complex consists of homologues of both yeast NuA4 and SWR1-C subunits, combining subunits necessary for histone acetylation and histone variant exchange. It is currently not known what protein complexes are formed by the plant homologues of NuA4 and SWR1-C subunits.

**Results:**

We report on the identification and molecular characterization of AtEAF1, a new subunit of Arabidopsis NuA4 complex which shows many similarities to the platform protein of the yeast NuA4 complex. AtEAF1 copurifies with Arabidopsis homologues of NuA4 and SWR1-C subunits ARP4 and SWC4 and interacts physically with AtYAF9A and AtYAF9B, homologues of the YAF9 subunit. Plants carrying a T-DNA insertion in one of the genes encoding AtEAF1 showed decreased *FLC* expression and early flowering, similarly to *Atyaf9* mutants. Chromatin immunoprecipitation analyses of the single mutant *Ateaf1b*-*2* and artificial miRNA knock-down *Ateaf1* lines showed decreased levels of H4K5 acetylation in the promoter regions of major flowering regulator genes, further supporting the role of AtEAF1 as a subunit of the plant NuA4 complex.

**Conclusions:**

Growing evidence suggests that the molecular functions of the NuA4 and SWR1 complexes are conserved in plants and contribute significantly to plant development and physiology. Our work provides evidence for the existence of a yeast-like EAF1 platform protein in *A. thaliana*, filling an important gap in the knowledge about the subunit organization of the plant NuA4 complex.

**Electronic supplementary material:**

The online version of this article (doi:10.1186/s12870-015-0461-1) contains supplementary material, which is available to authorized users.

## Background

Eukaryotic chromatin has evolved for seemingly contradictory functions. It ensures compaction and protection of genetic material, but also controls diverse processes including transcription, replication and DNA repair that require a relatively open and dynamic chromatin structure. This mixture of robustness and flexibility is achieved by a number of specialized enzymes that remodel chromatin or modify nucleosomes by covalent histone modifications. Different chromatin modifications can have a combinatorial effect on chromatin properties and influence each other by guiding, stimulating or inhibiting chromatin modifying enzymes.

One of the best studied examples of an interplay between different types of chromatin modifications is the strong functional relationship between histone acetylation and chromatin remodeling. Protein domains specialized in specific recognition of acetylated histone tails are often found in chromatin remodeling complexes. Acetylation of nucleosomal histones has a strong influence on the action of chromatin remodeling complexes such as RSC [1], SWI/SNF [2], INO80 [3] or SWR1-C [4].

In the case of SWR1-C, this link seems to go much further. In yeast, the histone variant exchange reaction, catalyzed by SWR1-C is stimulated by the NuA4 complex through acetylation of nucleosomal histones [4]. These two protein complexes not only cooperate, but also share interesting structural similarities. Each of the two complexes is formed by more than ten different protein subunits organized into modules [5]. One of the modules, composed of proteins ARP4, SWC4, YAF9 and monomeric Actin, is common to both complexes [6]. Furthermore, a crucial role in the integrity of NuA4 and SWR1-C is played by proteins EAF1 and SWR1, respectively, which organize different modules into a functional complex. Although EAF1 lacks the ATPase domain, central to the chromatin remodeling activity of SWR1-C, it shares with SWR1 the HSA domain which is necessary for binding of the common module ARP4-SWC4-YAF9-Actin [5,7].

In human, homologues of yeast NuA4 and SWR1-C subunits form a hybrid complex called TIP60-p400. How far the functions of the fused complexes are conserved is yet to be determined. Whereas the ATPase activity of p400 enables H2A.Z deposition through histone exchange [8], the HAT activity of the TIP60 subunit seems to be blocked by the association with p400 [9]. Nevertheless, subunit conservation between SWR1, NuA4 and TIP60-p400 implies a strong evolutionary link. One attractive explanation of the relationship between SWR1-C, NuA4 and TIP60-p400 points to the fact that the integrity of the three complexes depends on their platform subunits SWR1, EAF1 and p400, respectively. The complex is formed when the remaining subunits bind, directly or as a part of a protein module, to several conserved features of the platform protein. Strikingly, p400 combines the features of SWR1 and EAF1. A synthetic p400-like construct, obtained by insertion of the ATPase domain of SWR1 between the HSA (helicase/SANT–associated) and SANT (Swi3, Ada2, N-Cor, and TFIIIB) domains of EAF1, reconstituted a TIP60-p400-like complex when expressed in yeast [5]. The authors of the study concluded that a similar rearrangement could have given rise to the p400-like architecture in higher eukaryotes [5].

Outside Metazoa, the best characterized example of a domain architecture similar to that of p400 is PIE1, a protein necessary for incorporation of H2A.Z into nucleosomes in *Arabidopsis thaliana* [10,11]. HSA, ATPase and SANT domains are all present in PIE1 and show a high degree of sequence similarity to the corresponding domains of p400. Although most of the subunits of NuA4 and SWR1-C have clear homologues in Arabidopsis, no study has addressed the question of whether PIE1 can organize these proteins into a hybrid complex similar to TIP60-p400. Importantly, a plant homologue of EAF1 has not been identified so far, which calls into question the existence of an independent NuA4 complex in plants.

Up to now, studies in plants have embraced homologues of only three subunits of the yeast NuA4 complex, ESA1, YAF9 and EAF3. ESA1, the only essential histone acetyltransferase in *S. cerevisiae* and the catalytic subunit of the NuA4 complex [12], has two homologues in *A. thaliana*, HAM1 (Histone Acetyltransferase of the MYST family 1) and HAM2. Both proteins specifically acetylate lysine 5 of the histone H4 [13] and are functionally redundant, as the single mutants display no developmental phenotype, while a double mutant is nonviable [14]. Reduction of HAM1 and HAM2 transcript levels results in decreased expression of the negative flowering regulator *FLOWERING LOCUS C* (*FLC*) and premature transition to flowering. This change is accompanied by a decrease in H4 acetylation in the chromatin of *FLC* [15]. Similar effects were observed in plants deficient in AtYAF9A, one of the two Arabidopsis homologues of yeast YAF9, a subunit shared by NuA4 and SWR1-C [16]. Interestingly, a recent study shows that simultaneous loss of function of two *A. thaliana* homologues of EAF3 results in late flowering, also mediated by reduced histone acetylation which, in this case, disrupts the expression of flowering inducer *FLOWERING LOCUS T (FT)* [17].

The aim of this study was to determine whether Arabidopsis homologues of NuA4 subunits are organized into a big protein complex similar to yeast NuA4 or human TIP60-p400. Our initial hypothesis was that PIE1 serves as a platform protein for a plant analog of the human TIP60-p400 complex. Through analysis of proteins that bind to Arabidopsis homologues of ARP4 and SWC4, common subunits of yeast NuA4 and SWR1-C, we confirmed their interaction with PIE1 and other Arabidopsis homologues of NuA4 and SWR1-C subunits. In addition, we revealed their association with an uncharacterized EAF1-like protein, not previously considered a subunit of plant NuA4 or SWR1-C. Subsequently, we focused on a possible role of AtEAF1 as a platform of the plant NuA4 complex. We demonstrated that one of the two isoforms of AtEAF1 interacts with AtYAF9A and AtYAF9B through a conserved HSA domain. Phenotypical analyses of mutants indicated that AtEAF1 and AtYAF9 are necessary for proper timing of transition to flowering, which can be explained by their influence on the H4K5 acetylation in the chromatin of major flowering regulator genes and their transcriptional activity, supporting the role of AtEAF1 as a platform subunit in the plant NuA4 complex.

## Results

### An uncharacterized plant-specific domain-relative of the yeast EAF1 protein is physically associated with AtARP4 and AtSWC4

We used affinity purification followed by tandem mass spectrometry (AP-MS/MS) to test which Arabidopsis homologues of yeast NuA4 and SWR1-C are associated with the common protein module of the two complexes. For this purpose we chose Arabidopsis homologues of ARP4 and SWC4 as protein baits, because these subunits are encoded by single genes in *A. thaliana*. We overexpressed AtARP4 and AtSWC4 fused to Strep-tag, in an Arabidopsis cell suspension culture. Following purification of proteins bound to the baits, we identified them by mass spectrometry. Proteins detected in control purifications, with no bait or with Strep-GFP as bait, were eliminated as nonspecific hits (Additional file 1). Next, we looked for proteins showing sequence- or domain architecture-similarity to subunits of NuA4 and SWR1-C. We also looked for homologues of INO80 complex subunits, because the yeast and human versions of this complex also contain ARP4 [18,19]. As expected, we found conserved subunits of all three complexes in association with AtARP4, while AtSWC4 copurified only with subunits of NuA4 and SWR1-C (Table 1). In agreement with a recently published study [20], we found multiple subunits of the SWI/SNF complex among proteins copurified with AtARP4, suggesting that besides NuA4, SWR1-C and INO80, AtARP4 interacts with SWI/SNF in plants. We identified no subunits characteristic of INO80 or SWI/SNF among the proteins copurified with AtSWC4, which additionally confirms the specificity of the detected interactions.

**Table 1:** ***A thaliana*** **homologues of yeast SWR1**-**C and NuA4 subunits copurify with AtARP4 and AtSWC4**

**Purified proteins**		**Homologues**		**Baits**		**Protein complexes**			**Protein domains**		
**Locus ID**	**Name**	***H. sapiens***	***S. cerevisiae***	**At ARP4**	**At SWC4**	**SWR1** **/** **NuA4**	**INO80**	**SWI** **/** **SNF**-**type**	**HSA**	**ATPase**	**SANT**
AT1G18450	AtARP4^3^	BAF53A	ARP4	+	+	+	+	+			
AT5G22330	RVB1	RUVBL1	RVB1	+	+	+	+				
AT5G67630	RVB21	RUVBL2	RVB2	+	+	+	+				
AT3G49830	RVB22	RUVBL2	RVB2	+	+	+	+				
**AT3G24880**	**AtEAF1A**	**-**	**EAF1**	**+**	**+**	**+**			**+**		**+**
**AT3G24870**	**AtEAF1B**	**-**	**EAF1**	**+**	**+**	**+**			**+**		**+**
AT4G14385	EAF6	MEAF6	EAF6	+	+	+					
AT1G79020	EPL1B	EPC1	EPL1	+	+	+					
AT5G64610	HAM1^1^	TIP60	ESA1	+	+	+					
AT1G54390	ING2	ING3	YNG2	+	+	+					
AT3G12810	PIE1^1^	p400	SWR1	+	+	+			+	+	+
AT2G47210	AtSWC4^1^	DMAP1	SWC4	+	+	+					+
AT5G45600	AtYAF9A^1^	GAS41	YAF9	+	+	+					
AT3G33520	ARP6^1^	ARP6	ARP6		+	+					
AT4G37280	MRG1^1^	MRG15	EAF3		+	+					
AT1G26470	EAF7	MRGBP	EAF7		+	+					
AT1G16690	EPL1A	EPC1	EPL1		+	+					
AT2G36740	SWC2^1^	YL-1	SWC2		+	+					
AT5G37055	SWC6^1^	ZNHIT1	SWC6		+	+					
AT4G36080	TRA1	TRRAP1	TRA1		+	+					
AT2G18000	AtYAF9B^1^	GAS41	YAF9		+	+					
AT3G12380	ARP5^2^	ARP5	ARP5	+			+				
AT5G43500	ARP9	ARP8	ARP8	+			+				
AT3G57300	INO80^2^	INO80	INO80	+			+		+	+	
AT5G13950	NFRKB	NFRKB	-	+			+				
AT1G65650	UCH2	UCH37	-	+			+				
AT5G16310	UCH2	UCH37	-	+			+				
AT4G06634	YY1	YY1	-	+			+				
AT3G60830	ARP7^3^	-	ARP7	+				+			
AT3G17590	BSH^3^	INI1	SNF5	+				+			
AT2G46020	BRM^3^	BRG1/HBRM	SNF2/STH1	+				+	+	+	
AT2G28290	SYD^3^	BRG1/HBRM	SNF2/STH1	+				+	+	+	
AT3G06010	MINU1	BRG1/HBRM	SNF2/STH1	+				+	+	+	
AT5G19310	MINU2	BRG1/HBRM	SNF2/STH1	+				+	+	+	
AT2G47620	SWI3A^3^	BAF155/BAF170	SWI3	+				+			+
AT2G33610	SWI3B^3^	BAF155/BAF170	SWI3	+				+			+
AT1G21700	SWI3C^3^	BAF155/BAF170	SWI3	+				+			+
AT4G34430	SWI3D^3^	BAF155/BAF170	SWI3	+				+			+
AT3G01890	SWP73A	BAF60A	SNF12	+				+			
AT5G14170	SWP73B	BAF60A	SNF12	+				+			

Proteins copurified with AtARP4 or AtSWC4, fused to Strep-tag, were identified by MS/MS and manually annotated on the basis of sequence- and domain architecture-similarity to yeast and human proteins. The table is limited to proteins that were identified as subunits of chromatin remodeling or histone modifying complexes (multiple subunits were identified). Columns on the right show the distribution of selected protein domains.

^1,2,3^Proteins either directly or indirectly linked to *Arabidopsis* SWR1 and/or NuA4 (1), INO80 (2) or SWI/SNF-type (3) complexes, according to published experimental data.

**Figure 1:**
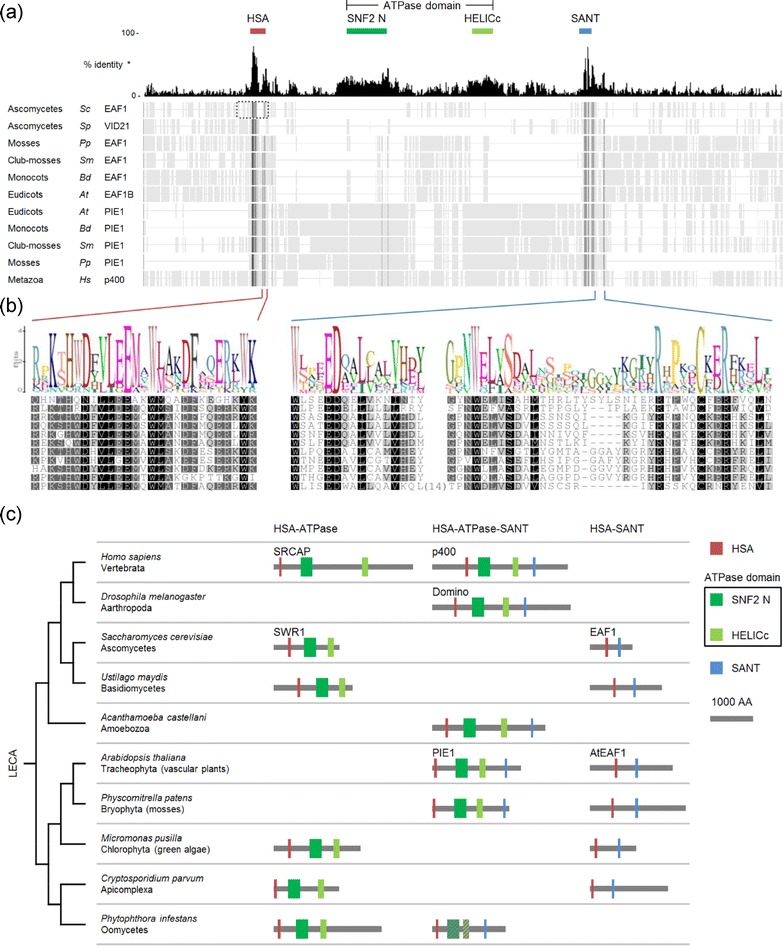
***Arabidopsis thaliana*** **AtEAF1 and PIE1 represent two distinct protein families that share highly similar HSA and SANT domains and do not coexist outside plants. (a)** Protein alignment of domain relatives of *Arabidopsis thaliana* (*At*) AtEAF1 and PIE1, found in *Saccharomyces cerevisiae* (*Sc*), *Schizosaccharomyces pombe* (*Sp*), *Brachypodium distachyon* (*Bd*), *Selaginella moellendorffii* (*Sm*), *Physcomitrella patens* (*Pp*) and *Homo sapiens* (*Hs*). A dotted rectangle depicts the region of yeast EAF1 that recruits ARP4 and actin, according to Szerlong et al. [28]. Columns containing over 75% gaps were removed from the alignment for clarity. Similarity shading of the alignments in **(a)** and **(b)** was done using the Blosum62 matrix. White indicates < 60% similarity, light grey 60 to 80%, dark grey 80 to 100% and black 100%. **(b)** A close-up of the conserved fragments of the HSA domain and the SANT domain. **(c)** Protein domains HSA, ATPase and SANT form three different domain architectures, which occur in various combinations across eukaryotes. All sequences and alignments used in this figure can be found in the Additional file 3. * Mean pairwise identity over all pairs in the column. A sliding window of 5 was used in the histogram.

According to published data for other species, the presence of ARP4 and SWC4 subunits is restricted to protein complexes closely related to NuA4, SWR1-C and TIP60-p400. Therefore, we considered proteins copurified with both baits as potential subunits of a hypothetical plant TIP60-p400-like complex (Table 1). One uncharacterized protein in this category had a domain architecture similar to that of yeast EAF1 (Figure 1a). We therefore named it AtEAF1.

We aligned the amino acid sequences of AtEAF1 homologues identified in distantly related plant species with the sequences of *S. cerevisiae* EAF1 and its *Schizosaccharomyces pombe* homologue VID21, as well as with human p400 and several plant homologues of PIE1 (Figure 1a). Strikingly, all proteins contained highly conserved HSA and SANT domains (Figure 1b), despite little overall sequence similarity and different domain arrangements (Figure 1c).

### AtEAFf1 is encoded by two nearly identical genes that are both transcriptionally active in *A. thaliana*

AtEAF1 is encoded by two genes in all of the sequenced *A. thaliana* ecotypes, except Mt-0 (1001genomes.org). The two genes occupy adjacent loci At3g24870 and At3g24880 on the reverse strand of chromosome 3 and share 98.5% identity in their coding regions. As no full-length cDNA clone was available for AtEAF1, we designed primers using existing annotations (Additional file 2), and cloned the full-length coding sequence (CDS) (Additional file 3). Our *AtEAF1B* CDS clone corresponds to the At3g24870.1 gene model (Additional file 4).

To determine whether both genes were transcriptionally active, we designed three pairs of primers complementary to both coding sequences. Each amplicon contained a restriction site specific for one gene (i.e. not found in the other). The results of restriction digestion of the RT-PCR products were consistent for all three amplicons and indicated that both transcripts are equally abundant in mature rosette leaves (Additional file 4).

### Plant homologues of the NuA4 complex subunit YAF9 interact with AtSWC4 and AtEAF1

**Figure 2:**
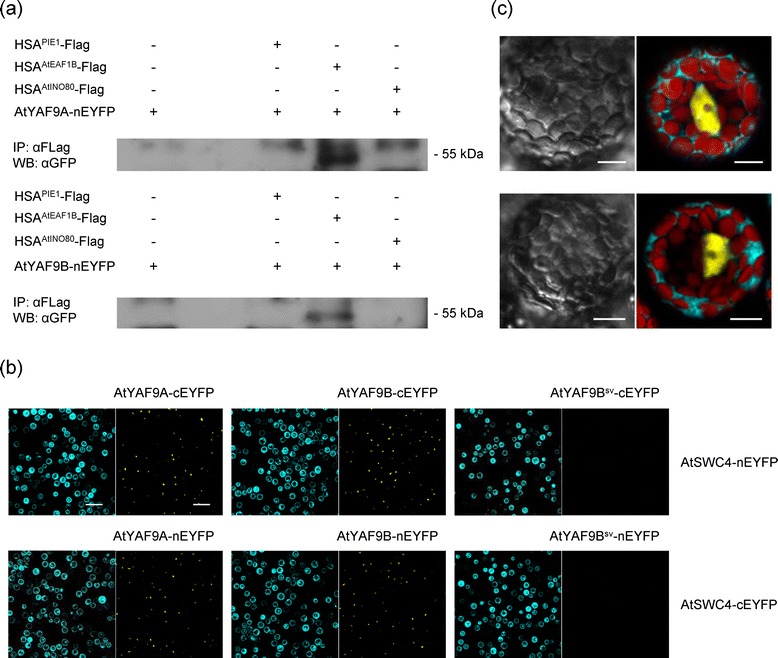
**AtYAF9A and AtYAF9B interact with AtSWC4 and the HSA**-**containing fragment of AtEAF1. (a)** Coimmunoprecipitation test of interaction between AtYAF9A and AtYAF9B with the HSA-containing fragments of PIE1, AtEAF1 and AtINO80. Yaf9:nEYFP fusion proteins were detected with antibodies against GFP. Bands above the 55 kDa bars are nonspecific signals from immunoglobulin heavy chains. For additional experimental controls, see Additional file 5. **(b)** BiFC assay in Arabidopsis mesophyll protoplasts. Each co-transfection consisted of a pair of complementary BiFC constructs (nEYFP and cEYFP fusion, right panels, yellow dots indicate fluorescence complementation in the nuclei) and an ECFP construct as an internal transfection control (left panels, cyan). AtYAF9B^sv^ is the short splice variant of AtYAF9B, lacking the putative C-terminal SWC4-binding domain. **(c)** Enlarged images of single protoplasts showing nuclear localization of the EYFP fluorescence, indicating interaction between AtSWC4 and AtYAF9A (upper image) or AtYAF9B (lower image). Chloroplast autofluorescence is shown in red and ECFP fluorescence in cyan. Scale bar: 10 μm. Contrast and brightness were enhanced in all micrographs in **(b)** and **(c)** to improve clarity.

As shown above, we found evidence for physical association of AtARP4 and AtSWC4 with AtEAF1, a domain relative of yeast NuA4 subunit EAF1. We assumed that AtARP4 and AtSWC4 associated with AtEAF1 through interaction with the latter’s HSA domain. Cooperative binding of ARP4 and SWC4 to the N-terminal region of SWR1, containing the HSA domain, requires YAF9 in yeast [7]. One of the two Arabidopsis homologues of YAF9 was shown recently to be required for histone H4 acetylation in the *FLC* locus, in line with its role as a plant NuA4 subunit [16]. To see whether the HSA domain of AtEAF1 can recruit *A. thaliana* YAF9, we transiently coexpressed nEYFP-tagged AtYAF9A or AtYAF9B with the HSA-containing fragment of AtEAF1 fused to the Flag-tag in Arabidopsis mesophyll protoplasts. Coimmunoprecipitation showed that AtYAF9A and AtYAF9B do indeed interact with this fragment of AtEAF1 (Figure 2a, Additional file 5). We also tested HSA-containing fragments of PIE1 and AtINO80 fused to Flag-tag, but no interaction was observed.

### AtYAF9A and AtYAF9B interact with AtSWC4 in the nucleus

Our AP-MS/MS results revealed a physical association of AtARP4 and AtSWC4 with AtYAF9A and AtYAF9B. To test whether AtARP4, AtSWC4, AtYAF9A and AtYAF9B associate closely to form a functional module, we screened these proteins for protein-protein interactions, using Bimolecular Fluorescence Complementation (BiFC) in Arabidopsis mesophyll protoplasts. Coexpression of AtYAF9A or AtYAF9B fused to the N- or C-terminal fragment of Enhanced Yellow Fluorescent Protein (EYFP) with AtSWC4 fused to the complementary EYFP fragment produced strong fluorescence localized in the nucleus (Figure 2b,c). No other combination of complementary fusions produced detectable EYFP fluorescence (Additional file 6).

Although pairwise sequence alignment between AtYAF9A and AtYAF9B amino acid sequences shows a high degree of similarity along their whole length, only a shorter splice variant of AtYAF9B has been studied previously [16,21]. We obtained CDS clones of both splice variants (Additional file 3, Additional file 4) and found that the shorter variant lacks the conserved C-terminal region, responsible for SWC4 binding in yeast [22]. In agreement with this observation, the shorter splice variant of AtYAF9B did not interact with AtSWC4 when tested by BiFC (Figure 2b). Therefore, we used the longer splice variant in all protein-protein interaction assays described herein.

### AtEAF1B, AtYAF9A and AtYAF9B are necessary for normal *FLC* levels and timing of flowering

**Figure 3:**
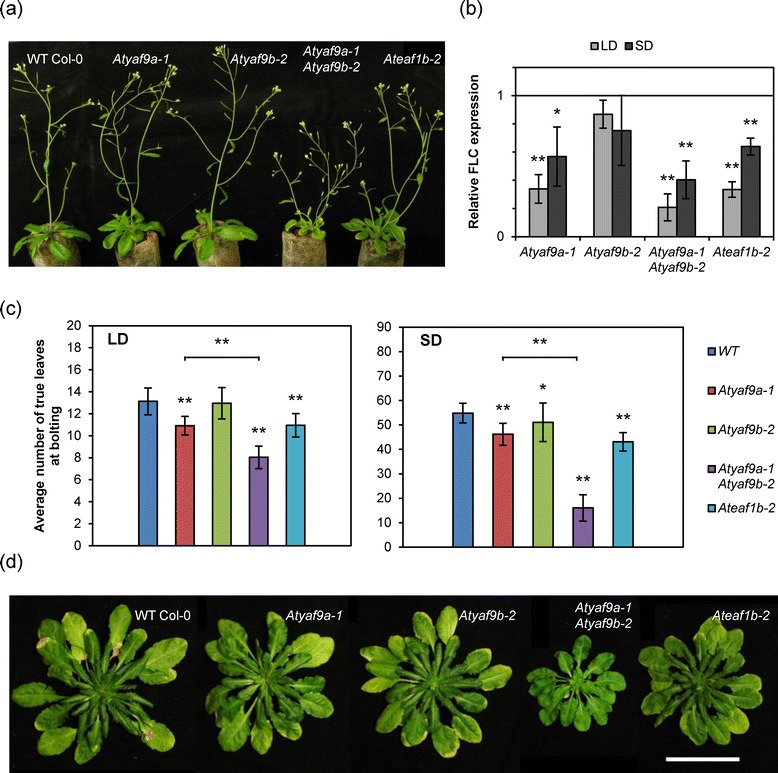
**Mutations in** ***AtEAF1*** **and** ***AtYAF9*** **genes affect** ***FLC*** **expression and flowering time. (a)** Comparison of plants grown under long day conditions (LD). **(b)** Relative expression levels of *FLC* transcript compared to WT control. Seedlings were collected in the middle of the light photoperiod. **(c)** Comparison of flowering time represented by an average number of true rosette leaves at the stage where the flower stem is 1 cm long. **(d)** Representative rosettes of plants grown under short day conditions (SD) at the stage when the leaves were counted. The bar length is 5 cm. In all graphs, asterisks indicate statistical significance of the difference between each mutant and the WT control. A single asterisk indicates a p-value < 0.05, double asterisk – p-value < 0.01 (t-test).

AtYAF9A-deficient plants express *FLC* at reduced levels [16]. Physical interaction between AtYAF9A and AtEAF1, and their similarity to functional counterparts in yeast, YAF9 and EAF1, respectively, suggested that AtEAF1 might also influence *FLC* transcription. To test this prediction we used plants with a T-DNA insertion near the 5′ end of the last exon of the *AtEAF1B* gene (Figure 3a, Additional file 4). We will refer to this line as *Ateaf1b*-*2*. Compared to wild-type seedlings, *Ateaf1b*-*2* mutants expressed *FLC* at significantly reduced levels under both long day (LD) and short day (SD) conditions (Figure 3b).

In our protein-protein interaction assays both AtYAF9A and AtYAF9B interacted with AtSWC4 and AtEAF1 (Figure 2). To investigate whether AtYAF9B also contributes to *FLC* expression and whether there is a redundancy of AtYAF9A and AtYAF9B function, we compared the relative *FLC* expression levels of *Atyaf9a*-*1*, *Atyaf9b*-*2* and *Atyaf9a*-*1 Atyaf9b*-*2* mutants with wild-type plants using the same experimental setup as described for *Ateaf1b*-*2* above. Interestingly, the *FLC* expression in the double mutant was not significantly reduced when compared to *Atyaf9a*-*1* (LD p = 0.18, SD p = 0.33) (Figure 3b).

A decrease in *FLC* expression leads to earlier transition from vegetative to reproductive phase in the *Atyaf9a*-*1* mutant [16]. We tested the effect of reduced *FLC* expression on the timing of flowering in AtYAF9- and AtEAF1-deficient plants. We grew *Atyaf9a*-*1*, *Atyaf9b*-*2*, *Atyaf9a*-*1 Atyaf9b*-*2* and *Ateaf1b*-*2* mutants, as well as wild type plants, under LD and SD conditions. The *Atyaf9a*-*1 Atyaf9b*-*2* double mutant flowered significantly earlier than single mutants under both LD and SD conditions (Figure 3c,d). Importantly, both *Ateaf1b*-*2* and *Atyaf9a*-*1* mutants flowered at a similar growth stage, which was intermediate between that of the *yaf9* double mutant and wild-type plants (WT), while the early flowering phenotype of the *Atyaf9b*-*2* mutant was least pronounced of all the mutant lines (Figure 3c).

### AtEAF1 and AtYAF9 are necessary for normal levels of H4K5 acetylation

**Figure 4:**
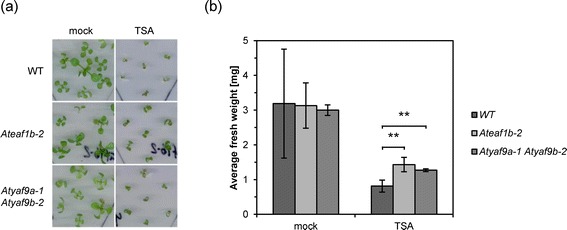
***Ateaf1b***-***2*** **and** ***Atyaf9a*** **-** ***1 Atyaf9b*** **-** ***2*** **mutants gain increased resistance to TSA. (a)** 12 day-old seedlings grown in the presence of TSA or mock. All images are in the same scale. **(b)** Comparison of average fresh weight between plants treated with TSA or mock. Error bars represent standard deviation of 4 biological replicates. Double asterisks indicate a p-value < 0.01 (t-test).

Published experimental data support involvement of Arabidopsis homologues of NuA4 subunits YAF9 and ESA1 (HAM1 and HAM2) in the acetylation of lysine 5 of histone H4 [13]. Therefore, if AtEAF1 is a functional subunit of the Arabidopsis NuA4 complex, partial loss of its function should lead to changes in H4K5 acetylation levels. As an initial test of the influence of AtEAF1 on H4K5 acetylation, we grew *Ateaf1b*-*2* and *Atyaf9a*-*1 Atyaf9b*-*2* seedlings for 12 days on MS medium supplemented with 1% sucrose or 1% sucrose and 12.5 μM Trichostatin A (TSA) (Figure 4). TSA is a specific inhibitor of histone deacetylases and has a strong negative effect on plant growth, coinciding with dramatic accumulation of acetylated histones [23,24]. We reasoned that impaired function of an important histone acetylatransferase such as NuA4 could prevent abnormal accumulation of acetylated histones and give mutant plants an advantage over WT plants under TSA challenge. As expected, average fresh weight of *Atyaf9a*-*1 Atyaf9b*-*2* and *Ateaf1b*-*2* mutant plants grown on plates containing TSA was significantly larger than those of WT plants grown in the same conditions, whereas no significant differences where observed under control conditions (Figure 4b). In order to verify if the observed effect can be attributed to differences in global H4K5 acetylation levels, we tested the abundance of histone H4 acetylated on lysine 5 by Western Blot (Additional file 7). Only the double mutant *Atyaf9a*-*1 Atyaf9b*-*2* displayed decreased levels of acetylated H4K5 relative to WT which indicates that the increased resistance of *Ateaf1b*-*2* plants to TSA cannot be due to a global loss of H4K5 acetylation. This observation could be explained if AtEAF1 had a specialized function in the Arabidopsis NuA4 complex. In fact, in yeast *eaf1* mutant a strong decrease in histone H4 acetylation was observed in the promoter region of the PHO5 gene, but no decrease in bulk histone H4 acetylation was reported [5]. Therefore we decided to test if specific genomic targets of histone acetyltraferases are affected in plants with impaired function of AtEAF1. We focused on major regulators of flowering transition *FLC*, *FT*, *CONSTANS* (*CO*) and *SUPPRESSOR OF OVEREXPRESSION OF CONSTANS 1* (*SOC1*) as histone acetylation in these genes was found to be deregulated by various H4 acetylation mutants in previous studies [15-17]. Our observations of flowering timing in *Ateaf1b*-*2* mutant, presented above, further justified that choice.

**Figure 5:**
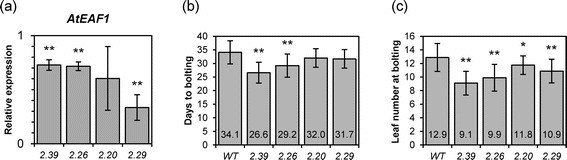
**Plants with transcript levels of** ***AtEAF1*** **decreased by artificial miRNA show deregulation of flowering time. (a)** Expression levels of *AtEAF1* in four independent amiRNA lines relative to WT Col-0. **(b)** Comparison of flowering time represented by an average number of days until the stage where the flower stem is 1 cm long. **(c)** Comparison of flowering time represented by an average number of true rosette leaves at the stage where the flower stem is 1 cm long. Asterisks in **(a, b, c)** indicate a p-value < 0.05 or p-value < 0.01 (double asterisk) (t-test).

In order to verify if the flowering phenotype of the *Ateaf1b*-*2* mutant is related to the function of AtEAF1, we generated transgenic Arabidopsis plants in which transcript levels of *AtEAF1A* and *AtEAF1B* genes were reduced simultaneously through artificial micro RNA (amiRNA) (Figure 5a) [25]. Although independent transgenic lines expressing amiRNA construct displayed a moderate early flowering phenotype (Figure 5b, c), it did not correlate with the decrease in *AtEAF1* transcript (Figure 5), In the line 2.39 which showed the earliest flowering we detected only slightly decreased levels of *AtEAF1* transcript while line 2.29, with stronger *AtEAF1* silencing, showed less obvious early flowering phenotype.

**Figure 6:**
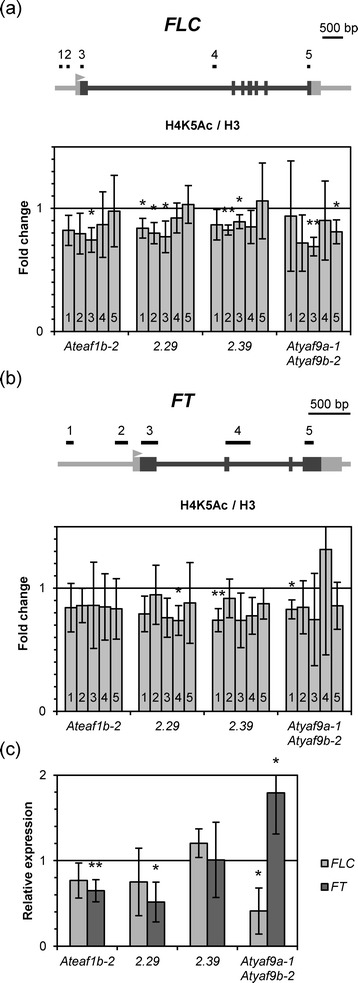
***Ateaf1b*** **-** ***2***, ***Atyaf9a*** **-** ***1 Atyaf9b*** **-** ***2*** **and** ***AtEAF1*** **-** **amiRNA lines display reduced H4K5 acetylation but different activity of** ***FLC*** **and** ***FT.*** **(a**, **b)** Acetylation levels in different parts of the *FLC* and *FT* genes normalized to H3 presented as fold change over WT Col-0. **(c)** Expression levels of *FLC* and *FT* relative to WT Col-0. Asterisks in **(a, b, c)** indicate a p-value < 0.05 or p-value < 0.01 (double asterisk) (t-test).

**Figure 7:**
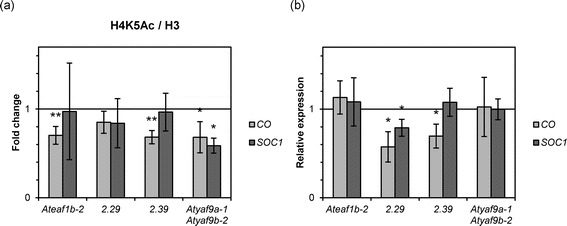
***CO*** **is more affected than** ***SOC1*** **in** ***Ateaf1b***-***2*** **and** ***AtEAF1***-**amiRNA lines. (a)** H4K5 acetylation levels in the promoters of *CO* and *SOC1* normalized to H3 presented as fold change over WT Col-0. **(b)** Expression levels of *CO* and *SOC1* relative to WT Col-0. Asterisks in **(a, b)** indicate a p-value < 0.05 or p-value < 0.01 (double asterisk) (t-test).

Our next step was to characterize differences in histone H4K5 acetylation levels over *FLC* and *FT* genes between WT, *Ateaf1b*-*2*, 2.29, 2.39 and *Atyaf9a*-*1 Atyaf9b*-*2* plants by chromatin immunoprecipitation (ChIP). We carried out the experiments on 10-day old seedlings grown under long day conditions, collected at the end of the day. For the amplification of the DNA fragments obtained from ChIP we used five pairs of PCR primers for each gene, corresponding to various functional elements of the gene (Figure 6a, b, Additional file 2). We observed a moderate but consistent decrease in H4K5 acetylation over both genes. Interestingly, we observed a stronger reduction in acetylation levels near the 5′ end of the genes, especially in the *FLC* locus. Following this observation, we tested the acetylation levels in the promoter regions of two other major flowering regulators, *CO* and *SOC1*. We observed a significant and consistent drop of H4K5 acetylation in the promoter of *CO* but little change in SOC1, except for the *Atyaf9a*-*1 Atyaf9b*-*2* line, which showed significantly lower acetylation at both loci (Figure 7a).

Examination of the transcript levels of *FLC*, *FT*, *CO* and *SOC1* genes by RT-qPCR showed their transcriptional deregulation. As expected, in many cases reduction in H4K5 acetylation was accompanied by a decreased level of a given transcript (Figure 6c, Figure 7b). In several cases, however, the observed decrease in acetylation did not result in lower transcript levels. In fact, we observed higher relative levels of *FT* transcript in the *Atyaf9a*-*1 Atyaf9b*-*2* line, despite a significant decrease of H4K5 acetylation in the promoter region of *FT* (Figure 6b,c). The lack of clear correlation between H4K5 acetylation and transcriptional activity of tested genes may be at least partially explained by their involvement in a network of functional interactions involving other mechanisms of transcriptional regulation.

## Discussion

In this study, we have investigated the role of a previously uncharacterized protein AtEAF1 as a potential subunit of the plant NuA4 histone acetylatransferase complex. We found that AtEAF1 and other Arabidopsis homologues of NuA4 subunits copurify with Arabidopsis homologues of ARP4 and SWC4, common subunits of the yeast NuA4 and SWR1 complexes. Our AtARP4 and AtSWC4 purifications resulted also in detection of peptides belonging to the subunits of the Arabidopsis SWR1 complex including PIE1, which recruits subunits responsible for H2A.Z deposition in Arabidopsis [26,27]. So far, PIE1 has been the only known plant protein with a potential to physically link AtARP4 and AtSWC4 with homologues of other SWR1-C and NuA4 subunits. We argue that the identification of AtEAF1 opens a possibility for a PIE1-independent NuA4 complex formation in plants.

The observed physical association of AtEAF1 with AtAPR4 and AtSWC4 is best explained by the presence of the HSA domain in AtEAF1. HSA domain is a common feature of the platform subunits of SWR1-C, NuA4 and the hybrid complex TIP60-p400. Several published studies suggest that HSA domain may provide the assembly surface for a submodule consisting of ARP4, SWC4, YAF9 and monomeric actin in NuA4 and SWR1 complexes [5,28,29]. Indeed, by coimmunoprecipitation we demonstrated a physical interaction between both Arabidopsis YAF9 homologues and a fragment of AtEAF1 containing the HSA domain (Figure 2a). Formation of a protein module by Arabidopsis homologues of ARP4, SWC4 and YAF9 is further supported by the interaction of AtSWC4 with AtYAF9A and AtYAF9B, as revealed by BiFC (Figure 2b,c), and by reciprocal copurification of AtARP4 and AtSWC4, as shown by AP-MS/MS (Table 1). Interestingly, we were not able to detect the expected interaction between YAF9 homologues and the fragment of PIE1 containing the HSA domain in our CoIP assay (Figure 2a). As YAF9 is necessary for H2A.Z deposition in yeast [5,30], it is assumed to be a subunit of the Arabidopsis SWR1-C. Although our CoIP result alone is not sufficient to question this view, it seems to agree with a relatively mild phenotype of the *Atyaf9a*-*1 Atyaf9b*-*2* mutant (Figure 3) as compared to the *pie1*-*5* or *arp6*-*1* mutant phenotypes [11].

The other conserved sequence feature of AtEAF1 is the SANT domain, located C-terminal of the HSA domain. We found that the combination of HSA and SANT domains is usually represented by no more than two genes per eukaryotic genome (Figure 1c). For example, in both *S. cerevisiae* and human there is just single gene that encodes an HSA-SANT domain protein, i.e. EAF1 and p400, respectively. Importantly, AtEAF1, unlike p400 or PIE1, does not contain an ATPase domain between the HSA and SANT domains. This characteristic leaves EAF1 as the most probable functional analogue of AtEAF1.

Physical interaction of AtEAF1 with AtYAF9A and AtYAF9B is consistent with our observation that plants carrying a T-DNA insertion in one of the *AtEAF1* genes are phenotypically similar to *Atyaf9a*-*1* mutants. It has been shown recently that *Atyaf9a*-*1* mutants display reduced levels of H4 acetylation in *FLC* gene chromatin [16], which leads to a decrease in *FLC* transcript levels and, consequently, partial loss of flowering inhibition. Although the double mutant *Ateaf1a Ateaf1b* is not currently available, our results show that the single mutant *Ateaf1b*-*2* is affected in *FLC* expression and flowering time under LD and SD conditions (Figure 3), phenocopying *Atyaf9a*-*1*.

We demonstrated that AtEAF1 and SWC4 interact with both Arabidopsis homologues of YAF9. If the influence of AtYAF9A on *FLC* expression results from its interaction with AtEAF1, the same could be true for AtYAF9B. Functional redundancy of AtYAF9A and AtYAF9B has been suggested previously on the basis of the phenotype of *Atyaf9a*-*1 Atyaf9b*-*kd* plants, which differs from the phenotype of either *Atyaf9a*-*1* or *Atyaf9b*-*kd* plants [21]. Our own data show that a double mutant, carrying T-DNA insertions in both YAF9 genes, displays stronger deregulation of flowering time control than either of the single mutants (Figure 3). This result suggests some level of functional redundancy between the two genes in the Arabidopsis NuA4 complex and is in agreement with our finding that both proteins interact with AtEAF1B.

Our analyses of the influence of the histone deacetylase inhibitor TSA revealed an increased resistance to this hyperacetylation-inducing agent in *Atyaf9a*-*1 Atyaf9b*-*2* and *Ateaf1b*-*2* mutant seedlings. This result supports a role for AtYAF9A, AtYA9B and AtEAF1B in histone acetylation. Under TSA treatment *Atyaf9a*-*1 Atyaf9b*-*2* accumulated less acetylated H4K5 than WT plants (Additional file 7) which may explain its increased resistance to the drug. No such effect was observed for *Ateafb*-*2* mutant. Several explanations of this result are possible. We chose to follow a hypothesis that AtEAF1 is only required for H4K5 acetylation in specific genomic targets, similar to yeast EAF1 which is mainly required for the NuA4 activity in the promoter regions [31].

As we were not able to determine which genes may be relevant to the negative effect of TSA on plant growth, we used genes encoding main flowering regulators as models to study the role of AtEAF1 in Arabidopsis NuA4. ChIP experiment, in which we employed artificial miRNA knock-down lines for *AtEAF1* gene showed that decreased levels of *AtEAF1* transcript have similar effect on histone H4K5 acetylation as the disruption of one of the AtEAF1 isoforms in the *Ateaf1b*-*2* mutant (Figures 6 and 7). As expected, we observed a general decrease in H4K5 acetylation levels over most of the tested regions of *FLC* and *FT* loci with a slight bias towards their 5′ end (Figure 6a,b). We could also detect significant decrease in acetylation in the promoter of the *CO* gene and a weaker effect in the *SOC1* gene (Figure 7a).

The gene-specific effects which we observed at the level of histone acetylation were not evenly reflected in the transcript levels of the studied genes. This can be at least in part attributed to the functional interactions between *FLC*, *FT*, *CO*, *SOC1* and multiple other genes involved in the control of flowering transition. Partial loss of NuA4 activity likely affects many genes with diverse functions. Put to such a stress, the system of positive and negative flowering regulators may seek new balance with outcomes that are difficult to predict. Another possible explanation of these results comes from a recent study on plant homologues of the NuA4 subunit EAF3, i.e. MRG1 and MRG2. In contrast to previous reports showing that Arabidopsis homologues of NuA4 subunits YAF9 and ESA1 contribute to flowering time control mainly by ensuring proper levels of the negative flowering regulator FLC, Xu et al. demonstrated that transcriptional activity of the positive flowering regulator *FT* also depends on H4K5 acetylation [17]. According to the report, MRG proteins act as H3K36me3 readers and guide HAM1 and HAM2 acetyltraferases through a direct protein-protein interaction. This does not exclude a role for other NuA4 subunits as in the case of the yeast NuA4 complex or human TIP60-p400 which incorporate MRG homologues as stable subunits [12,32]. In fact, MRG1 was among the NuA4 subunits identified as AtSWC4 interaction partners in our AP-MS/MS analyses (Table 1, Additional file 1). Conservation of the AtEAF1 role as a bridge between functional submodules of NuA4 containing YAF9 and EAF3 homologues would explain the ambivalent effects of its partial loss that we observe.

## Conclusions

Our work introduces AtEAF1 as a new subunit of the Arabidopsis NuA4 complex. Characteristic sequence features, interaction with the AtYAF9-AtSWC4-AtARP4 submodule and influence on H4K5 acetylation suggest that AtEAF1 may be a functional analog of the yeast EAF1 protein. Judging from previous studies on human and yeast models, elucidation of the exact role of AtEAF1 subunit in the structural integrity and function of the Arabidopsis NuA4 complex will require significant effort. Our data do not exclude the existence of another large HAT complex in Arabidopsis, organized around PIE1, with additional H2A.Z exchange activity. Addressing this problem may also be required to better understand the regulation of histone H4 acetylation in plants.

## Methods

### Plant material

The *Ateaf1b*-*2* (SALK_067053), *Atyaf9a*-*1* (SALK_106430) and *Atyaf9b*-*2* (SALK_046223) mutants were obtained from the T-DNA mutant collection at Salk Institute. The seeds were provided by Nottingham Arabidopsis Stock Centre (NASC). Positions of T-DNA insertions were confirmed by Sanger sequencing of cloned PCR amplification products (for primer sequences see Additional file 2). The double mutant *Atyaf9a*-*1 Atyaf9b*-*2* was obtained by crossing the homozygous *Atyaf9a*-*1* and *Atyaf9b*-*2* mutants. WT Columbia-0 ecotype (Col-0) plants were used as a control in all experiments.

### Protoplast preparation and transfection

Protoplasts were prepared from 30-day old plants grown under LD conditions. The Tape-Arabidopsis Sandwich method was used for protoplast preparation [33]. Transfection of protoplasts was carried out in U96 Microwell plates (Thermo Scientific Nunc) according to a published protocol [34].

### Coimmunoprecipitation (CoIP)

For each CoIP, 12 wells in the U96 Microwell plate were used to cotransfect protoplasts with plasmids carrying HSA-Flag and YAF9-nEYFP constructs. Isolation of protoplast proteins and CoIP was performed as previously described [35], except that the immunoprecipitation (IP) lysis buffer was modified by replacing HEPES with 10 mM Tris-HCl, pH 7.5. The same buffer was used to wash beads after IP. Immunodetection after western blot was done with anti-GFP antibodies (sc-8334, Santa Cruz, www.scbt.com) and anti-Flag antibodies (OctA-Probe Antibody D-8, sc-807, Santa Cruz). All CoIPs were carried out at least twice.

### Affinity purification followed by tandem mass spectrometry (AP-MS/MS)

Expression cassettes containing the coding sequences of the One-STrEP-tag:ATARP4 or One-STrEP-tag:ATSWC4 fusion proteins under the control of the Arabidopsis *UBQ10* promoter and *NOS* terminator were ligated into the binary vector pART27 [36]. *Agrobacterium tumefaciens* (strain GV3101) mediated transformations were carried out to stably express recombinant proteins in the Arabidopsis suspension-cultured cells (T87 line).

To purify AtARP4 or AtSWC4 complexes, 320 ml (~24 g of fresh weight) of a 10-day-old T87 culture were vacuum filtered, ground in liquid nitrogen and resuspended in 5 ml of extraction buffer H (200 mM NaCl, 200 mM Tris-HCl pH 8.0, 200 mM NaF, 15% glycerol, 0.3 mM EDTA, 0.5% TritonX-100, 0.8 mM PMSF, 4 mM DTT, 3.2 mM NaV_2_O_3_,) or L (150 mM NaCl, 100 mM Tris-HCl pH 8.0, 100 mM NaF, 10% glycerol, 2.5 mM EDTA, 2.5 mM EGTA, 0.4% Triton X-100, 0.8 μM PMSF, 4 mM DTT, 3.2 mM NaV_2_O_3_) with one tablet of cOmplete EDTA-free Protease Inhibitor Cocktail (Roche, www.roche.com). After centrifugation (15000 *g*, 15 min, 4°C and then 180 000 *g*, 90 min, 4°C) supernatant was loaded onto a column containing 600 μl of Strep-Tactin Superflow High Capacity resin (IBA). After seven washing steps (100 mM Tris-HCl pH 8.0, 150 mM NaCl), bound proteins were eluted with 1.8 ml of 2.5 mM desthiobiotin (IBA) and finally concentrated and desalted using Amicon Ultra 10 K (Millipore, www.millipore.com).

Trypsin–digested peptides were analyzed using Orbitrap Velos (Thermo Scientific, www.thermo.com) coupled with nanoAcquity UPLC (Waters, www.waters.com) according to standard protocols at the Mass Spectrometry Laboratory, Institute of Biochemistry and Biophysics, Polish Academy of Sciences, Warsaw. To identify proteins, Mascot software (Matrix Science, www.matrixscience.com) was employed to search mass spectra against the TAIR10 database using the following parameters: no missed cleavages allowed, 20 ppm for peptide mass tolerance and 0.6 Da for fragment ion mass tolerance, fixed modification – cysteine carbamidomethylation, variable modification – methionine oxidation. Results were filtered using significance threshold p-value < 0.05 and expect cut-off 0.05 (http://www.matrixscience.com/help/scoring_help.html).

*A. thaliana* T87 suspension-cultured cells were grown at 22°C under continuous light (50 μmol m^−2^ s^−1^) and shaking at 120 rpm in Gamborg’s B5 medium [37] with 1.5% sucrose and 0.1 μg/l 2,4-dichlorophenoxyacetic acid (Sigma-Aldrich, www.sigmaaldrich.com).

### Bimolecular fluorescence complementation (BiFC)

For BiFC, single wells in the U96 Microwell plate were used to transfect protoplasts with plasmids encoding proteins of interest fused to the N-terminus of nEYFP and cEYFP. Empty vector (3 μg) encoding Enhanced Cyan Fluorescent Protein (ECFP) was added to each well as a transfection control. After transfection, protoplasts resuspended in 200 μl of W5 solution with 5 mM glucose [33] were transferred to black 96-well black glass-bottom plates (SensoPlate, Greiner Bio-One, www.greinerbioone.com), sealed with transparent sealing tape (Thermo Scientific Nunc, cat. no. 236366), and returned to the growth chamber for overnight incubation. After incubation, the sealing tape was removed and the plate was screened with a Nikon A1Rsi confocal system (www.nikon.com). See Additional file 8 for original files.

### Gene expression analyses

RNA was extracted using TRI Reagent (Sigma-Aldrich). For the analysis of *FLC* expression, RNA was extracted from 10- and 16-day old seedlings, grown in plates under LD (16 hours of light) or SD (8 hours of light) conditions, respectively. For the analysis of relative expression levels of AtEAF1A and AtEAF1B, RNA was extracted from mature rosette leaves of Col-0 WT plants grown under LD conditions. The gene expression data presented in Figures 6 and 7 was obtained from plants grown and harvested in the same way as plants grown for ChIP analyses. Reverse transcription was done with the RevertAid kit (Thermo Scientific). Relative expression levels of *FLC* were measured by quantitative PCR. *UBQ10* (Figures 3 and 5) and *UBC21* (Figures 6 and 7) gene transcripts were used as a reference. The 2^-ΔΔCt^ method [38] was used to quantify the relative transcript levels in all experiments. Sequences of all primers can be found in Additional file 2.

### Preparation of genetic constructs

Coding sequences of AtYAF9B, AtEAF1, PIE1 and INO80 were cloned from cDNA obtained from WT Col-0 plants. The remaining coding sequences were subcloned from pUNI51 constructs from the FL-cDNA collection at the Salk Institute, provided by Arabidopsis Biological Resource Center (ABRC). All transient expression vectors used in this study are based on pSAT vector series [39]. The Flag vectors were constructed by replacing the nEYFP sequence with Flag sequence. All vectors were modified by inserting two *Sfi*I recognition sites into the multiple cloning site to facilitate transfer of coding sequences between vectors. Sequences of all primers can be found in Additional file 2.

### Sequence analyses

Conserved protein domains were found using InterProScan [40]. Domain relatives were identified using CDART [41] and by sequence similarity search using BLAST (blast.ncbi.nlm.nih.gov). All multiple sequence alignments were performed using the T-Coffee program [42] accessed through EMBL-EBI web services (www.ebi.ac.uk). For visualization of sequence alignment data, Geneious version 6.0 was used (Biomatters, www.geneious.com).

### Trichostatin A treatment

Plants were grown in plates containing MS medium supplemented with 1% sucrose with pH stabilized by MES at 5.7. DMSO solution of Trichostatin A (Sigma-Aldrich) was added to the medium before pouring the plates to the final concentration of 12.5 μM. Equal amount of DMSO was added as a mock. Seeds were surface-sterilized before sawing and kept in 4°C for four days. After stratification, plates were transferred to a growth chamber with long day conditions (16 h of light). The measurements were done on 12-day old seedlings.

### Artificial micro RNA lines

Artificial micro RNA transgene was designed with the WMD3 tool (wmd3.weigelworld.org) and constructed according to the instructions available at the same website. Briefly, the target-specific sites of the Arabidopsis miRNA precursor MIR319a cloned into pRS300 vector were mutated through overlap extension PCR to target a sequence identical in both *AtEAF1* genes. pRS300 was a gift from Detlef Weigel (Addgene plasmid # 22846). After confirmation of the mutations by DNA sequencing, the precursor was placed downstream of the *UBQ10* promoter in a binary vector using standard molecular biology procedures. The plasmid was then transferred into *Agrobacterium tumefaciens* strain GV3101. *A. thaliana* Col-0 plants were transformed using floral deep method [43]. Transgenic plants were selected on soil by spraying with diluted BASTA (Bayer). Presence of the amiRNA construct was confirmed by PCR. Progeny of the BASTA resistant plants of the T1 and T2 plants was used in subsequent experiments. Sequences of all primers can be found in Additional file 2.

### Chromatin immunoprecipitation (ChIP)

About 350 mg of 10-day-old seedlings grown in LD conditions were harvested at the end of the light photoperiod, ground in liquid nitrogen and then fixed for 15 min at room temperature in extraction buffer containing 1% formaldehyde (60 mM Hepes pH 8.0, 1 M sucrose, 5 mM KCl, 5 mM MgCL_2_, 5 mM EDTA, 0.6% Triton X-100, 1% formaldehyde, 1 mM PMSF, 1% Protease Inhibitor Cocktail, Sigma). The reaction was quenched by adding glycine to a final concentration of 100 mM. The homogenate was filtered through Miracloth, centrifuged and the pellet was washed with buffer containing 0.25 M sucrose, 10 mM Tris-HCl pH 8.0, 10 mM MgCl2, 1% Triton X-100, 1 mM EDTA, 5 mM beta-mercaptoethanol, 1 mM PMSF and Protease Inhibitor Cocktail. Then, the pellet was resuspended in the sonication buffer (50 mM Tris-HCL pH 8.0, 10 mM EDTA, 0.5% SDS, 1 mM PMSF and Protease Inhibitor Cocktail) and sonicated using Bioruptor (Diagenode) for 25 cycles of 30 sec on and 30 sec off. Sonicated chromatin was diluted 10-fold and incubated with antibodies (anti-H3, Abcam, ab1791; anti-H4K5ac, Millipore 07-327) overnight at 4°C with gentle agitation. After pre-clearing, magnetic protein A-beads (Dynabeads protein A, Life Sciences) were incubated with the antibodies-chromatin mix for 1 hours. The slurry was then washed and DNA was extracted with 10% Chelex (Biorad) as described previously [44].

All ChIP experiments were carried out with three independent biological replicates and quantified by qPCR (Maxima SybrGreen/ROX, Thermo Scientific). Primer sequences used for the ChIP-qPCR are listed in Additional file 2. The ChIP data are presented as the ratio of percent of input for H4K5ac to percent of input for total H3 normalized to WT levels. Error bars correspond to SD of the mean of three biological replicates. Statistical evaluations were performed using a Student’s t-test.

## Additional files

Additional file 1:
**AP-MS/MS data.**
Additional file 2:
**Oligonucleotides used in this study.**
Additional file 3:
**DNA and protein sequences, alignments.**
Additional file 4:
**Gene structures of** ***AtYAF9A*** **,** ***AtYAF9B*** **and** ***AtEAF1B*** **with positions of T-DNA insertions in the mutant lines marked.** Details of the amiRNA design and phenotypes of the silenced lines. Relative expression levels of the *AtEAF1A* and *AtEAF1B* genes. Additional file 5:
**Uncropped scans of the Western blot films.**
Additional file 6:
**BiFC screening for protein-protein interactions between AtARP4, AtSWC4, AtYAF9A and AtYAF9B.**
Additional file 7:
**Western Blot analyses of bulk H4K5 acetylation levels in mutants and WT plants treated or untreated with TSA.**
Additional file 8:
**Original confocal images (.nd2 format requires free viewer available at** www.nikoninstruments.com/Products/Software/NIS-Elements-Viewer **).**
