# Supplementary material for: AtEAF1 is a potential platform protein for Arabidopsis NuA4 acetyltransferase complex
Source: BMC Plant Biol. 2015 Mar 5;15:75. doi: 10.1186/s12870-015-0461-1 (PMC4358907; doi:10.1186/s12870-015-0461-1)
Supplement: Additional file 2: — Oligonucleotides used in this study. [file 12870_2015_461_MOESM2_ESM.doc]

**Oligonucleotides used in the this study**

Genotyping

| Line ID | Name | Primer sequence |
| --- | --- | --- |
| Left border primer of the T-DNA insertion (signal.salk.edu) | LBb1.3 | ATTTTGCCGATTTCGGAAC |
| SALK_067053 (*Ateaf1b-2*) | LP | AATTGCATCTGTTGTTGAGGG |
|  | RP | CGATTTCTCACATGTGGGAAC |
| SALK_106430 (*Atyaf9a*) | LP | ACTGGAACAGAGGAGAGAGCC |
|  | RP | ATCAATCTCACAAGTGGGCAG |
| SALK_046223 (*Atyaf9b*) | LP | GCCTCCATCGGCTATACAAAG |
|  | RP | TGGAGTTGTGAGTTTCCCTTG |

**Bimolecular Fluorescence Complementation and Coimmunoprecipitation**

| Gene | Name | Primer sequence | Purpose |
| --- | --- | --- | --- |
| - | uni51-F | CTGTTGGTGTGTCTATTAAATCG | Universal forward primer for subcloning between pUNI51 and pSAT-SfiI vectors |
| AT1G18450 (*AtARP4*) | R | AGGCCCATGAGGCCAAGGGCATTTTCTCTGAATGTA | Gene-specific primer for removing the stop codon from the pUNI51 clone (PCR uni-51-F + R) |
| AT2G47210 (*AtSWC4*) | R | AGGCCCATGAGGCCAGAGATCAGAGGCTTTCAACTT |  |
| AT5G45600 (At*YAF9A*) | R | AGGCCCATGAGGCCACAGGTCTGATCCTGTTTTAAC |  |
| AT2G18000.1 (*AtYAF9B*) | F | AGGCCGTCAAGGCCAGAAGGAGATATAACCATGGAGTCGGATATCGAGATT | Cloning from cDNA |
|  | R | AGGCCCATGAGGCCAGAACAAGAATGCACCTGGAGG |  |
| AT2G18000.2 (*AtYAF9B*) | R | AGGCCCATGAGGCCAACATTCATAGCCAGAGGAAGA |  |
| AT3G24870 (*AtEAF1B*) | F | AGGCCGTCAAGGCCAGAAGGAGATATAACCATGTCAGATGCAGTTATGTTAGATACT | Cloning of a 200 bp fragment of the CDS containing the HSA domain |
|  | R | TGGCCCATGAGGCCAAACTGAATAAAAGAGGCTTGCT |  |
| AT3G12810 (*PIE1*) | F | AGGCCGTCAAGGCCAGAAGGAGATATAACCATGGCGTCTAAAGGTGGTAAATCTA |  |
|  | R | TGGCCCATGAGGCCAACCTGCAGAAGAATTTATCTCT |  |
| AT3G57300 (*AtINO80*) | F | AGGCCGTCAAGGCCAGAAGGAGATATAACCATGGTTTTACAGGTCCATTACGTGAAGA |  |
|  | R | TGGCCCATGAGGCCACTCAGAAGGTTCTGCCGCTGA |  |
| - | F | TCGACAAGGAGATTACAAGGATGACGATGACAAGTGAT | Construction of the pSAT-FLAG vectors |
| - | R | CTAGATCACTTGTCATCGTCATCCTTGTAATCTCCTTG |  |

**Construction of AmiRNA precursor**

| Name | Primer sequence |
| --- | --- |
| miR2 I | GATTGTAACGCCCTTAATCGCGTTCTCTCTTTTGTATTCC |
| miR2 II | GAACGCGATTAAGGGCGTTACAATCAAAGAGAATCAATGA |
| miR2 III | GAACACGATTAAGGGGGTTACATTCACAGGTCGTGATATG |
| miR2 IV | GAATGTAACCCCCTTAATCGTGTTCTACATATATATTCCT |

**RT-PCR, RT-qPCR**

| Gene | Name | Primer sequence |
| --- | --- | --- |
| AT3G24880 / AT3G24870 (*AtEAF1A / AtEAF1B*) | EApaF | TTTGAGGATCAGGCGCTTGT |
|  | EApaR | TGCACTATCAGCCCCATCAC |
|  | EBamF | TTGAGAAGATTTGTTTGATTGGGAAGA |
|  | EBamR | TCTTGACCTGAAGTTGATGCGT |
|  | ETaqF | GTTTGCATGGTGGATCGGC |
|  | ETaqR | GCTTCTTTGCACTATGAGGACC |
|  | F | AAAAAGCAGGCTCCACCATGCATGGAAGCGTTTCAGG |
|  | R | AGAAAGCTGGGTCTTCCACTTTTGTATCAGGAGGC |
|  | F | AACTCCATGATCAGGCCTCG |
|  | R | TCAAATTACTGAAAGCTGGGATCC |
| *FLC* | F | ACTTGAACTTGTGGATAGCAAGC |
|  | R | CCAGTTGAACAAGAGCATCGA |
| *FT* | F | CCAAGTCCTAGCAACCCTCA |
|  | R | TCTCATTGCCAAAGGTTGTTCC |
| *CO* | F | AGGGACTCACTACAACGACAA |
|  | R | CGTTCTTGGGTGTGAAGCTG |
| *SOC1* | F | CGAGCAAGAAAGACTCAAGTGT |
|  | R | CGCTTTCATGAGATCCCCAC |
| *UBQ10 (reference)* | F | GGCCTTGTATAATCCCTGATGAATAAG |
|  | R | AAAGAGATAACAGGAACGGAAACATAG |
| *UBC21 (reference)* | F | CTGCGACTCAGGGAATCTTCTAA |
|  | R | TTGTGCCATTGAATTGAACCC |

ChIP-qPCR

| Gene | Name | Name | Reference |
| --- | --- | --- | --- |
| *FLC* | FLC-1F | ACTGGTTTGAACTCTTCCGACT |  |
|  | FLC-1R | ACTATGTAGGCACGACTTTGGT |  |
|  | FLC-2F | ATTTAGCAACGAAAGTGAAAACTAAGG | Deal et al., 2007 |
|  | FLC-2R | GCCACGTGTACCGCATGAC | Deal et al., 2007 |
|  | FLC-3F | AGAAATCAAGCGAATTGAGAACAA | Deal et al., 2007 |
|  | FLC-3R | CGTTGCGACGTTTGGAGAA | Deal et al., 2007 |
|  | FLC-4F | AAGCCAGCGCTATCACTAAACTTT | Deal et al., 2007 |
|  | FLC-4R | TCGGCAGATTGAAAATGACATT | Deal et al., 2007 |
|  | FLC-5F | CATCATGTGGGAGCAGAAGCT | Deal et al., 2007 |
|  | FLC-5R | CGGAAGATTGTCGGAGATTTG | Deal et al., 2007 |
| *FT* | FT-1F | TCGGACATTGGTAGGTATGGAC |  |
|  | FT-1R | GCCGCTTGTGTTATATGGTCG |  |
|  | FT-2F | AGTGTATTAGTGTGGTGGGTTTGG | Xu et al., 2014 (modified) |
|  | FT-2R | GTCGGTGAAATCATAACCACAATC | Xu et al., 2014 |
|  | FT-3F | GAGACCCTCTTATAGTAAGCAGAGTTG | Zacharaki at al., 2012 |
|  | FT-3R | GGGAGTTCAAGTGAAAGAACCAAAGT | Zacharaki at al., 2012 |
|  | FT-4F | CCAGATGTTCCAAGTCCTAGCAACC | Zacharaki at al., 2012 |
|  | FT-4R | GGTGTGGGCTTTTTTGGGAGAC | Zacharaki at al., 2012 |
|  | FT-5F | AATCCAAGTCCCACTGCAGG |  |
|  | FT-5R | GCGAGTGTTGAAGTTCTGGC |  |
| *CO* | CO-F | GCCCTGTTGTTCTCTCCACT |  |
|  | CO-R | GGATTCGATCTCCCCTCTACTT |  |
| *SOC1* | SOC1-F | CAAACCCTTTTAGCCAATCG | López-González et al., 2014 |
|  | SOC1-R | AAGGATGCAACCTCCTTTCA | López-González et al., 2014 |
