# Supplementary material for: AtEAF1 is a potential platform protein for Arabidopsis NuA4 acetyltransferase complex
Source: BMC Plant Biol. 2015 Mar 5;15:75. doi: 10.1186/s12870-015-0461-1 (PMC4358907; doi:10.1186/s12870-015-0461-1)
Supplement: Additional file 3: — DNA and protein sequences, alignments. [file 12870_2015_461_MOESM3_ESM.doc]

[1. CDS clones 1](#__RefHeading___Toc384913308)

[2. T-DNA insertion mapping 3](#__RefHeading___Toc384913309)

[3. Protein sequences 4](#__RefHeading___Toc384913310)

[3.1. HSA-ATPase proteins 4](#__RefHeading___Toc384913311)

[3.2. HSA-ATPase-SANT proteins 7](#__RefHeading___Toc384913312)

[3.3. HSA-SANT proteins 12](#__RefHeading___Toc384913313)

[4. Alignment used in Figure 1 15](#__RefHeading___Toc384913314)

1. CDS clones

>AtEAF1B FL CDS

ATGCATGGAAGCGTTTCAGGATATCTTCTAGTAAATGCTGAGGTTGATTCCATGGGAGGAGTTATCGATAGTGGAGGTGGTATTGGTGTTAAAACATCTCCGCGCCGAACAGCTATTGAGAAGGCTCAAGCGGAGCTAAGGCAAGAGTATGATGTTCGTGAGGAAAGGAGGAGAGAATTGGAGTTTCTAGAGAAAGGCGGTAATCCCTTGGATTTCAAGTTTGGTATTGCAACTTCACATAGCGTCCAATCTACATCACTCACAGATCAGCAAGCAGAGCATTTTGTAAACAGTGAAGTCAAAGATAGTTTTGCCCTGACTGCCTCACCACATGGTGACTCGGTGGAGAGTAGCGGTAGACCTGGAGTTCCTACAATTTCTGAACCCAATACAGCAGATAATCTTTTACTGTTTGATTCTGAAAACAAGTCAGTTGAAGGGGAAAGAAATTTGAGACATCCTAATAGGCAAAACAGAACTTCTGAGTCAGAACGATCTTCCAAAGCACACACCAACCAGAATACCAAAGAAACAGAAGATTCTGCCATCTTTCGCCCGTATGCTCGGAGGAACAGATCGAAGATAAGTCGGGATCCAGCACGGTCAAGCTCTACGGATTTAGTTCAGAATCGTGGTGGTCTTGCAACGTCTATATCTATTCGCAGAGGATCAGTGGAAGGAAAGGGTTGCATTCCCGAAGCAGCCAATCAAAAGGATATGCATACAACTTCTGTATCTTGTCCAGTATTTGCAAATTCAAATGGTAATATCGTTCCGAAAAATAGAGTTTCCAGCAATTCGCTGAATACTAAGGTGGATGGTGAACCTGTTGTACGAGAGAGTACTGCTGGATCCAAGACTAGTCTATTGAAAGATGAAGCCGACATTTCGTATAGTAAAAGCTCTGCATATTTGCCAGTTGGAGAGTCTGGCCTTGCTGGGGAGAAGGCACAACTAGTTTCGACTGGTGGTTCTCCCAAAGCTGCAACAATAGCTGGCCAGAAAAATAGTTCTACCCAACTGAATGGCCTAAGAGATTCCACGGTAGAGGAAGAAAGCTTAACAAATAGAGGAGCTACAGGGACTAATGGGTTAGAGTCAGAGTCTTCTCATGCGAACAACGTAGAAGTAAATGTAGATAATGAAAGAGATCTTTATAAAGTGGACAAACTTGACTCTGATGAAATCTCTATGCAGAAGACTTTGAGAGTAGAGGGGTTACTAGATCAAACAGTTGGTGAAATGACCAAAACTAAGATTGAAGATGAGACAGGTCAATCTACCACCATTATCAGTGAGTGCATTCCTGAATGTGAAATGCAGATGAAATCGGTTAAAATTGAAAATCAAAGTCACAGAAGTACAGCTGAGATGCAAACTAAGGAGAAAAGTTCCGAGACTGAGAAAAGGCTTCAAGATGGGTTGGTTGTACTCGAAAATGACAGTAAAGTTGGTAGTATTTTATCTGAAAATCCTAGCAGCACGTTGTGCTCTGGAATTCCTCAGGCATCTGTAGACACAAGTTCTTGCACAGTTGGCAATAGTTTATTGTCAGGAACTGACATTGAAGCATTAAAACATCAGCCGAGCTCAGATGCAGTTATGTTAGATACTGTCAAGGAGGACGCTATTCTTGAGGAGGCCCGGATTATACAGGCAAAGAAAAAAAGAATTGCCGAATTATCTTGTGGTACTGCACCAGTGGAGGTCCGTGAGAAATCTCAATGGGATTTTGTCCTCGAAGAAATGGCATGGCTGGCAAATGATTTTGCGCAGGAGCGCCTTTGGAAAATGACTGCTGCCACACAAATTTGCCATCGAGTTGCTTTGACTTGTCAGTTGAGATTTGAGGAACGAAATCAGCACAGAAAGCTGAAAAAAATAGCTTCAGTCCTATCTTATGCTATTTTACAATTCTGGAGTTCTGTGGAGGCCGAGGTTCCTGGGGAGCTGGAGGAGACGAGCTTGGGAATTGTTAAGGAGACCTGCCAAGAATCTAATTGTTTGAATGGCATAAGATGTTTAGCTGCGGGTGTCAAGGAATATGCAAGTAGATTTTTGAAGTACAACAACTCTTCCATCTCCTATCATTCAGCTGCACTGTCAACACCTGACAATATGTGCGACCCAGAAATATTGGATATATCTATGGTTGATCAGCTTACAGAAGCAAGCCTCTTTTATTCAGTTCCATCTGGTGCAATGGAGGTGTACCTTAAGTCCATTGAGTCGCATCTTACACGTTGTGAGTCTGGAAGTAGCATGCAGGAGGAGGTTGATACATCAGCCTATGATACTGCTGGAGATATAGGATATAATGTTACTGCGTTCGATGAGGATGAAGGAGAAACAAGTACCTATTATCTTCCGGGAGCTTTCGAGTCTAGCAGATCATTTAATATAAGCCACAAAAAAAGGAAGAATCTGATGAAGTCTCATTCTGCCCGGTCATATGATCTTGGGGACGATTTACCATACGTCAACAACACAGGTGGCTCTAACTCATCAAGTTTGATAGTAAAAAGGCCTGACAGTAATATTAATGCCGGCTCAGTTCCGACAAGGCGAGTGCGCACTGCTTCCAGGCATAGGGTTGTGAGTCCTTTTGGGTGTGCTACTACTGGGAATTTACCAGTGCCCTCGAAGACAGATGCGTCTAGTGGGGATACTAGTTCTTTCCAGGATGAGTATAGTAGTTTGCATGGTGGATCGGCAGTCCAAAAAGGCACAGAGGTTGAGTCAAGTGTTAATTTTGAAAAGCTGTTACCTTATGACATGGCTGAAACCTCAGGAAAACCTAAAAAGAAAAAGAAGACTCATCAGGGCTCCGCATATGACCAAACTTGGCATCTCAATCCGTCAGTCCATGTTGAACAGAAGGACCACTGGAAGAAGCGACCAGAGAATAATTTCGATATGAATGGTCTGTATGGTCCTCATAGTGCAAAGAAGCAAAAGACCACCAAGCAATTGGTAGAGAATAATTTTGATATGGCTATTCCTCACACTGGATCGATTCCTTCTCCGGCTGCTTCCCAGATGAGCAATATGTCCAACCCCAACAAATCTATCAAATTTATTGGAGGTCGTGATAGGGGCAGAAAAATAAAAGGCCTGAAGATTTCTCCTGGTCAGCATGGTTCTGGAAATCCATGGTCACTATTTGAGGATCAGGCGCTTGTTGTCCTGGTGCATGATATGGGGCCTAACTGGGAGCTCATTAGTGATGCAATGAACAGCACTCTTAAAATTAAGTATATATATCGCAATCCGACTGAGTGCAAGGACCGGCATAAGATTCTGATGGATAAAACTGCTGGTGATGGGGCTGATAGTGCAGAAGATTCAGGGAATTCTCAGTCTTATCCATCCACTTTGCCTGGCATACCAAAGGGAAGTGCGAGACAGTTGTTTCAACGACTGCAAGGGCCAATGGAGGAAGATACCCTGAAGTCTCATTTTGAGAAGATTTGTTTGATTGGGAAGAAATTACATTATAGAAAGACACAGAACGATGGTCGGGATCCCAAGCAAATAGTACCAGTTCACAATTCACAAGTCATGGCTCTCTCTCAAGTATTCCCGAATAATCTGAATGGAGGTGTTCTTACGCCCCTTGATGTCTGTGACGCATCAACTTCAGGTCAAGATGTATTTTCACTTGAAAATCCAGGTCTTCCAATGTTGAATCAGGGAACGCCAGTGCTCCCTACTTCTGGAGCACATCCATCCACTCCTGGATCATCTGGTGTTGTTCTGAGCAACAATTTGCCAACCACATCTGGCCTACAGAGTGCTTCTGTCAGGGATGGTAGATTCAATGTTCCTAGAGGGTCTTTGCCACTTGATGAACAACACCGACTACAACAATTTAATCAAACGTTATCTGGTAGAAACCTGCAGCAGCCCTCCTTATCAACTCCTGCAGCTGTCTCAGGATCTGATCGTGGACATCGCATGGTTCCTGGTGGAAATGCTATGGGTGTAAGTGGAATGAACAGGAACACACCCATGTCAAGGCCTGGTTTTCAAGGGATGGCCTCAGCAGCAATGCCAAATACTGGTAATATGCATACCTCTGGCATGGTAGGAATTCCAAACACTGGAAATATTCATTCCGGAGGAGGAGCTTCTCAAGGAAACTCCATGATCAGGCCTCGTGAAGCTGTGCAGCATATGATGCGGATGCAGGCTGCTCAAGGGAACAGTCCGGGGATCCCAGCTTTCAGTAATTTGAGTTCTGGATTTACCAACCAGACAACTCCTGTTCAGGCGTACCCAGGCCATCTTTCCCAGCAGCATCAGATGTCACCGCAGTCACATGTGCTTGGCAACTCGCATCATCCTCATCTCCAGAGCCCAAGTCAAGCCACTGGGGCACAGCAGGAAGCATTTGCTATCCGTCAAAGACAAATTCACCAGAGGTATTTGCAGCAACAGCAACAGCAGTTTCCAGCATCTGGTTCTATGATGCCACATGTACAACAACCTCAGGGCTCTTCTGTTTCTTCTTCTTCACAAAACAGTCCTCAGACTCAACCACCTGTTTCGCCCCAGCCATTATCCATGCCCCCGGTGTCGCCCTCTCCTAATATTAATGCTATGGCACAACAGAAACCTCAAAAATCTCAGTTGGCGCTTCATGGTTTGGGTAGGAGTCCTCAGTCTGGTACTTCTGGAGTGAACAATCAAGCTGGAAAACAAAGGCAGCGACAACTTCAGCAGTCTGCAAGACAGCATCCACACCAGCGGCAGCCAACACAAGGTCAACAGCTGAATAAACAACTGAAGGGGATGGGTAGAGGCAACATGATCCATCAGAACATCACTGTCGATCAGTCACACCTGAATGGCCTTACCATGCCCCAAGGAAATCAAGCTACCGAAAAAGGAGAGATAGCGGTTTCAGTTAGGCCTGATCAGCAGTCTAGTGTGGGGACTACCACGAGCACGGATCTGCAGTCTAAACCATTTGTTTCTCCCTTGTCTTCAAATCATTCACAGCAACTGCCAAAATCATTCCCTGGTGCTTTGTCTCCATCCCCTCAACAACAGATGCAATTACATTCAGACAATAGCATCCAAGGCCAGAGCTCACCTGCGACCCCATGTAATATTTTATCCACCTCTAGCCTGTCAATTGCACCAGCGGTTGCACCTTCTAACCATCAGCATTTGTTGATACACCAAAAGCAGCGCAATCAAGTGCAATCAACAGCTCAGAGAGTTGTTCAACATAATCATCTAGGGAACTCTGAGTTATCAAAGAAGTCCCAAGCTGAATGTATGCCACGTGTTCCACAGTCTGTAACCAATACCACCCAAACTGCTAGTATGGGTACGACCAAGGGTATGCCTCAAGCGAGTAATGATTTGAAAAACATAAAAGCAGTTGGTTCTACTGCGGTGCCTGCTTTGGAACCTCCATCTTGTGTTGCTTCTGTGCAAAGCACTGCTTCGAAAGTAGTAAACAATTCCAATACAGATTCAGCAGGAAATGATCCAGTATCAACACCGAACCAAGGACTAGCTCAAAAGCACGGGATCAAGGGCGTTACACAGAGACAACAACAGTCTCTACCTTCAGAAGAAAAGAGACCGAAATTGCCAGAGAAGCCGACTGTACAAAATCAGAAACACCTTGCTTCTGAGGAGCAGCCTCATTTAGAAGAAGCACAAGAATTATCATCTTCCAAGCCTCCTGATACAAAAGTGGAATGA

>AtYAF9B short splicing variant

ATGGAGTCGGATATCGAGATTTTGTCTGAAGCTGATGCGTCTATGCGGAAGCTACGCATTTTCGGAATTGATGATCGCGAAGATGAGAATGGGAGAAGAAGAATCAAAGATGTTGAAGTTTATGTTCCGATTGTGTGTGGATCGATTGCTTTTTATCTTGGAAAGAAAGCCACAGAATATCGAACACATAAGTGGACTGTTTATGTACGTGGAGCTACGAATGAGGATCTTGGTGTGGTTATCAAGCGGGTTATCTTCCATTTGCATCCAAGTTTTAATAATCCAACTAGAGTGGTTGATGCTCCTCCCTTTGCATTGTCTGAGTGTGGTTGGGGAGAATTCAAAATCGACATAACCGTTTTCTTCCATACCGATGTCTGTGAAAAGAAGTTGGAGTTGTCTCACGTCTTAAAGCTGAACCCGGAGAATGCATATGGTCCTATTCCTAAGTCTATTAAGATACCCGTTGTCGCTGAGTCTTACAATGAAGTTGTCTTTCCAGACCCTTTTGAGAGTTTTGTCGCCCGTGTTCATAATCACCCGGCTATACAGATCTCTAACATCCCAGATGGTTTGAACCTGCCTCCTCCAGGTGCATTCTTGTTC

>AtYAF9B long splicing variant

ATGGAGTCGGATATCGAGATTTTGTCTGAAGCTGATGCGTCTATGCGGAAGCTACGCATTTTCGGAATTGATGATCGCGAAGATGAGAATGGGAGAAGAAGAATCAAAGATGTTGAAGTTTATGTTCCGATTGTGTGTGGATCGATTGCTTTTTATCTTGGAAAGAAAGCCACAGAATATCGAACACATAAGTGGACTGTTTATGTACGTGGAGCTACGAATGAGGATCTTGGTGTGGTTATCAAGCGGGTTATCTTCCATTTGCATCCAAGTTTTAATAATCCAACTAGAGTGGTTGATGCTCCTCCCTTTGCATTGTCTGAGTGTGGTTGGGGAGAATTCAAAATCGACATAACCGTTTTCTTCCATACCGATGTCTGTGAAAAGAAGTTGGAGTTGTCTCACGTCTTAAAGCTGAACCCGGAGAATGCATATGGTCCTATTCCTAAGTCTATTAAGATACCCGTTGTCGCTGAGTCTTACAATGAAGTTGTCTTTCCAGACCCTTTTGAGAGTTTTGTCGCCCGTGTTCATAATCACCCGGCTATACAGATCTCTAACATCCCAGATGGTTTGAACCTGCCTCCTCCAGGAGTTGCTGACACTTATTATCTGATGGAAAAAGGAGACACTAAGGAACATCCACTCAGTCCATGGTTTTTGAAGTTTTCAGAAGTAGAAGAGCTTTTTAAACTTACTGCAGCTCGTCAGAAGGTACAAGCTGATATTGCCAAGCTAAAAAGACAGTTGATAATGGTAGATGGGCAACCTGAAGGACTTGAGTCTTCCTCTGGCTATGAATGT

>PIE1 HSA

atgGCGTCTAAAGGTGGTAAATCTAAACCTGATATAGTAATGGCGTCCAAGAGTGGGAAATCTAAACCTGATAATGAATCCAGAGCTAAGCGTCAGAAGACGCTCGAAGCTCCTAAAGAACCACGTCGTCCTAAAACTCACTGGGACCATGTATTGGAGGAGATGGCTTGGCTGTCAAAGGACTTTGAGTCTGAGAGGAAATGGAAGCTGGCGCAGGCGAAGAAGGTTGCTTTGAGAGCCAGCAAAGGAATGCTAGATCAGGCATCTAGGGAAGAAAGGAAGCTAAAGGAAGAAGAACAGCGACTTCGGAAAGTAGCCCTCAATATCTCGAAAGACATGAAAAAGTTTTGGATGAAAGTTGAGAAGCTGGTGCTTTACAAGCATCAGTTGGTACGCAATGAGAAGAAGAAGAAGGCTATGGACAAGCAACTTGAATTTCTGTTAGGCCAAACTGAGAGGTACTCAACCATGTTGGCAGAAAATTTAGTGGAGCCTTATAAACAGGGCCAAAATACTCCTTCAAAACCTCTACTAACTATCGAATCAAAAAGTGATGAGGAGAGGGCAGAACAGATACCTCCAGAGATAAATTCTTCTGCAGGT

>AtEAF1B HSA

atgTCAGATGCAGTTATGTTAGATACTGTCAAGGAGGACGCTATTCTTGAGGAGGCCCGGATTATACAGGCAAAGAAAAAAAGAATTGCCGAATTATCTTGTGGTACTGCACCAGTGGAGGTCCGTGAGAAATCTCAATGGGATTTTGTCCTCGAAGAAATGGCATGGCTGGCAAATGATTTTGCGCAGGAGCGCCTTTGGAAAATGACTGCTGCCACACAAATTTGCCATCGAGTTGCTTTGACTTGTCAGTTGAGATTTGAGGAACGAAATCAGCACAGAAAGCTGAAAAAAATAGCTTCAGTCCTATCTTATGCTATTTTACAATTCTGGAGTTCTGTGGAGGCCGAGGTTCCTGGGGAGCTGGAGGAGACGAGCTTGGGAATTGTTAAGGAGACCTGCCAAGAATCTAATTGTTTGAATGGCATAAGATGTTTAGCTGCGGGTGTCAAGGAATATGCAAGTAGATTTTTGAAGTACAACAACTCTTCCATCTCCTATCATTCAGCTGCACTGTCAACACCTGACAATATGTGCGACCCAGAAATATTGGATATATCTATGGTTGATCAGCTTACAGAAGCAAGCCTCTTTTATTCAGTT

>AtINO80 HSA

atgGTTTTACAGGTCCATTACGTGAAGATTCTGGAGAAGGGGGATACATACGAGATTGTTAAACGAAGTCTACCGAAGAAGCTGAAAGCAAAGAATGATCCTGCAGTCATTGAGAAAACAGAAAGGGATAAAATTAGAAAAGCCTGGATCAATATTGTCAGAAGAGATATAGCAAAACACCATAGAATTTTCACTACTTTTCATCGTAAACTATCAATTGATGCCAAGAGGTTTGCAGATGGTTGCCAAAGAGAGGTGAGAATGAAGGTGGGTAGATCATACAAAATCCCAAGAACTGCACCAATTCGCACTAGGAAGATATCCAGAGACATGCTGCTATTCTGGAAGCGATATGACAAGCAGATGGCAGAAGAGAGGAAAAAGCAAGAAAAGGAAGCTGCAGAGGCTTTTAAACGTGAACAGGAGCAGCGAGAGTCAAAAAGGCAGCAACAAAGGCTCAATTTCCTTATTAAACAGACTGAGCTTTACAGTCACTTCATGCAAAACAAGACCGATTCGAATCCTTCCGAAGCCTTACCAATAGGTGATGAAAATCCGATTGACGAAGTGCTCCCAGAAACTTCAGCGGCAGAACCTTCTGAG

1. T-DNA insertion mapping

Inserts start directly upstream of each sequence.

>SALK_067053 Ateaf1b-2

GAGATGAGGATGATGCGAGTTGCCAAGCACATGTGACTGCGGTGACATCTGATGCTGCTGGGAAAGATGGCCTGGGTACGCCTGAACAGGAGTTGTCTGGTTGGTAAATCCAGAACTCAAATTACTGAAAGCTGGGATCCCCGGACTGTTCCCTTGAGCAGCCTGCATCTGAAACTCATGAATATCAGTTTTTATCACCATTGTAGAGTTCGTAATGTTGTCAACGAGTTTCAAAATGACAATCGACCAGCGGTTGACAAAAATTAGAAGCGCTGAACGTCTACAAACTAAACAAAGAGCGAGATAAATAAGTCCAGGTAGTGAAGTAAGAAATAGATAGTTAAGTTCCCACATGTGAGAAATCG

>SALK_106430 Atyaf9a-1

TTTTTCCCGGTATCTTCGACTTGCACTAGTCATAAGATTTGTTGTGTGAGACGACATTTTAAGGCAATGATCAACTTAAACTAAGGAATGAAAAAAGCTTTAGATAAGGAGATCATACTAGGAGCAGGCAAGTTGTAACCAGAGGGTAATCTTGGAAAGGTCAAAGCCGGATGATTCTGAACCCTAGCTAAAAAACTTTCTGAAGGATCAGGAAAGACAATCTCGTCATAAGATTCCACAACCACAGGCTTCTTCATTGTCAAAGGACCTGATTCATCTTCAGGGTATAGCTTTAAATGATGATATCTGTTGTATCCACCCAAAACAACAAATAACTCATAAGTAACTCTTCCAATTGCTGGCATTTTCCTACTTAACAACAATGCTATTGAAAGGGACTTACAAACTCAATGGCTTATCACAGACATCACTGTGGAAATGCAATGTCATTGCAATCTCGAATTCTCCCCAACCAGATTCCGATACCTCGAATGGAGGCTCCTCGATAACTCTAGTCGGGCTATTGAAACTAGAATGAAGCTGAAATACAACTTTCTTTACTACAACACTGATATCTTCATTTGTTGCCCCTCGAACATATACTGCCCACTTGTGAGATTGAT

>SALK_046223 Atyaf9b-2

GGATGTTCCTTAGTGTCTCCTTTTTCCATCAGATAATAAGTGTCAGCAACTCCTAAAACATTGCAATGCGAGAATTATTATGCCTCCGGCAATAATTATTAATTCATTAGAAGTCAGAACAAGAATGCACCTGGAGGAGGCAGGTTCAAACCATCTGGGATGTTAGAGATCTGTATAGCCGGGTGATTATGAACACGGGCGACAAAACTCTCAAAAGGGTCTGGAAAGACAACTTCATTGTAAGACTCAGCGACAACGGGTATCTTAATAGACTTAGGAATAGGACCATATGCATTCTCCGGGTTCAGCTTTAAGACGTGAGACCTGGAAAGAAGTTCAATACTAAAACGTAAGAGATATAAACCAACTTACTCCACTGAAATTCACAATCACAAGGGAAACTCACAACTCCA

1. Protein sequences
   1. HSA-ATPase proteins

>NP_006653 helicase SRCAP [Homo sapiens]

MQSSPSPAHPQLPVLQTQMVSDGMTGSNPVSPASSSSPASSGAGGISPQHIAQDSSLDGPPGPPDGATVPLEGFSLSQAADLANKGPKWEKSHAEIAEQAKHEAEIETRIAELRKEGFWSLKRLPKVPEPPRPKGHWDYLCEEMQWLSADFAQERRWKRGVARKVVRMVIRHHEEQRQKEERARREEQAKLRRIASTMAKDVRQFWSNVEKVVQFKQQSRLEEKRKKALDLHLDFIVGQTEKYSDLLSQSLNQPLTSSKAGSSPCLGSSSAASSPPPPASRLDDEDGDFQPQEDEEEDDEETIEVEEQQEGNDAEAQRREIELLRREGELPLEELLRSLPPQLLEGPSSPSQTPSSHDSDTRDGPEEGAEEEPPQVLEIKPPPSAVTQRNKQPWHPDEDDEEFTANEEEAEDEEDTIAAEEQLEGEVDHAMELSELAREGELSMEELLQQYAGAYAPGSGSSEDEDEDEVDANSSDCEPEGPVEAEEPPQEDSSSQSDSVEDRSEDEEDEHSEEEETSGSSASEESESEESEDAQSQSQADEEEEDDDFGVEYLLARDEEQSEADAGSGPPTPGPTTLGPKKEITDIAAAAESLQPKGYTLATTQVKTPIPLLLRGQLREYQHIGLDWLVTMYEKKLNGILADEMGLGKTIQTISLLAHLACEKGNWGPHLIIVPTSVMLNWEMELKRWCPSFKILTYYGAQKERKLKRQGWTKPNAFHVCITSYKLVLQDHQAFRRKNWRYLILDEAQNIKNFKSQRWQSLLNFNSQRRLLLTGTPLQNSLMELWSLMHFLMPHVFQSHREFKEWFSNPLTGMIEGSQEYNEGLVKRLHKVLRPFLLRRVKVDVEKQMPKKYEHVIRCRLSKRQRCLYDDFMAQTTTKETLATGHFMSVINILMQLRKVCNHPNLFDPRPVTSPFITPGICFSTASLVLRATDVHPLQRIDMGRFDLIGLEGRVSRYEADTFLPRHRLSRRVLLEVATAPDPPPRPKPVKMKVNRMLQPVPKQEGRTVVVVNNPRAPLGPVPVRPPPGPELSAQPTPGPVPQVLPASLMVSASPAGPPLIPASRPPGPVLLPPLQPNSGSLPQVLPSPLGVLSGTSRPPTPTLSLKPTPPAPVRLSPAPPPGSSSLLKPLTVPPGYTFPPAAATTTSTTTATATTTAVPAPTPAPQRLILSPDMQARLPSGEVVSIGQLASLAQRPVANAGGSKPLTFQIQGNKLTLTGAQVRQLAVGQPRPLQRNVVHLVSAGGQHHLISQPAHVALIQAVAPTPGPTPVSVLPSSTPSTTPAPTGLSLPLAANQVPPTMVNNTGVVKIVVRQAPRDGLTPVPPLAPAPRPPSSGLPAVLNPRPTLTPGRLPTPTLGTARAPMPTPTLVRPLLKLVHSPSPEVSASAPGAAPLTISSPLHVPSSLPGPASSPMPIPNSSPLASPVSSTVSVPLSSSLPISVPTTLPAPASAPLTIPISAPLTVSASGPALLTSVTPPLAPVVPAAPGPPSLAPSGASPSASALTLGLATAPSLSSSQTPGHPLLLAPTSSHVPGLNSTVAPACSPVLVPASALASPFPSAPNPAPAQASLLAPASSASQALATPLAPMAAPQTAILAPSPAPPLAPLPVLAPSPGAAPVLASSQTPVPVMAPSSTPGTSLASASPVPAPTPVLAPSSTQTMLPAPVPSPLPSPASTQTLALAPALAPTLGGSSPSQTLSLGTGNPQGPFPTQTLSLTPASSLVPTPAQTLSLAPGPPLGPTQTLSLAPAPPLAPASPVGPAPAHTLTLAPASSSASLLAPASVQTLTLSPAPVPTLGPAAAQTLALAPASTQSPASQASSLVVSASGAAPLPVTMVSRLPVSKDEPDTLTLRSGPPSPPSTATSFGGPRPRRQPPPPPRSPFYLDSLEEKRKRQRSERLERIFQLSEAHGALAPVYGTEVLDFCTLPQPVASPIGPRSPGPSHPTFWTYTEAAHRAVLFPQQRLDQLSEIIERFIFVMPPVEAPPPSLHACHPPPWLAPRQAAFQEQLASELWPRARPLHRIVCNMRTQFPDLRLIQYDCGKLQTLAVLLRQLKAEGHRVLIFTQMTRMLDVLEQFLTYHGHLYLRLDGSTRVEQRQALMERFNADKRIFCFILSTRSGGVGVNLTGADTVVFYDSDWNPTMDAQAQDRCHRIGQTRDVHIYRLISERTVEENILKKANQKRMLGDMAIEGGNFTTAYFKQQTIRELFDMPLEEPSSSSVPSAPEEEEETVASKQTHILEQALCRAEDEEDIRAATQAKAEQVAELAEFNENDGFPAGEGEEAGRPGAEDEEMSRAEQEIAALVEQLTPIERYAMKFLEASLEEVSREELKQAEEQVEAARKDLDQAKEEVFRLPQEEEEGPGAGDESSCGTGGGTHRRSKKAKAPERPGTRVSERLRGARAETQGANHTPVISAHQTRSTTTPPRCSPARERVPRPAPRPRPTPASAPAAIPALVPVPVSAPVPISAPNPITILPVHILPSPPPPSQIPPCSSPACTPPPACTPPPAHTPPPAQTCLVTPSSPLLLGPPSVPISASVTNLPLGLRPEAELCAQALASPESLELASVASSETSSLSLVPPKDLLPVAVEILPVSEKNLSLTPSAPSLTLEAGSIPNGQEQEAPDSAEGTTLTVLPEGEELPLCVSESNGLELPPSAASDEPLQEPLEADRTSEELTEAKTPTSSPEKPQELVTAEVAAPSTSSSATSSPEGPSPARPPRRRTSADVEIRGQGTGRPGQPPGPKVLRKLPGRLVTVVEEKELVRRRRQQRGAASTLVPGVSETSASPGSPSVRSMSGPESSPPIGGPCEAAPSSSLPTPPQQPFIARRHIELGVTGGGSPENGDGALLAITPPAVKRRRGRPPKKNRSPADAGRGVDEAPSSTLKGKTNGADPVPGPETLIVADPVLEPQLIPGPQPLGPQPVHRPNPLLSPVEKRRRGRPPKARDLPIPGTISSAGDGNSESRTQPPPHPSPLTPLPPLLVCPTATVANTVTTVTISTSPPKRKRGRPPKNPPSPRPSQLPVLDRDSTSVLESCGLGRRRQPQGQGESEGSSSDEDGSRPLTRLARLRLEAEGMRGRKSGGSMVVAVIQDDLDLADSGPGGLELTPPVVSLTPKLRSTRLRPGSLVPPLETEKLPRKRAGAPVGGSPGLAKRGRLQPPSPLGPEGSVEESEAEASGEEEEGDGTPRRRPGPRRLVGTTNQGDQRILRSSAPPSLAGPAVSHRGRKAKT

>NP_010621 [Saccharomyces cerevisiae S288c]

MTTSRKSHAKDKKAGGEQDLADLKFRYDLLTNELFHLREFVSLVDYDPTHFNDSESFQKFLRETHLSLEERGEKFTDDVAKKGTNGDLTRRRRNLRTSTVVSSETTNEKKGDIELKLESIAPLVRNKCEELKYKLSDHSNRKSIVPQKRPIQHLKKREAAKSLKFKSERKENPLPLHEHIAEERYDHIAKVEEPSEAFTIKCPSDDSSFENTSEHYSDNFYFTTSSEEEDIKKKRGRKKKKPRIKLVVHPPKQTITNPLHVVKPGYESLHEYIASFKSLEDDLTLEEYNKYIDEQRRLLSRLKKGIENGALKYDKETDSLQPITSKEIKTIITYKPDPISYFYKQQDLQIHTDHLINQGIHMSKLFRSSTKARIARAKKVSQMIEQHFKHVAGAEERKAKEEERHKKSLARFAVQAVKKRWNMAEKAYRILRKDEEEQLKRIEGKQHLSKMLEKSTQLLEAQLNQVNDDGRSSTPSSDSNDVLSESDDDMDDELSTSSDEDEEVDADVGLENSPASTEATPTDESLNLIQLKEKYGHFNGSSTVYDSRNKDEKFPTLDKHESSSSESSVMTGEESSIYSSSENESQNENDRESDDKTPSVGLSALFGKGEESDGDLDLDDSEDFTVNSSSVEGEELEKDQVDNSAATFERAGDFVHTQNENRDDIKDVEEDAETKVQEEQLSVVDVPVPSLLRGNLRTYQKQGLNWLASLYNNHTNGILADEMGLGKTIQTISLLAYLACEKENWGPHLIVVPTSVLLNWEMEFKRFAPGFKVLTYYGSPQQRKEKRKGWNKPDAFHVCIVSYQLVVQDQHSFKRKRWQYMVLDEAHNIKNFRSTRWQALLNFNTQRRLLLTGTPLQNNLAELWSLLYFLMPQTVIDGKKVSGFADLDAFQQWFGRPVDKIIETGQNFGQDKETKKTVAKLHQVLRPYLLRRLKADVEKQMPAKYEHIVYCKLSKRQRFLYDDFMSRAQTKATLASGNFMSIVNCLMQLRKVCNHPNLFEVRPILTSFVLEHCVASDYKDVERTLLKLFKKNNQVNRVDLDFLNLVFTLNDKDLTSYHAEEISKLTCVKNFVEEVNKLRETNKQLQEEFGEASFLNFQDANQYFKYSNKQKLEGTVDMLNFLKMVNKLRCDRRPIFGKNLIDLLTKDRRVKYDKSSIIDNELIKPLQTRVLDNRKIIDTFAVLTPSAVSLDMRKLALGLNDDSSVGENTRLKVMQNCFEVSNPLHQLQTKLTIAFPDKSLLQYDCGKLQKLAILLQQLKDNGHRALIFTQMTKVLDVLEQFLNYHGYLYMRLDGATKIEDRQILTERFNTDSRITVFILSSRSGGLGINLTGADTVIFYDSDWNPAMDKQCQDRCHRIGQTRDVHIYRFVSEHTIESNILKKANQKRQLDNVVIQEGDFTTDYFSKLSVRDLLGSELPENASGGDKPLIADADVAAKDPRQLERLLAQAEDEDDVKAANLAMREVEIDNDDFDESTEKKAANEEEENHAELDEYEGTAHVDEYMIRFIANGYYY

>XP_761632 hypothetical protein UM05485.1 [Ustilago maydis 521]

MSPGDSALDYAKRQAGNVSSQAPSRPSSENDANGTVSTSNEHPSNEAGPSRPRSMGSERSASPQQGTTPKVPTKRRRLNAELAFSSPGPAFQVEQHGSEAQSDSNDSSGRARRPRRSTTLTKSGSNESQDRRSSAHIPKRKEEAGESRNLAESVPKNKLKLNNGKARASDEVELRESSLSLVLPSRSRSRSKSVVKLEPDQDSAFPQEVATPTLQPTRPKPQITPLTALNPQAALSEILARRRSERIALAQSDLEDVHDGHDMLVRELFHLTKFVTMVGYDPDVARTDQSDVFTTFKHAHDLRFSLDDSGSGAEASTAARVTRRRVNARLESLSLKRPDPPSTPSTPSFSKVKVDDDKKSAPEGRRNRRFSSVTGAQPRVNGAHTLETGHSDDTDDSSEEEDSEGSDLDAYSPDGREKGDAKDYNASRKGDAKRARLSSTPHKRHRAKVQDASSNAPVKRTGPRKSKALDELDAFILRQQPRPLPDHPPPLHILAPHQIPAHRRFGGDLDALYESFNMLQDDEGGLEDDDLETYIKLDQRWRAGLPIHPEAGSTTRHAVQKVPRNKSHHDHLLESVTSSYSQMRQYAKLKQQNSRKVARMIAQHWERQLGTSEREKKAEERRLRALAKWTLREVLKQWRLAVNVVRARKAAAEKAEKEKSDKEQLNAILEQSTAMLKKQHEVMTRADSLDDSDDEGSDRTNYGSDGSAESEISDIDDDDNQLIQIQDELPSVSPEQSLDMLTPVPEEADGSKDRVSNTTDHEAATANGDPTVVVESESQPSRRPQRRTARTKTFKARDSKLDADDIEFNDAGNDDEQEDAELERQMLEEDEEDDSEDAGLAADANIPIEELLKRYGYGQEADQDAEDSDAGEDAVSNDDSLENSATKDGSEDVAAVASIKIQEDAEVEEERPVQEDSMPDEAMDLEDDAVSTALNRPSDALLVDDHSDAESAATSGRRSSRRSMTRASSIVSSDRHATRLRQPFLLRGQLRPYQQIGFEWLCSLYANGVNGILADEMGLGKTIQTISLLAHLACDKGVWGPHLVVAPTSVMLNWEVEFKKFLPGFKILSYYGNQKERKEKRIGWNTENSFNVCITSYQLVLADQHIFRRKPWVYLVLDEAHHIKNFRSQRWQTLLGFNSQRRLLLTGTPLQNNLMDLWSLMYFLMPNGATELPGGGAFANMKDFQDWFSNPLDKAIEGGTSMNDETRAMVQKLHAVLRPYLLRRLKSEVEKELPSKYEHVITCRLSKRQRFLYNDFMSRAKTRESLASGNYLSIINCLMQLRKVCNHPDLFEVRPIVTSFAMSRSVVADYEIKDLLVRRRLLQENVWEKVDLDVTNLRITDGEEHLTAIESRDLRRLNAAKKLPHFREAVPEPRELDTWTLEGFERSREQRKLVDRMEKWKHMAYLNQYRCTKRPIYGSGLIKMLTEAGEAARLEPLEQHESDRRGFLTRCDSVLRIVQSRSTRRENMQALIDRFAFVTPRAVAVDMPRWALPGLEAHQRPDMVKREFDTVHPVAVKLHIAFPDASLLQYDCGKLQQLDILMRRLKEGGHRILIFTQMTRVLDILESFLNYHGYRYLRLDGATKVESRQALTEQFNRDARISAFILSTRSGGLGINLTGADTVLFYDLDWNAAIEAQCMDRAHRIGQTRDVHIYRFVTEHTIEENMLRKANQKRLLDNVVIQQGEFNTETLAKRLDWTDMLDESGKIGDVEVVVADQGVGARDVESAFLQAEDDEDRQAALRARHEMFIDDADFEEHQPSTSRPNTASATPLAHTASDGARPDNGAHAADALDAHEIENEHQEQEQEQEQAASIDDYMLAFVESDWPFFA

>XP_002504538 SNF2 super family [Micromonas sp. RCC299]

METLQEELDRAPLDPDAVRAEVSALEKESEIPVHMLLKRYGAPKEEVEAASVKAESEAGGDDEDEDTADDSEGSFNEDDDSDDDEDDDDEDDEDDEDDDDDDAMPPPGSQPDSRRVSLTINGVERPPDKVGALPVVFETPEEAKAAAIEEGKRIRYEDGVMDRAWDIMARPPLMKPVPPFPEPARNKTHWDHLLEEMKWLSGDFVRERKFRAKLARKAVYAVARSNLDLESRVIKREQDLLAAQRKTARNIGNEIMHFWIKIEKVVRFKAQAKVDSKRKQVMDKHLDFLLGQTERYSTMLAGKLTGEEEGALEGGEMKALPAPAQKSKPNPMPSVPEVKEEEDEDGDDFVAPDEDDMDDEETLEEEMRRAQAEGDDEDNDKEMADLAADAEVPIEELLRRYREMEEAEEKEKASKVSKKSVEIEDHEPTPMDTTCSPGEETRETTFADIVGEAGAVTGTSKRGQRGGKPAPAAAEALDDNDDFKIGDGEEEEDDEETLEEEMRRAQAEGDDDDHEKEMNDLAADAEIPIEELMRRYREMEAAHGGGEDLEEAEEEEGDDEEEDEEEEVEEEEEEDEPGVDALGGDDEPPMTEEQKAASRERRRVLDSLAGDAGSLQPKGHTLESADVKCRVPFLLKHSLREYQHVGLNWLVSCYDKALNGILADEMGLGKTIQTISLLAYLACNHGIWGPHLIVVPTSVMLNWEVEFKKWCPAFKLLTYFGTAKERKLKRQGWSKPNSFHVCITTYRLITQDQKVFRRKKWKYLILDEAHMIKNWRSQRWQTLLNFNSKRRLLITGTPLQNDLMELWSLMHFLMPHVFQSHSEFKNWFSSPLSGMVEGGEGINMDLVSRLHGVLRPFLLRRLKSEVEKNLPGKTEHVVHCGLSKRQRRLYEEYMASSDTSTTLSSGNLLGIINCLMQLRKVCNHPDLFAGRPIVSAFDMLPGVSIAVPSVVQNAARTLHEDPFRSKWFAPRGLHLLTIEDVNADAGGYGYASGDGWGCREALRRMAPVHEVEAALAQVTPKPSQRLGRMTQNADAAVRLFAEARAAAAKVERREVARRLALAAADAASRIPTYGTDLRRACVVRHPVHHCHLIERTFGVGPFATAPALLSAVKTYEARASDGADLVTAFMFAIPKARAPTPTMTCSAPSASVRAASRRIDAWAQKVGAPALAPLRLAQVRQQLFFPDRRLVQFDCGKLQALATLLRMKKSGGHKVLIFTQMTKMLDILEAFLNLYGYPYCRLDGTTRPEQRQIMMQRFNTDPRLFAFILSTRSGGFGINLTGADTVVFYDSDWNPAMDQQAQDRAHRIGQTREVHIYRLVCKGTIEENILRKSMQKRELDHFAIQAGNFNTEQFKKIAEAKARGETAPDGSGPGDEDEDKKRRADVVGGDAGFAAMNIFDKAMGAMGKSKPDNNKNGGGGGGDGTNEDDEVARLMDEAQDDADKAAAAAEAAGDADEAAEFGDDIREKDNPDTEDDDKSGGTSDQKSNKLKRVASASDGRSLAKASANKDEVTPATPTQKVGDADNGVGGDLSMVAINTSLEGDDQFAQDMMRKVQMSASKGEAIEQQLRPVERYAVRYLEETVRILDDVGIDADAVVDIEEKAWELDQLEKQKAAAEREVDDDEEGLVVEGWETGAADEEYRKKVEQAQEEARLQAEWERLEAERWAAMYAVPSAEDRSAAAAAAAATGAGGGGGLAGAIIGAKVPKGKRSSRDNTPGGTPRAGSPAPEGAAPALKVKFKLGPSLLGGGVKRECEESTDPELAHAHKKHKHKHKDETPEERAERKRMRKLTKEGAIAGGPQAQTPVKPNLTTAAAGAAVLQQSPTMTNPMEVSPGAGNNPMGYPAGMIPADFTIKEDAVISTLVLTMGDRAFSLGSELLSNNGPFIRPPAACKDRFRKLIALHGSSLAADVGSTWMASGQLRVTPELARALASKVANDAFPGDASSASPTGDGGPRRAPGPIRALLGAVQAMKASGDDATGVGKQGVVESVRNIFKVV

>XP_627205 Swr1p like SWI/SNF2 family ATpase with a HSA domain at the N-terminus probably involved in chromatin remodelling [Cryptosporidium parvum Iowa II]

MSKIRFQKSQLSIEPEPPKEIDEDHRDFLIKESKWMYGCFIDEHKWKIKSFKAVAQCAIRYLQTKNQRLKKRKEEEDKRLRIVSKNISVNINKFWNNISKIVRHRKLSELNKLLRIKQFEKLDKLVSETEKFYFGTNEDSGKKTQPNDKYCKIDLKSKSDSEDDLETDNWDYIEKVDNKLDLEMESSDFDSDEVNAELDELNNDASLNLEELYLKYYGNPYDKKNNLKRDKSDHAYIGDAIKRKKNDYCEEYVETTISKHIEEELGTEKEMNDTDKKNDFSLKDEFETLSNVANTPIDKAIANLEEKNNPQNCLETNNALAKVSIDQIKIPFLLKNNMREYQVAGLEWMVKLYKKGLNGILADEMGLGKTIQTISLLAYLACYMKNWGPHLIVVPTSVMLNWEMEFKRWLPCFKVITYFGTPKERQKKRIGWNDPNAFNVCIASYTLILQDAHIFKRKQWQYLILDEAQNIKNFKSQKWQVMLSFNTERRLLLTGTPLQNNLMELWSLLHFLMPHIFTSHHDFKTWFSDPLTTAIENQQVENERNLLSRLHSVLRPFLLRRLKKDVEKEMPSKIEHVIKCPLSKRQKELYDEFLESKTTQNTIAGGDYIGLMNVLMQLRKVCNHPDLFEPRTIKTPIVEKKLMINYSFSSLIFFPNICITMSSNVPGLGKNISTNNYLYIGKKTNKSKYTTLFDKLTNHRFVNIPNIFILYNEIHMSKFQAQSQCELTYKKWTGMQSCNYNIENSLFSKRTIGTENLLEINRFIRIGLDRLASVEFNSPLIGMDSKSTEYTKDVNKYILDHISNNKVKNKSNSVINSTESVSLFYKEDNRKIASEWRPSCDYPHLLDESVQIFHSNKIKNTNCDSKPCIKFGSPFNVVYGKDCRNFIFDQIQNKLKMVGEKTFSFSTFPNLFSNRKKSYKNTAISYKSIDYNTLNEMNAKLINLEMFPSKLLFVKPNFRKQLPMLSQFNMILERKVISTNFSIPIEGKYSFVKNELFCNSYISLQVDNLINSSNAYLHNISFFKKCIVPPRRIIEDDCGKFQILSRLLHKLFNEGHRCIIFTQMSKMLDVLESFINYRGYNYLRLDGSTKVDDRQKLVNRFNRDQRIYLFISSTRSGGVGLNLTGADTVIFYDSDWNPAMDRQAMDRCHRIGQTRDVNIYRLVSEWTIEESIFKKQLQKRLLDDVVVDQGRFTSEFFSKNDIQKMIGSRNQNMLNSDNNSIYVTRVLHESSTADSNASANFNDNQKKEFEDVLAAVEDLDDINALKKSSREIATENDDFINEFEEKIKHKVESNRTLEKDNVGNFNQYDITTNITLNNLLKYCIEFFESVSVPLDIQNEVDLLEFQINNINSDSSIENSLSEYSQGELPYD

>XP_002898448 SNF2 family helicase/ATPase and F-box protein, putative [Phytophthora infestans T30-4]

MANARLKQARRRASSRTPNAAQPVVHPPAQTPAASSLAQGASTRQDEQIADAQSEQVALQSALLERKSVLEGEIQDLTLGKTMHKEPEPGCDPSGRHVARYHRDYLLQEMEWMAADFSQERKWRLRNAKMLSQALVSHLDRQEQRLARQKKSEEIARRRTAARVGRDVKKFWTKIDKIIAFKVKLQADELRQKHMQKHLVQLVEQTEKYATALAASFQEAEEADDEEKAMESEDSDADFEMVDEEEDDEMTIEAEESRSGPLSKRQSAVEVATLQEEAEMSIEELRARYAAVEEMDEAGDSSEDGEFELTEEEDDDETTIAAEEQRNGPVSRRQAAAEMAELQEENELSIEELRARYAEALQADGETVAEQDEIMEVENTDDVGDRDFVPTRRDEEEQADDETTMEEEERLEGGVSPSQKAEELRLLEEEGEMSIEQLRARYAAASDEEAGSSQDNESDDAVDKQQEHPATDETMAEEAETQTCDTTSTLARKNGYKRPYLLTSRLDLREYQEAGVNWLVSMCERRINGILADEMGLGKTIQTISLLAHLACAQGLWGPHLIVVPTSCLVNWEMEFKRWCPAFKVLTYFGSAKRRKELRQGWSKQNAFQVCITSYQLVVQDAHCFKRKKWYYLILDEAHNIKNWKSLRWQTLLTFSSQRRLLLTGTPLQNNLLELWALMHFLMPHVFASRKEFSYWFQNPLALMVENGSDPTQSGDNGVEGGKDLVTQLHGIIRPFVLRRLKKDVAKQLPGKFEHVINCQLSKRQRFLYEDFISRSSTRRAMFGRGKGRGANFMSMMNVLMQLRKVCNHPDLFEPRPIASPLDMASLLVHAPSRCGFLVDEIVNERPRVALWTEVNLPGLELQCRDKLSSTRRRELFFYDVSAPLPEDSVAIVPSAYGDKRDVVRRIMKLAAKRREYWEQKRESVAQLQKIHVGLYLDEPVFGDALIRACTMPTFISAAMEVHMHRARPFLDAREPTQALQGMVRDPEERLESLQSLVNKSVCYVPKARARPARVIYGGGGFAYDDNFVLSRRAQLEEMEEEHAHPVASRVLTPYYNSFKRTQLFFPDKALVQFDCGKLQQLAVLLRTLKRGGHRCLIFTQMSSMLNILEAFLNLHGHTYFRLDGATKVDKRQMLMERFNRDEKIFCFILSTRSGGLGINLTGADAVIFYDSDWNPAMDAQAQDRAHRIGQTRDVHIYRLVSEHTVEENILRKAQQKRHLDFLVMSEGQFTTDFFSKASLRELMMSTGEEPDDVESESEDEDAEEDLDDDKEVSFDTVESAMAQLEDEEDVVAMKGARAEYLQELNEFDDDAARVASAGDAMSSKPSTPSSVVSGSTAASERGDEEDDEFGNEEDTADETGRRASRRDLATPDGSEHESMDDDEDHKKTPQSSRKRPRRSSTDRRKTPKRVKLSESHDQHGNNGEKAREKARDAAEEQKLQAWKASVQSLQGFEDSLNPVDRYALHFREDVDPLYAYSPAQQAAALAGVDVNPTAPTLLEDIEQTEAEKREEEVRLIAEGELVVGQMDDNEEASAEQTAERYTELYRRERAHVLFESRKRLLTGAAWSLMKCVISGQPFYFNADTREATWECPPVWIRNEQLKSAHKRGYEGLPPPALHRVMSMLTPYPERYRAQMVCRSWHTAAQHQSLYFKVSASDFESGSPTSLAKVLANVATGDTVLFGAGVYQLDETLEINKRLRLLAAPDARVELQMHSCRAQLRWSARGGVICGFHLTRTSSIPDAAAREIKPSSDAKEGMSAVKESRRVLRKRNKKLANWQHLLSVVGDGQLRVQYCEFDGNGLGNACVCVWGRGEKKKKGKKRRRAGSSASSAQTSKPGTPVVGPSPASTPAATPRAVTAVTPTIPVKPSASAATPVTTPSVAAAVNTSQCAVPSTSQSVVTSASTTSAAQVAKPVSVPPVSTLASATTPAPVSTITRIVAPTAPRPVNIAPKAMTPMSTGGTVTPRVTTPGATTPRPATTTIPAADTLLVLQNCRIRGAGSSGVLLVRGSLVMTLTTVEGNAHSGVTVLGGQALIRRNKIQRNARFGLRLLYHAGNVIVEDNVVFGNACGNLDVDNSGRRFVVRLNDMDKGKKTYDKLPHSHGKLRLKTYHVLEKEVPRPVPKPAPTLTSATSEYWKRQLTGTGATTTTATPVRPVISANNMMAARLPMGFMRPVMFPQAPNALHVSHLPLAFASLGAKPTIPSVTLNRSTTSAQLPTTAANLQRTLSAPGAASRPVIIPGTSSVSSTTATTVATPSGMSVLKYQRKRRPKTQQVVVGGREIVLRDTCEKPVEKIVKPRRPKDPQTPVPTSATMQHMTSPSALQLKFAPGSTAAAVAAAMNAMMSNSVKLQAAAATSKTQVQTVAATTPAIPAVPTTTMSTVTPRPVVTTTPSLQTVNTPSAATQTKSPVAPAAPTASTPTAAPITCTKTGTPQSTVGSLMTAKAGNLSTTASKLAVSGPSTDSVNDSTKPGEKRTLESAGIATKEESSVGLDGQPDKKVSKI

- 1. HSA-ATPase-SANT proteins

>NP_056224 E1A-binding protein p400 [Homo sapiens]

MHHGTGPQNVQHQLQRSRACPGSEGEEQPAHPNPPPSPAAPFAPSASPSAPQSPSYQIQQLMNRSPATGQNVNITLQSVGPVVGGNQQITLAPLPLPSPTSPGFQFSAQPRRFEHGSPSYIQVTSPLSQQVQTQSPTQPSPGPGQALQNVRAGAPGPGLGLCSSSPTGGFVDASVLVRQISLSPSSGGHFVFQDGSGLTQIAQGAQVQLQHPGTPITVRERRPSQPHTQSGGTIHHLGPQSPAAAGGAGLQPLASPSHITTANLPPQISSIIQGQLVQQQQVLQGPPLPRPLGFERTPGVLLPGAGGAAGFGMTSPPPPTSPSRTAVPPGLSSLPLTSVGNTGMKKVPKKLEEIPPASPEMAQMRKQCLDYHYQEMQALKEVFKEYLIELFFLQHFQGNMMDFLAFKKKHYAPLQAYLRQNDLDIEEEEEEEEEEEEKSEVINDEQQALAGSLVAGAGSTVETDLFKRQQAMPSTGMAEQSKRPRLEVGHQGVVFQHPGADAGVPLQQLMPTAQGGMPPTPQAAQLAGQRQSQQQYDPSTGPPVQNAASLHTPLPQLPGRLPPAGVPTAALSSALQFAQQPQVVEAQTQLQIPVKTQQPNVPIPAPPSSQLPIPPSQPAQLALHVPTPGKVQVQASQLSSLPQMVASTRLPVDPAPPCPRPLPTSSTSSLAPVSGSGPGPSPARSSPVNRPSSATNKALSPVTSRTPGVVASAPTKPQSPAQNATSSQDSSQDTLTEQITLENQVHQRIAELRKAGLWSQRRLPKLQEAPRPKSHWDYLLEEMQWMATDFAQERRWKVAAAKKLVRTVVRHHEEKQLREERGKKEEQSRLRRIAASTAREIECFWSNIEQVVEIKLRVELEEKRKKALNLQKVSRRGKELRPKGFDALQESSLDSGMSGRKRKASISLTDDEVDDEEETIEEEEANEGVVDHQTELSNLAKEAELPLLDLMKLYEGAFLPSSQWPRPKPDGEDTSGEEDADDCPGDRESRKDLVLIDSLFIMDQFKAAERMNIGKPNAKDIADVTAVAEAILPKGSARVTTSVKFNAPSLLYGALRDYQKIGLDWLAKLYRKNLNGILADEAGLGKTVQIIAFFAHLACNEGNWGPHLVVVRSCNILKWELELKRWCPGLKILSYIGSHRELKAKRQEWAEPNSFHVCITSYTQFFRGLTAFTRVRWKCLVIDEMQRVKGMTERHWEAVFTLQSQQRLLLIDSPLHNTFLELWTMVHFLVPGISRPYLSSPLRAPSEESQDYYHKVVIRLHRVTQPFILRRTKRDVEKQLTKKYEHVLKCRLSNRQKALYEDVILQPGTQEALKSGHFVNVLSILVRLQRICNHPGLVEPRHPGSSYVAGPLEYPSASLILKALERDFWKEADLSMFDLIGLENKITRHEAELLSKKKIPRKLMEEISTSAAPAARPAAAKLKASRLFQPVQYGQKPEGRTVAFPSTHPPRTAAPTTASAAPQGPLRGRPPIATFSANPEAKAAAAPFQTSQASASAPRHQPASASSTAASPAHPAKLRAQTTAQASTPGQPPPQPQAPSHAAGQSALPQRLVLPSQAQARLPSGEVVKIAQLASITGPQSRVAQPETPVTLQFQGSKFTLSHSQLRQLTAGQPLQLQGSVLQIVSAPGQPYLRAPGPVVMQTVSQAGAVHGALGSKPPAGGPSPAPLTPQVGVPGRVAVNALAVGEPGTASKPASPIGGPTQEEKTRLLKERLDQIYLVNERRCSQAPVYGRDLLRICALPSHGRVQWRGSLDGRRGKEAGPAHSYTSSSESPSELMLTLCRCGESLQDVIDRVAFVIPPVVAAPPSLRVPRPPPLYSHRMRILRQGLREHAAPYFQQLRQTTAPRLLQFPELRLVQFDSGKLEALAILLQKLKSEGRRVLILSQMILMLDILEMFLNFHYLTYVRIDENASSEQRQELMRSFNRDRRIFCAILSTHSRTTGINLVEADTVVFYDNDLNPVMDAKAQEWCDRIGRCKDIHIYRLVSGNSIEEKLLKNGTKDLIREVAAQGNDYSMAFLTQRTIQELFEVYSPMDDAGFPVKAEEFVVLSQEPSVTETIAPKIARPFIEALKSIEYLEEDAQKSAQEGVLGPHTDALSSDSENMPCDEEPSQLEELADFMEQLTPIEKYALNYLELFHTSIEQEKERNSEDAVMTAVRAWEFWNLKTLQEREARLRLEQEEAELLTYTREDAYSMEYVYEDVDGQTEVMPLWTPPTPPQDDSDIYLDSVMCLMYEATPIPEAKLPPVYVRKERKRHKTDPSAAGRKKKQRHGEAVVPPRSLFDRATPGLLKIRREGKEQKKNILLKQQVPFAKPLPTFAKPTAEPGQDNPEWLISEDWALLQAVKQLLELPLNLTIVSPAHTPNWDLVSDVVNSCSRIYRSSKQCRNRYENVIIPREEGKSKNNRPLRTSQIYAQDENATHTQLYTSHFDLMKMTAGKRSPPIKPLLGMNPFQKNPKHASVLAESGINYDKPLPPIQVASLRAERIAKEKKALADQQKAQQPAVAQPPPPQPQPPPPPQQPPPPLPQPQAAGSQPPAGPPAVQPQPQPQPQTQPQPVQAPAKAQPAITTGGSAAVLAGTIKTSVTGTSMPTGAVSGNVIVNTIAGVPAATFQSINKRLASPVAPGALTTPGGSAPAQVVHTQPPPRAVGSPATATPDLVSMATTQGVRAVTSVTASAVVTTNLTPVQTPARSLVPQVSQATGVQLPGKTITPAHFQLLRQQQQQQQQQQQQQQQQQQQQQQQQQQQQQTTTTSQVQVPQIQGQAQSPAQIKAVGKLTPEHLIKMQKQKLQMPPQPPPPQAQSAPPQPTAQVQVQTSQPPQQQSPQLTTVTAPRPGALLTGTTVANLQVARLTRVPTSQLQAQGQMQTQAPQPAQVALAKPPVVSVPAAVVSSPGVTTLPMNVAGISVAIGQPQKAAGQTVVAQPVHMQQLLKLKQQAVQQQKAIQPQAAQGPAAVQQKITAQQITTPGAQQKVAYAAQPALKTQFLTTPISQAQKLAGAQQVQTQIQVAKLPQVVQQQTPVASIQQVASASQQASPQTVALTQATAAGQQVQMIPAVTATAQVVQQKLIQQQVVTTASAPLQTPGAPNPAQVPASSDSPSQQPKLQMRVPAVRLKTPTKPPCQ

>NP_524833 domino, isoform A [Drosophila melanogaster]

MNEGNSAGGGHEGLSPAPPAVPDRVTPHSTEISVAPANSTSTTVRAAGSVGAALPATRHHQHIATQVKGIASSSSKQQKQLASAQLPVPLSPLPQQQQQTAEATAAAAAPAHSNVSVSSSTIEASVLPPQAKRQRLDDNEDRTSAASIVGPAESSNIVSSLLPASVASSSEVGGLSSTALQDLNALKKRILQQKLQILRNLKERHLENVSEYFYLQNGGSMMDYPAWRKKTPTPQFISYSNANRIDQLIHEDKPSTSAAAAAAQNQKYTTQQTDSVESSLVSGIGTGATKGAPLDGNISNSTVKTNTQSQVPSKIGSFTESTPAATESNSSTTVPGTATSGAATSTSATSAEASGNVLAVEAEIKIPAVGATPVAISTKLPAAVVQLTQQGGTPLLPCNTSAGSTALRRPQGQNNASSGSAAASGGGGSLTPTPLYTGNGPAALGGSGGLTPGTPTSGSLLSPALGGGSGTPNSAAQEFSFKAKQEVYVMQRISELQREGLWTERRLPKLQEPSRPKAHWDYLLEEMVWLAADFAQERKWKKNAAKKCAKMVQKYFQDKATAAQRAEKAQELQLKRVASFIAREVKSFWSNVEKLVEYKHQTKIEEKRKQALDQHLSFIVDQTEKFSQQLVEGMNKSVADTPSLNSSRLTSPKRESDDDFRPESGSEDDEETIAKAEEDAADVKEEVTALAKESEMDFDDFLNDLPPGYLENRDKLMKEEQSSAIKTETPDDSDDSEFEAKEASDDDENTISKQEEAEQEIDHKKEIDELEADNDLSVEQLLAKYKSEQPPSPKRRKLAPRDPELDSDDDSTAVDSTEESEDAATEDEEDLSTVKTDTDMEEQDEQEDGLKSLMADADATSGAAGSGSTAGASGNKDDMLNDAAALAESLQPKGNTLSSTNVVTPVPFLLKHSLREYQHIGLDWLVTMNERKLNGILADEMGLGKTIQTIALLAHLACAKGNWGPHLIVVPSSVMLNWEMEFKKWCPGFKILTYYGSQKERKLKRVGWTKPNAFHVCITSYKLVVQDQQSFRRKKWKYLILDEAQNIKNFKSQRWQLLLNFSTERRLLLTGTPLQNDLMELWSLMHFLMPYVFSSHREFKEWFSNPMTGMIEGNMEYNETLITRLHKVIRPFLLRRLKKEVEKQMPKKYEHVITCRLSNRQRYLYEDFMSRAKTRETLQTGNLLSVINVLMQLRKVCNHPNMFEARPTISPFQMDGITFHTPRLVCDIMEYDPFTQINLETLNLLLLHLEQTMTAYVSHKSRLLAPPRKLIEDIDTAPLPAPRCPNGKYRFHIRVRSAELAQRIKLNAVKVGASPAMRLEGSKIMPMRNLLPSGRVLKRVSASINPVNMALKPVVINSVVTTTSSSTTASSPTGALSVLSNSKLLGARSQINAPTPAKVAKTMQDGKPFFYLTPATNSGAAGARLTLTSKTTASASTTTSRTTVTASTTSGQQLIRDPIVKDLATHVKSTVQKQSIANGKTEPEEETEAEDPYKVQELIQMRKEQRLAALKRMAMINRRRTDATPIYGEDCREAIQRCMQATRSLKRSTWQTRGYANCCTAMAHRNGWSLNHLLKSFEERCADLKPVFANFVIYVPSVCAPRIRRYVQNLSSTHWQHEQRIENIVDQALRPKLALLHPIISEMTTKFPDPRLIQYDCGKLQTMDRLLRQLKVNGHRVLIFTQMTKMLDVLEAFLNYHGHIYLRLDGSTRVEQRQILMERFNGDKRIFCFILSTRSGGVGINLTGADTVIFYDSDWNPTMDAQAQDRCHRIGQTRDVHIYRLVSERTIEVNILKKANQKRMLSDMAIEGGNFTTTYFKSSTIKDLFTMEQSEQDESSQEKSENKDRIVATTTLSDTPSTVVETEKQSLRAFEHALAAAEDEQDVQATKTAKAEVAADLAEFDENIPIATEDPNAEGGPQVELSKADLEMQNLVKQLSPIERYAMRFVEETGAAWTAEQLRAAEAELEAQKREWEANRLAAMHKEEELLKQETEAEEMLTYSRKDSSNQVWISRNTMEQMPMWCPPTPPQDNDNDIYIDYSLSFMYELEPIAETDLPPVYVRKEHKRSRTDAGYDGSRRPNKMRREDNYVPPRSLFDRPTPQLARLRRELKSQRFRGSFKPNMPIPGLKPQLPTKPLTEPEAMAEWCVFEDMAILHVLVNLQGLPCSLMLLSPGQTPNWDLVSEMVNFCSKTYRSARQCRWRYETHIQPREEGKVVESPKKQKKLKPTLRTEYLKSPLRYLRTTQLYVSDNNASFYKTMRSRFDSIKTAYLKKAPPPKRQFSAPSLMNPKHMEVLQEFGILNYDQPVSPQNIAAMKANKIREKQRGQQMSQPPVGVGVVQQMQQQSQQQQQPAPPPLPQQQQPQQVVQQVQQQQQQQQQQQQQQVVQQQLPTVSNVQQTLPVQQTVELVQQQPTTTTTVAVPAAGGQLQQLQIQHLTSSNVSPGQQTAILLHQPQQQLRTHPGQGGQSNTQQLVKTIVGTSSSLTAGQLQQLAQQSAVASGGQSSVSVVLTTPVQTLPSVVQPQIGSGAQIVSISSQTLPVNSSPQLGSIVQTQSLPQVVSVSTLPTVGTVLTTTANQPQQQHQTTAVTTLNTTMLRGQRIVSTAAGNTLQQRTTAGGQSIVSMPNLGQGASPSQFQTQLRLAAVPTSPATQTTQLVTTKGIPVSALQQGGKTTVIPVTQQSGGAHIQLYRQRSLKVLQTTTQAVPSGSAGATGATANLVQAGGTIIQASNMATHVTSQKVAVSGMPGTSTTVQAGNVVSSVQMHGQARTQFIKQMAAGKQQLQRQVVSADGTTTTTAAGDMLLVKRHNILAAQKAQQASGALFTTTTGQQQQQQQQQGQLPVAGQPQQVTQHQIASLVKASTAAAASGSSVNAGGVTVSATNPTVQAGSVNMTLPQLKPGSQIKVTMPNQMRHLQMQQQLTMPRKISRMTQLVSASGQPTATNIITTTGPQQQQQGVTVSGGGTLPTVASQQQQQQHQQKVGGGNSVQAQLLHIQNTKGLSNSVTMQQIQQVMRSGQQGTLATTNLVLGKTSVGRVIPVSVASQANQRQTIQVVSAASAQALAAGNLRTHVAGPSIASTLKVAAPGSAGGQTTQQTLIAALQHNQRQNASPVRLQTTAGGNLLAVVQQQQQQQHTSIAGPTAGPAEVMTITQTTTTLPTVGSLQQQQQQQQQQGGISQPTTQQVRKLVQKKILIRSEKE

>XP_004333644 SNF2 family Nterminal domain containing protein [Acanthamoeba castellanii str. Neff]

MDPSTSGGGKDITSPPSATAAASAEAHTQIRKKKAPGLASTNKRKRTAEVADSSAKGGIETNLKRPRTPEARASSKQQTVGKEEGADVNDDPAALVELLRTQALGTEGARRAQWLQRRERKLRVLSHLEHGESLLAFLLQAHHPAAAAAQPPPSTACDPPPPPLWPPTTAAHASPAAPPCSASSPPPPPRGGEVADSHLVLQGGHEAGARRVEEEEEEETMPPLRGLALPSTAAAAVAVTIGAAAAAGGGGGIRAEEAGAGAKGRAEWEAWVQGRVGALQREGLWSGRRAGKVPEPPRGKTHWDYVLEEMAWMAHDFAEERRFKMALARRVSKEVLRYHQARRTREERESRKEEQRLRRQATTIAREVVAFWHSVEKLVRHKHQTRLEQRKREALDKHLDFLVGQTERYSTMLAEELATPSITLPSATTAATTHRDGGADGLASSSSSSIGLPEAPLRLEPRMAYESASLPRAEESVGVDGDDGEYLLERDQAALQVQADDEATLEQEEAQQHEEGSINATTAQQEIDSLRAEADMPIEELLALYAARRGEEDEEEEEEQAEGEEKDAMDIEDAAATTAATTAATTAATTAATTAATTESLRVVATASGRTPGERDEAEGLTAELLAVGNHHHHHRRAEDNDDEDDEGRARIRRAAAEATEAQPTGNTLSTTHVKTPVPFLLKHTLREYQHIGLDWLVTMYEKGLNGILADEMGLGKTIQTISLLAHLASEKQNWGPHLVVVPTSVMLNWEREFRKWCPSFKLLTYYGNPKTRRLKRTGWSKPNAFHVCITSYTLVLQDHQVFRRKQWKYLILDEAHNIKNFKSQRWQILLNFNAQRRLLLTGTPLQNDLMELWSLMHFLMPHIFRSHSQFKKWFSNPMNDIIDSGGDRQQQQQQDALVARLHAVLRPFLLRRLKKDVEQQLPRKVEHIVPCRLSKRQRHLYEEFISRADTRDKLASGNYLSLVNVLMQLRKVCNHPELFEERPVVSSLDMGQSISMHVPSAVVNMLAYEAMDDVDLGFLNLRFVDNEPMTSWQSDRVGELRTTREQATAFCSSSSWTSSLWPRGEKQQQQQEEEESSAEWSRVRPLALGRWREDEQRRLAHLVYSNEWRCAQRPLYGLDLRQCVRVPARTPPPPHLAAAEDGHDAGPPPLVLSHEMRALAVRDLVTRFTCIITPARAPPPLLHCSHPRPSDTEAMARTAYHLTQNYFGRLWWGSKYTSPIVSPAAALDVLARSVGQFLTPGPEGAKELALPLATVRSVFHALLHLPAPEGQAPLRLAATSAVKDPERNVHLWQNFCLFESLYGASTARSAFEAALSALPTASDRLPIWHQYVALRQGIEGAAGVQKVVDLLSRCFSDVATPAAAPLPQQLQSLHPYARKVIRDTAPMSYAWHGLLLESALAGLHPTQRGPVQRAVLQIIPAYTLLSFSVARFELGRSNIPAARSLLDAALRLNPAFDVVWLNAARVRMREGDEAGARQLLQKAVQTHPTSALLWLNWIALARGGDEQAKETEAERRSQAERRGVDMMWCVLFYVAHTGLLQYDCGKLQELDRLLRTLKQGGHKCATGGAGSASVRSRRMLDVLEAFLNIHGHTYLRLDGATRVEERQHLMERFNADPRIFLFILSTRAGGLGVNLVGADTVIFYDSDWNPAMDQQAQDRCHRIGQTREVHIYRLVTQHTIEENILRKANQKRHLEQLVLTEGQFTTDFFQKVTISDLLQHGEGAASGAGGGDGGLEPLARKDSSVSWTPKDWERAIASVEDESDVHAMEQLKKELSEEMQEFTEDPHHPLTPGGPAAVHAGKAAAAAADNETFLSVLSPIERYAFKFLDEVNPVVDAQELEAMHEEVEQEERQWERDVLQRIKKEELLLAQEPPAPSAQDEELLFYEVVGSVSEWREAALSGSAVYGAWCPEEDQLYVPSYDRELFPELYPYPDLPSCIHYLPRHDHDHHDHHPDPASPPSLSCPPSAKKAAAAASASAPSSGASSSSLRQTTLTPFRQKHHPAPHKADRPPLSAASTPAKHTTPTLQKKKIMKMKMMKIRHAPPSSEPLLPELPDMEVPTEAPTKRPNKADTEAAAAATTTTTKKRKGKPAATGRSSAGPSSPPSPVLQQSAEIASASDKTSGSGQKQPAKKKRRTVKEDKGVGPAAKLKHAKGDKALTTAAAAAKKKKKPSKSPATPRHAKGPTKRGADKEADPSTTTGGAALALPEEERVLDRAVQAYGPSPNWDLIADIVNSAPPTRYHRRSRQQCLQHWAMLSASTSAPAPGTSAPPPGSASVAAATSTSSSSGVLPSSAHPGLGPAAARGGLARLGSASLALPHHHHHHQQQQQQQQHHHHQQQQQQPGTGLSLALGHASASTSSASALACGSPAAAVRAKAQHQPTPVPCGPVTPGLSHRATHLSPLSATAGGGGAGSGFTATIAAAAASSSSSIALLHRTAGRGGHVVAPSSSSSASLSSSSSPAALSSVTSAAASSWPLNLTPLKPPTLSAPHPAPPEAAAIATTQAAPQSSSTPLKGKLPDHHHHHQPPT

>NP_187887 photoperiod-independent early flowering 1 protein [Arabidopsis thaliana]

MASKGGKSKPDIVMASKSGKSKPDNESRAKRQKTLEAPKEPRRPKTHWDHVLEEMAWLSKDFESERKWKLAQAKKVALRASKGMLDQASREERKLKEEEQRLRKVALNISKDMKKFWMKVEKLVLYKHQLVRNEKKKKAMDKQLEFLLGQTERYSTMLAENLVEPYKQGQNTPSKPLLTIESKSDEERAEQIPPEINSSAGLESGSPELDEDYDLKSEDETEDDEDTIEEDEKHFTKRERQEELEALQNEVDLPVEELLRRYTSGRVSRETSPVKDENEDNLTSVSRVTSPVKDENQDNLASVGQDHGEDKNNLAASEETEGNPSVRRSNDSYGHLAISETHSHDLEPGMTTASVKSRKEDHTYDFNDEQEDVDFVLANGEEKDDEATLAVEEELAKADNEDHVEEIALLQKESEMPIEVLLARYKEDFGGKDISEDESESSFAVSEDSIVDSDENRQQADLDDDNVDLTECKLDPEPCSENVEGTFHEVAEDNDKDSSDKIADAAAAARSAQPTGFTYSTTKVRTKLPFLLKHSLREYQHIGLDWLVTMYEKKLNGILADEMGLGKTIMTIALLAHLACDKGIWGPHLIVVPTSVMLNWETEFLKWCPAFKILTYFGSAKERKLKRQGWMKLNSFHVCITTYRLVIQDSKMFKRKKWKYLILDEAHLIKNWKSQRWQTLLNFNSKRRILLTGTPLQNDLMELWSLMHFLMPHVFQSHQEFKDWFCNPIAGMVEGQEKINKEVIDRLHNVLRPFLLRRLKRDVEKQLPSKHEHVIFCRLSKRQRNLYEDFIASTETQATLTSGSFFGMISIIMQLRKVCNHPDLFEGRPIVSSFDMAGIDVQLSSTICSLLLESPFSKVDLEALGFLFTHLDFSMTSWEGDEIKAISTPSELIKQRVNLKDDLEAIPLSPKNRKNLQGTNIFEEIRKAVFEERIQESKDRAAAIAWWNSLRCQRKPTYSTSLRTLLTIKGPLDDLKANCSSYMYSSILADIVLSPIERFQKMIELVEAFTFAIPAARVPSPTCWCSKSDSPVFLSPSYKEKVTDLLSPLLSPIRPAIVRRQVYFPDRRLIQFDCGKLQELAMLLRKLKFGGHRALIFTQMTKMLDVLEAFINLYGYTYMRLDGSTPPEERQTLMQRFNTNPKIFLFILSTRSGGVGINLVGADTVIFYDSDWNPAMDQQAQDRCHRIGQTREVHIYRLISESTIEENILKKANQKRVLDNLVIQNGEYNTEFFKKLDPMELFSGHKALTTKDEKETSKHCGADIPLSNADVEAALKQAEDEADYMALKRVEQEEAVDNQEFTEEPVERPEDDELVNEDDIKADEPADQGLVAAGPAKEEMSLLHSDIRDERAVITTSSQEDDTDVLDDVKQMAAAAADAGQAISSFENQLRPIDRYAIRFLELWDPIIVEAAMENEAGFEEKEWELDHIEKYKEEMEAEIDDGEEPLVYEKWDADFATEAYRQQVEVLAQHQLMEDLENEAREREAAEVAEMVLTQNESAHVLKPKKKKKAKKAKYKSLKKGSLAAESKHVKSVVKIEDSTDDDNEEFGYVSSSDSDMVTPLSRMHMKGKKRDLIVDTDEEKTSKKKAKKHKKSLPNSDIKYKQTSALLDELEPSKPSDSMVVDNELKLTNRGKTVGKKFITSMPIKRVLMIKPEKLKKGNLWSRDCVPSPDSWLPQEDAILCAMVHEYGPNWNFVSGTLYGMTAGGAYRGRYRHPAYCCERYRELIQRHILSASDSAVNEKNLNTGSGKALLKVTEENIRTLLNVAAEQPDTEMLLQKHFSCLLSSIWRTSTRTGNDQMLSLNSPIFNRQFMGSVNHTQDLARKPWQGMKVTSLSRKLLESALQDSGPSQPDNTISRSRLQETQPINKLGLELTLEFPRGNDDSLNQFPPMISLSIDGSDSLNYVNEPPGEDVLKGSRVAAENRYRNAANACIEDSFGWASNTFPANDLKSRTGTKAQSLGKHKLSASDSAKSTKSKHRKLLAEQLEGAWVRPNDPNLKFDFTPGDREEEEEQEVDEKANSAEIEMISCSQWYDPFFTSGLDDCSLASDISEIE

>XP_003557609 PREDICTED: uncharacterized protein LOC100821638 [Brachypodium distachyon]

MASKGARSKLDHETRARRKKALEAPREPRKPKVHWDHVLGEMVWLAKEFESERKWKLSMAKKIAQRANMGIVDQATKDEKKQKEGEYRLRKVALNISKDVKKFWTKIEKLVLYKNQLEVEERKKKALDKQLDFLLGQTERYSTMLAENLVDVPHLQTQENGPLQTNLPSQEEEVAEENTNALMHDDLDKMEIDDDYNSSLNEEPEDDEHTIDEDEAQITEAERNEELAALQAEADLPLDDILKLYAKTKVSRESSPDSKDTFSKSDLKNLMKDPSNQANGCNHESGGTSSDEGNSSEEVDDSYSYTEFVKKNHGKSNGSISSVGEQGDKDYVAADEGKDDEATLSEEEELAKGDSPDPLDEIKLLQKESEIPLEELLARYQMDGYADGVTTELENSPTHYNEEVNTDMSLDGQSVDILKLNNDMLENHEITDMLERKLVSGNALQPEIVPESSVQGCSVKEDELTDAKVANEETGDSVIDDAAAAARSAQPTGNTFSTTSVRTKFPFLLKHSLREYQHIGLDWLVAMYEKRLNGILADEMGLGKTIMTISLLAHLACEKGIWGPHLIVVPTSVMLNWETEFLKWCPAFKILTYFGSAKERKQKRQGWMKPNFFHVCITTYRLVIQDSKAFKRKKWKYLILDEAHLIKNWKSQRWQTLLNFNSKRRILLTGTPLQNDLMELWSLMHFLMPHVFQSHQEFKDWFCNPISGMVEGQDKVNKEVIDRLHNVLRPFILRRLKRDVEKQLPQKHEHVIYCRLSRRQRNLYEDFIANSETQATLTSGNYFGMISIIMQLRKVCNHPDLFEGRPIISSFDMAGINMQISSSVCMVLDKGPFSQADLSDMNLVFTQNEFNMTSWEVDEVADAFSPGITSRGSGAEFSCSNKDGQRGIGKNIFEEIQKALQEERMKEAKERAASIAWWNRIRCQKRPVYGTNIRELLTIRHPICDVLEKKSNPLCHMEFSSSLADLVLSSVERFNKMLGFIESFTFAIPAARAATPICWCKKRKSPVLLEPAYREQCMNEFSPILSPIRPAIVRRQVYFPDRRLIQFDCGKLQELAILLRRLKSEGHRALIFTQMTKMLDTLEEFINLYGYTYLRLDGSTQPEERQTLMQRFNTNPKYFLFILSTRSGGVGVNLVGADTVIFYDSDWNPAMDQQAQDRCHRIGQTREVNIYRLISESTIEENILKKANQKRALDDLVIQRGSYNTEFFKKLDPMEFFSGHAPLNVEDQQKDRSMPSVVSNETGLALSNADVEAAIRQAEDEADYMALKRLEQEEAADNQEFSEEAAGRLEDDELVNEEAKPDEHCSAEHKHQCSDVDNDKSVALPVNQLDEEKALRLAAGDGDMDMLADVKQMAAAAAAAGQASSSFENQLRPIDRYAMRFLELWDPIIDKAAVNYQANVVEEEWELERIEKLKEDLEAEIDEDQEPLSYETWDVDFATTAYRQHVEALAKKQLLEEQEKQACKAAKELEETNDIISHRKKSKKNKRKAGKFKSLKRGRLSSESEAMLDETSVDTMSIDGNAPSPELISDESPHHCSHKRKKMVSRNEEVNSSSRSLKKFKKAPKSNCISESSSHKHLLEGKQLKLMDEVNFSDPKLVSIRSDGRISTPCMPVKRVMVIKPERLKRKGLIWPRDCVPDSWTNEEDAVLCGTVHEYGPVWELASEFLHSIPGGAFYRGRYRHPVHCCERFRELICKHVLSAMDNTNSEKVPSGTGKAILKVSEDQTQMLLNAISEIPNNELLLQKHFMAILSSVWRSKCGHEPRRVTSTCSSALNKPVRLNEKWSMTNYRPTSNLIKTALADAQAQCPRAVLPRNQESGRNYLELVLDFRTDQHAYEADFPSVVNVSILEPEPVKRAIVQVDQSLLSGLSHRNAEKRFRIASEACFDGEGSHWASSAFHVYDAARHKSGPKSVGKHKTSSESGRPAKSKIQRTTEPQDVPTAMNDFLRAPGQLLASAAEFHIAQSLSDFGINDSEFTCFHDLPLETDTEFAPCQYELASLPGIEELDPLSDFTDIG

>XP_002977590 hypothetical protein SELMODRAFT_450749 [Selaginella moellendorffii]

MEAGGAAAKPQLRCYGRRNRSKSAKEEVSSPGADDARSNGKLPGDKRLVEDSVGQEKAPGDSKLGLASEESKLNAGKGAVTPSKLEPLQSDAGKTQELCVVATEAKVLDETPQKDSVRFLNEEEELAYEDSVLSRAEEIWTRQEALEQVKRRAPSKWKEPTHAKSHWDYVLEEMVWLSKDFDKERKWKVAQAKRVAMKVAKAKSEEESKGMRRLKGEEQRIRRIASSIAKEVKKFWVKIEKLVVYKHQLAVEQKKKKALDKHLNFLLGQTERYSSMLAVNLSGLPENNEDQKTQDTGDLLDAANEPSTDKDFTCDQMEEEDDEATLEADEALITEDERKEELNALQRESELPIEELVGLYAKGDNSEEERSTSDADPDKELDDEEYDPHTAYASEEDDERTFDEEEKLAMAENKDVNLELEQLKMENELPVSELLSRYRAEGAESSGDEKGGASDAPSDSSDEAKERMGEPSTSGRSSLLIIKSTGRSKATGESKIFYGTEQEAEVLANVARAAQQRGERMRKRAGLDDSSRVEGSGKTGRKELDDAAAAALSAQPTGYTFSTTRVRTKIPFLLKHSLREYQHIGLDWLVTMYEKRLNGILADEMGLGKTIMTIALLAHLACEKGIWGPHLIVVPTSVMLNWETEFMKWCPAFKVLTYFGNAKERKIKRQGWSKANSFHVCITTYRLVIQDAKAFKRKKWKYLILDEAHLIKNWKSQRWQMLLNFNSKRRILLTGTPLQNDLMELWSLMHFLMPHVFQSHQEFRDWFSNPISGMVEGQDKVNQDVVDRLHNVLRPFILRRLKRDVEKQLPGKHEHVVPCRLSKRQRNLYEDFMASSDTQATLSGGNFLGLINVLMQLRKVCNHPDLFESRPIVSSFDMPGLQLQMCSELCTAVQQKPFSTVNLEVLNYLLSEDMEAWEPSELAELATPTPLIEEIATSGEDSWNEQEQVTEPQNIFEEIQAALAAQRLKRRREKARQFGWLNMLRCSRHVVYGRGLAKHVEVEHPVYGIHSIQSDPSRYLTFPSAIAEVVKLPSSWCESLLDLLQAFVFVIPAARAPPPLIWCSRQSASSILRHDFPSNQLAVMSDLVAPLRPVIVRQQLFFPDRRLLQFDCGKLQQLSVLLRRLKSEGHRALIFTQMTKMLDILESFINLYGYNYMRLDGSTKPEQRQILMQRFNTNPKIFLFILSTRSGGVGINLVGADTVIFYDSDWNPAMDQQAQDRCHRIGQTREVHIYRLVSESTIEENILKKANQKRFLDNLVIQSGGYNTEFFKKLDPMELLSGVNTTKRADQPLSNADVDAALKSAEDEADYMAMKKVELEEAAENQEFAEDVSVEEDECADDLEDGKVSETKVPSPEFETAGTPSLPELKEPQPLLLDAEEEMDMLADVKQLAAAAAASGQGNNFEDQLKPIERYAIQFLDLWNPIIDTSALETQVTYEEKEWELEQIEKLKEEQEADIDEDDEPLLYESWDTSNADAAYRQQVEVLTQQQELLQAQWDAMHDEELQEADRLIEGEKQQKRPKKKKLKSLERSSVEEYNFCSEEDAGADDHIDADEADEVLSIASDEGEYDMQWDNSLRTPFQRKRKFSRFFEEEGGENKRFRKEDSRTEYFRPRFGGKLTICTTARKSPVILLERDRRKEALKAKDHHTDWMPEEDEVLCAVVHEYGGNWLLASDALEGMPDGGVYRGRHRHPVKCKDRFRQLVVENAGTICGGVSSERLLSGAVIKVTEEDTKRLLELVQRVPDKEVLLQRHFATVQSVKDSYKGSAKSRNGFVSNVGHPRNLPFVSLANLCQGCLKPPLRQASHKQGHALVAEALSQTTASDNGKMPENSACKAVKNNTASDNNDNGDVESQPPLELSLSFSDPEEMEDFRPVNVTLRPGEPKTAEPPSSSSIRSLVCETRFRLASQMTPSIWAAAAISVVPKPTPSQNNPPVKRQQPPTDVAKLQRPGKQPRLQPVSKPNGMIEIRPVEMKTSVSPSQIKPANLKKPPPTAVFTTTGASRVHHVATKPSSSSSIQLARPSSSAKSTAVAAAAAGSTAAAAAAGGANYPFAQYWRPDSTVSSSRLAQAPASEEASYHRKPAAAAKNKGVSE

>XP_001762479 SWR1 complex protein, SNF2 family DNA-dependent ATPase, partial [Physcomitrella patens subsp. patens]

MQFLEANKDKKVQPKSKKPARPKTHWDFVIEEMTWLAKGKPTTKGWIFLQSHEECTKCSKKFLKILLVHQEEEQRMRRVASNIAKDVKKFWLKVDKLVSYKQQLLVEERKKKALDKHLDFLLGQTERYSTMLAENLADNTTAHEPQSHQPFPSLCKEVSSTDIQVLSVLSEQINVSGEAEKLEGAVRMEVEGDDEFIVEDKDEQEDDEATLEADEALITEEERKEELLALQIESELPLEDLLIAYKLMRDQEDDDDKEEKVVEGPTYVTPVEESKKGTSAAAHDIGSLGQPDSVEGCLVASETDENLAKLVVGVTHRNTIGAFLTQVKPQKRRHLSPKSLQHQIGELQVCTADDDERTLEEEERIAMEEGDRNINEINELKLESEMPLEELLAKYRSGISGSSSDDEDDERGVNYFSEAIERMGEPSTSGRDYQRLSSTEVDREMNNFNVINTVETLDVGENKKSEDRLADYAAAAQSAQPTGYTFSTTQVKTKLPFLLKHSLREYQHIGLDWLVTMYEKRLNGILADEMGLGKTIMTIALLAHLACEKGVWGPHLIVVPTSVMLNWETEFMKWCPAFKILTYFGNAKERKLKRQGWSRPNSFHVCITTYRLVIQDAKAFKRKKWKYLILDEAHLIKNWKSQRWQTLLNFNSKRRILLTGTPLQNDLMELWSLMHFLMPHVFQSHQEFRDWFCNPITGMVEGEDQVNKELVDRLHNVLRPFLLRRLKKDVEKQLPGKFEHVIRCRLSKRQRNLYEDFMASSDTQATLSSGNFFGLINVLMQLRKVCNHPDLFEGRPIVSSFDMTGIKLHLSSAACSATAMGPFDGIDLGTLNLQFSRLSGTMTKWEAHEVGMLKAPGPLIVELAGTGEDTWDRHQSKHKPSKEVRTVIQEIHSSLRDNRERQRRERLLAISVLNEFRCNQQPLYGADFLKSAEIIHPVYDVHKVNGNPRQYLEFSSILSDIVQLPLTRCESMIDLITAFVFAIPAARAPHPVAWCSHLIPASIDLKQSISEEVLQRANSMLVPLRPVFVRKQLFFPDRRLLQFDCGKLQELAVLLRRLKSQGHRALIFTQMTKMLDVLESFINLYGYTYMRLDGSTKPEQRQVLMQRFNTNPKIFLFILSTRSGGVGINLVGADTVIFYDSDWNPAMDLQAQDRCHRIGQTREVHIYRLISESTIEENILKKANQKRILDDLVIQSGSYNTEFFKKLDPMELFSGLKEIKVRGTPDKKLTSFITSSIKELSNAEVDAALKNAEDEADYMAMKRVEQEEAAENQEFTEELFAGNVDEEDLADDLDESGKSRKLQTDIAGVDGSRVGERAPEDGGTAIITEVIPFGEFSILPADPDEEMDMLADVRQMAAAAAASGRGSISFEDQLRPVERYAMRFLELWDPRVDSMAVVAQVSFEEKEWELDQLEKLKEEQEAEMDEDNEPLFYETWDTALADEAYRQQVDILAQQQVCKRALKLRIKALAEAAATVRGAENFTLKSKGKKKLKKAKFKTLAEGSLMTGTEDISIREEFQVDRSSDPSYRDQHSDMMLLPHRSLSQRKRKAPMLLKEEVRVEAHTKKLKKNHFGKDRRSSGSTLQESVDQPGGLSNSGVHGGTVLAGKDKRGKLTILGMPPKKGPLIMLEKERKKDSLRSQDHLPPASPWTHGEDAVLCAVVHEYGGNWQLASDALAGGPDGGVYRGRHRHPVYCRERFRELLAQNAAAASGDPVSERSALSAATNAQLKVTEEHTKRLLNAVLQLPDKELLLQRHFVAALAAVEKWRKVAAKHGSEKLLT

>XP_002998828 conserved hypothetical protein [Phytophthora infestans T30-4]

MQTRRDEEASNRKALHQQPVLMATSTPAVTDHSSSNGLASIKRPTSTPRSSSPSVDSTNAEFEGNLRESVRLHREVNKRTLSVTRKRQLPRLPTPARGKSHWDFLLEEMKWMATDFAQERNWKRVIQHRLAADVIVAQKAESVRQEHENRQMARGIALQISAFWRTMERIAARSRVRFEAAGTDQGEIDDPMSGDAEDDGKRSKSEDLDVASTSDGVLALKYHTIEVARSNGANVKKGEAKADAVLASAKTHMTYIVSAGKRARSAMTSSTAEDADKSSTWSLREAAFRDLQARCTALQTTGGPTIILAAFQLLALRWMLDLYSSGLNIFLNDQLGMGKATTIVAFLSLIEIVSSQKQHGDETTVTTEDQVTGPHLIIVSEEELHKWRYFLRIWHPGRRIQLYDGVGSTSSCREQLQREWKRKSRAQAHKTEDAFLTYDDDDELDLDELYDPIYCVLCPVSAFAQDRDAFVAFTNWQMVVVENEHGAQFEDAACVSALQQLRQRQRRVLCNGQSLENWKNTALRLQYAEFLVREASDDHVTERRHRVEKWPVQELNVERASVAEVMQLYGKSSSVRASLWKAASQEDTPQYMNALLVALSCLSLRRVRSEVESELGKIDEISLSCQPSLSQRTQYRNVLTGFAATLDTCSGREERLSVWLQLLLRLREICNCVDLVNDMDKLGHADMRLLTSCSAKLQVLEPLLRRLLLQEGKKVVIYCQFNAMFPVLEFFLSLLDINYVRVTGSVAMQRRALCHFADRAVVRVALASTRLSMSNGRRAVSVFGSEAIIVVDGDWNATCDAKLRASWAKMAVGGADTLPVYRLHCENTIEASLLRVGASLTEKVFGEMSPQELLAVPSDMTPSLTIEKPSWWSSTATSSGGNGLTDTGAMARLTQVAQQVESDEKYCGDANELGAPLIVHNVDLDAEEHLLLANTDELTPVEWYAVNYVHNVTDKKQEGEGKTVESAESNEEESAAFWNDSNVFLQSDERSFEKLATLEANRQWQEGDATSQLFFTLDTRYNGVVDIALEKVFMQMRMEGMETHFDVYKPPRTLQSNQMTFRVSYRVKRKLESQLGGIKSASQKEMRVDLEGIPVPDVSEFADDDFWGDTNLDALDSAEWDDPALLSGILGAGVGTGTGASSTASSGTASSGTTTAASATGADASKTGSQKSSKKSKTSTGRARKSSMSSDSGRDAWSVHDDIILKKLFELYGANWTLIAQVFNSSTAVSRFVCKKRSPRQCYDRYGKIISGSLANTGASVLKDNKLSTLKAQRAAAAANVAAAQLTSVVLDTRIGLPHSELLLTFPQCHSFPGLPPASIVSELSLVEMTLAQRKKLAIDKKPTGMDELKSIKTSFDAIIQCMKHKTSPPPIPIPVGATESSSDLGTKASLTAASETPTTSAPVVATPTKSTTKKATVSPSVTAKAMSVAVPPPHKSHTDMISLLPTTVLGPDEVIKRSKEAAVVAVQAAAAVASVGRDGSPLSASGDAMLGAGSNFGAAVSGSMPRRSHAASLTSEMTAVGVLSTSSIAPTVPATGAATTASNTGTWGGNMVQTNGLNASASMELNAASVLPGTEAPRAPMPVTTSTLLHVLDRMPEIKNKIQSILNRTDCSESQKVAMIARLLSNTNAINNTNALASPASTPNSNTVLSALTADAGMLNSSDMLIDTDSPLPMPASLDASSSSNVTPSSGQFQSPPTSQP

- 1. HSA-SANT proteins

>NP_010646 Eaf1p [Saccharomyces cerevisiae S288c]

MSSRPSSAVPNSASLSEDQSSDRSKFPKADDLIDERDRKLTELYCVSRLNQLLELTDENKLRKEIDAFLKKNDIRRGIRFDEASLPKLLHTAATPITKKKLKDVNLINVPNQRLSDSKMSRELPENSENVSVKSESHFVPSHDNSIRENMMDSLRPAEKTGGMWNKRPLESTMGGEEERHEKRQKMQSQSLESSNNSEMASLPISPRPPVPNALAHYTYYENIEYPPADPTEVQPAVKFKDPLIKNIMAKEIDTSDHYNENNVDALETVFLLMNDYIPSKIPQALPLAELKYMSQTLPLINLIPRAHKALTTNIINNALNEARITVVGSRIEELRRLGLWSLRQPKRFIDPWKQHNTHQNILLEEAKWMQADFKEGHKYKVAICTAMAQAIKDYWTYGEICCVKRKTLLPGKENKLSDDGRISEKSGRPSDTSRNDSDISIAGKDDIGIIANVDDITEKESAAANDNDENGKNEAGAKSDFDFADGLLSQEGAHDQIISSIDTKLLLKKPSSSSEVVLIQHEVAASSALIETEESKKELAPPFKLSIFVDELNTFEKTLIQDLPLYNGINEERPKKDDSLPFIPISKSVVSLDDNGFYKLLERQLIDEEPSISQLSKRRGMFYGNRRNHYLRPPAVPSLRYLQNRTPTIWLSEDDQELVKNINTYGYNWELISAHMTHRLTYSYLSNIERRTPWQCFERFVQLNERFNFSDLKGPRAHSAQQWLIEAHKFQQRQNRRISPLGVNTESIQRGHRRLRWASMFEAIRKCMKKRENTPRPNPTQPRKPLDCKNMKVPTPAEMSLLKAQRDEALRRDIQLRRTVKNRLQQRQQQSQQAHSSRAQSPIPSNGKSSSNLARNGQASAPRPNQKQYTEQDIIESYSRKLLEQKPDIGPEMALKAAKNYYRTLREQQQQLKQHQIQQQRQQLQEESSHVQQLQQLQPGSQAPPPKSSPSQSSLSNISNINSAPRIKSPTPQEILQRFQKQ

>NP_588036 NuA4 histone acetyltransferase complex subunit Vid21 [Schizosaccharomyces pombe 972h-]

MKQKETKTSQIALVDGEKLSITDSFASLFTLDEEEENDSVDNEEIKLTKEKHEKLLLLFWLHCKFPNGLEWLHSSSDLLPEQVSEWFNFYQKYRFKRGRNFNLSARESVTPIVEEPIVPEEPDNLEGVSEETPLKETSLELSEEEIITSKSPIPSPETIHKNIDVEEKETIEPTTPVKEVETTAHAEEEKGPLTPDSEYAARQLTEELANKSSQEEGVDKQLRVVEATEKEHEEDGNEENVTVTKPVEVATDQVESKEVKKKEVSETTEPTAPPVTVAEVLEIEDKVPKVDEVEEVHSPEAKVTENDVENVQSGIDIEKTIQLLNNQEIPSEQQIISVDKATESPVQEVAVDVNEKPVDEIVEPSKLQMENKLPSEKSPTIDRTGVEAPLFELSVSMPLTLIPPSKFSEPVKPELSSEAWLLRTEMSPLHLRLKNAHKYVLSDNWSHAYREEIVRQSLHHLTVAKEKGIWSFRQPKRQNEMPRLKTHRDYVLDEMQWMSIDFSQERKWKIILAHRMANWVMDYHQASDKCTVCTPASLSKNKKPYMQENEHQKDSHEETFNEQIVSHFNLNDNNNNKVLSIPRDSLQFYNAVFSDDIFVTTNSEQIQNCVLNVPMYGPPTENNEYCEEISEKYPITPVSRFAYAKTKLKSTCAKASRKRLFNQLELSPPESFMEKKARSDENQLDGNKIKDDNQKLSSVGTFSVRPPYPPSSKDIRPEAPWLPEEDELLLLLLRRYSFNWEFVASRLTPPGLYIPLAEKRTAWDCFERWIQVDPRAANVQLTGSHARLAQQKLDESLRHSDKVSQHLSLRDEGTPNHLIKHNSYFLLPTVSRHYRPITIFEAIRKILKKREFAKKPTMTKRAIAPSAASTEKLPPVPSPLELSRLKSEREAQIQQIQAQRNFAQLQSQNRALRPQNAAVAAGAQQHNQQLAAFQAVAASQNSSNNSSAGVSPIAGRMVPRLQPYAVSSSLKLTPEQIHQLQQRKQTVPTTERTQ

>XP_762001 hypothetical protein UM05854.1 [Ustilago maydis 521]

MSTEPVSSLALPAPAILPTTPIPASTQQASSLVSDQIQEASTASQKSVTSITQPPIPSQQQPPQHITANHPASIAAAATVALSATLLNPNEWNALASTSMVTLDDLRSAFLEQRQQQLKDLERSHRESLREMIFMSENYDKEDVAGSPWKLGQSLQVAEDDAREHVKSFLDQHRLALDPKMSIKDHILEQPKLLRQRVSQELKTGPIELAPPPTPTSATAPSAGEAQRDRDLKREIEQNNLHRSQAVARAAAAVQAHAIAEGVAKIKPLTITAAESQGVRVAPLQPGQVPSFVIESTTPIAHIVASSDSSTAVLVASDAQKVDSHTTMSAAEAVTPAIKVESTDNIDASPSKASHALEKAVSPVPGLARNASTQSTSLRVPPSPMVVPTIPENITAPPLHPTLQVLAPNPLSVTYAASLRPLPPDPTRRITGAGLSGGAIHHRSGRKITSLSNHSNVNSYSSIAAAKIGPAGSGQADLYRWYVRARASPGAGMVGKADKCLMTSDWRVAFNEQRFVRAMARIEKLKAQGEWSFRQPKKQKGPVVRKAHWDHLLEEMKWLQTDFREERRWKMTVAFHLAHEVAAWHRARTPAERAQFCVHVQRSYRHRTISDNVEQHNTDKLSAKEVVSSSQPFQAAAASLDDVEMTHSTSSSHIQKDGEPATVKAESGQPVEDADVTMDGGADADEAVTQAVESALQTTNEAASARAEEMDADGEDDADTDANDVEAATAALVSGEPSKPPKVEVVREPLSQPQPMPTLADVSVPTTSSATPTVPASSTPTTAANDKLVHALRSQPKDADSTLTAEMPPQLLATLRAPIFSTSVTTTVVSPAALLDSLNPEAAAALLGIEVSDLANAADLLEPGSLSFSKMFPELPLYGGVSLPESNSKSDRRWDEGSLNQPPRLTHVTKLLDSRPLLVSTLEPSKNRANGRWLADSDWVVAAEQSDPLRGVTDGADTALPPMPGSLLFARKSNRAAKDASGPASTTPAEPASPDARAALFVWTPDEDNYLMTLAKQYHNNWALVADLFNSTRLNTATDKREAWDCYDRCKRIEQAAAEGKPPPGPPPMPAPAADADKDSKKGDGKDADASSSKRDKLSKKSGSKHDGSKRKQRRSNLMEVMRRSAKRREATKQAQQTQQTKKVNLNTHETHAQIKAGPAITPQSLSALKTERDQAALRQYYEQQRAQLAYQQQQQQQQRLLAQQAQAQRMAQQQGTAGTSAPAATAVTQAGKAGTAIVAGGTGGVGNVSLLPAGTPAASAGPAGQAQQKAATGQGTNQAIAQQTQQQQQQPQSQQTAQMQVQQPPAAAAAAQQQQQQQVQLQSQLQASQAAQMQNLYAQMQQQQQQQQRQQQQQLGQAAAGLAQVRPGVGALTQQQLATLTPQQQQQYHAQLAAATAAAQQQQLRSQMAAVAAAQGGQAGFQLPNAQAQAQAQAQAQFQLMQQQQQQQNVMQNQGRPAMAQQAQQAALQMYQQQQRQAQMRPPQPTQPFAARPNARPGTAASSQAKPQAAARSATPAPPMPATVQALQQQLAISLATSNLSAEQINGLAIQLYKQAQQQQQQQQQQQQQQQQQQQQQQQQQQQQQQQQQQQQQQQQQQQPQQPAPTQQRPPPQQLLNQAIQALAAQTQKNQQPAPTVHGVTGSPVRPPTPRPS

>NP_189131 Helicase/SANT-associated, DNA binding protein [Arabidopsis thaliana]

MHGSVSGYLLVNAEVDSMGGVIDSGGGIGVKTSPRRTAIEKAQAELRQEYDVREERRRELEFLEKGGNPLDFKFGIATSHSVQSTSLTDQQAEHFVNSEVKDSFALTASPHGDSVESSGRPGVPTISEPNTADNLLLFDSENKSVEGERNLRHPNRQNRTSESERSSKAHTNQNTKETEDSAIFRPYARRNRSKISRDPARSSSTDLVQNRGGLATSISIRRGSVEGKGCIPEAANQKDMHTTSVSCPVFANSNGNIVPKNRVSSNSLNTKVDGEPVVRESTAGSKTSLLKDEADISYSKSSAYLPVGESGLAGEKAQLVSTGGSPKAATIAGQKNSSTQLNGLRDSTVEEESLTNRGATGTNGLESESSHANNVEVNVDNERDLYKVDKLDSDEISMQKTLRVEGLLDQTVGEMTKTKIEDETGQSTTIISECIPECEMQMKSVKIENQSHRSTAEMQTKEKSSETEKRLQDGLVVLENDSKVGSILSENPSSTLCSGIPQASVDTSSCTVGNSLLSGTDIEALKHQPSSDAVMLDTVKEDAILEEARIIQAKKKRIAELSCGTAPVEVREKSQWDFVLEEMAWLANDFAQERLWKMTAATQICHRVALTCQLRFEERNQHRKLKKIASVLSYAILQFWSSVEAEVPGELEETSLGIVKETCQESNCLNGIRCLAAGVKEYASRFLKYNNSSISYHSAALSTPDNMCDPEILDISMVDQLTEASLFYSVPSGAMEVYLKSIESHLTRCEKSGSSMQEEVDTSAYDTAGDIGYNVTAFDEDEGETSTYYLPGAFESSRSFNISHKKRKNLMKSHSARSYDLGDDLPYVNNTGGSNSSSLIVKRPDSNINAGSVPTRRVRTASRHRVVSPFGCATTGNLPVPSKTDASSGDTSSFQDEYSSLHGGSAVQKGTEVESSVNFEKLLPYDMAETSGKPKKKKKTHQGSAYDQTWHLNPSVHVEQKDHWKKRPENNFDMNGLYGPHSAKKQKTTKQLVENNFDMAIPHTGSIPSPAASQMSNMSNPNKSIKFIGGRDRGRKIKGLKISPGQHGSGNPWSLFEDQALVVLVHDMGPNWELISDAMNSTLKIKYIYRNPTECKDRHKILMDKTAGDGADSAEDSGNSQSYPSTLPGIPKGSARQLFQRLQGPMEEDTLKSHFEKICLIGKKLHYRKTQNDGRDPKQIVPVHNSQVMALSQVFPNNLNGGVLTPLDVCDASTSGQDVFSLENPGLPMLNQGTPVLPTSGAHPSTPGSSGVVLSNNLPTTSGLQSASVRDGRFNVPRGSLPLDEQHRLQQFNQTLSGRNLQQPSLSTPAAVSGSDRGHRMVPGGNAMGVSGMNRNTPMSRPGFQGMASAAMPNTGNMHTSGMVGIPNTGNIHSGGGASQGNSMIRPREAVQHMMRMQAAQGNSPGIPAFSNLSSGFTNQTTPVQAYPGHLSQQHQMSPQSHVLGNSHHPHLQSPSQATGAQQEAFAIRQRQIHQRYLQQQQQQFPASGSMMPHVQQPQGSSVSSSSQNSPQTQPPVSPQPLSMPPVSPSPNINAMAQQKPQKSQLALHGLGRSPQSGTSGVNNQAGKQRQRQLQQSARQHPHQRQPTQGQQLNKQLKGMGRGNMIHQNITVDQSHLNGLTMPQGNQATEKGEIAVSVRPDQQSSVGTTTSTDLQSKPFVSPLSSNHSQQLPKSFPGALSPSPQQQMQLHSDNSIQGQSSPATPCNILSTSSLSIAPAVAPSNHQHLLIHQKQRNQVQSTAQRVVQHNHLGNSELSKKSQAECMPRVPQSVTNTTQTASMGTTKGMPQASNDLKNIKAVGSTAVPALEPPSCVASVQSTASKVVNNSNTDSAGNDPVSTPNQGLAQKHGIKGVTQRQQQSLPSEEKRPKLPEKPTVQNQKHLASEEQPHLEEAQELSSSKPPDTKVE

>XP_003565807 PREDICTED: uncharacterized protein LOC100845890 [Brachypodium distachyon]

MGGMVDYGVSVSTKSSPRSAAIEKAQEELRHQYDVREERRRELDFLEKGGNPLDFKFGYVESVSLQSTSLTDQIAEQNGISEAKGSFAFAASTHGDSVESSGKPGSSLCRETNMADNLMLFGGDNNGITKEKIVKRGSRRTNAGQLRQSSDGHNNAKRAEGSGLSRLGVKSQAYVRRNRSKPSRDSANVTSITSPVVPAIASEPKDAKATTKEKQADDLGVLYGSSLNRAGPKCEKEPNAASDEHMAMELSGTRTIHEGHCTVKHEARQGDHNNSVATEAVLNDVNGKQQPDGCGEIAAAGASIETPDATSKPVLRSSYSSLSTHDERETCADEKVDNDHLDEDMAHIHVGELGNISKGPVCAVEAATVHKNAMDSRCEDMTITIGDHADDSNLVPMKIDRKSHEDLDISGISSKDVNEGGQLEGLSRLSSVREKSCSEQPELSTTVLVKDEMEISDDAIVEQKDTSCPEPSHLINNKETPDLENNSSHGDSNSAHPTVVGPALNTFPDSTPSSKTYGSNVVSEIKKCGESLSTMTNKEYEDSILRKACLIEVGLKRACERSPCNISLEKRRKGHWDFVLEEMAWMANDFMQERLWRITAASQVCHWIASDGLAKFDQASIYRKQKTVIRSLAKGVMSFWRSAEAVLTAAGTAKVMQKDDSDMLGETKPTGIKADNKQGNESMETEHSIRPLRSQIQDYAVRFLEYNSQASDSVFTEAPPTPDRLNEFGILKVSTQLSQVSLFYAVPPGAMLAYRESVESLFMYHKNIGTGLKYNYEASVCDSAADLAQDNAYDEDEGETCTYLLAETYDGGLLPKMGHKKKHIMRQRINSARPYEIGTDVSYEPCLERKSGNQQFFSNGKRPSSFVGIPTKRIRTAARQRVVSPFPTSVGGTPHVKTDASSGDTDSYQDDQSSLHGGSLSWKNADYESTVDFDRQMPYDGSEAWTKANKKKKHKSTGYKTAQNTANSYASAAVKKDYLKKRSETQQFYSNGTIVTNGGQHAFKKLKMMKQGIDISQEASPVASQMSNMANPAKIIKIITNRDRGRKVKALKMSSGGGWSNFEDQALVVLVHDLGQNWELVSDAINNIVQFKSVHRQPKECKERHKVLVDKSSGDGADSAEDSGSSQHYQFTIPGIPKGSARQLFQRLQGPFEEENLKSHFEKIALLMPQVQSRRRQVNSRELKPIIQPHSSHVAALSQACPNNLSGSTLMPLDLCDTISPNLDAITPGSGYQGSHANGLTLSNHHGSIGTPSPTPNSRLQGSPGMVLGSNLSSPATLSAPSRDSQKYGAPRSTSLQVDDEQQKIQYNQVASGKNPQQAGVSAPGTCPAGVDRGAHVMPAVNGIGMTAGVNRGPSVQGVPNAVNVHSGAMSSLGNSVLRPRDPMQIRMVSQGSSRAAHFSSMNPPFSKAAAPSPVHQTQKLHQMSQPSHVLGNPHNPRAQGTNHSSSHQQSYAGHFAKERQFPQRMVPQQHNDPSGASAVPSVQNGPHIQQQNKAPVASSTPPSQPLHQQQQPPIQNPPDSFAPPNQPASTAEPKQKKQQGQQQVRQNQQQRNQASQQAKLMKSLGRGNMLIPQTPAVDAAPASAVSTCSKKQASDKNLMQHGQGSSPGNKASTSAIPQPGNQHMLYASLPQSPKQLPDTSSQGLMQGSPSHTLLAAPQPPVHSKPPSTTQQRQINPSQNSIQRMMMQQNLQMNSDCRMDAQIDQIQHNPVIPTTSISHGTESSSPGLPCMNQQKHEASANDVTSVTSTSKLICSPKDNLVGNGASLPSSSQELLQRKISGGLPMHGQDIGGQWHQQQSMQHLQPPHHQQTQHQQRPVVQGSLYAPSNSGSG

>XP_002986963 hypothetical protein SELMODRAFT_425792 [Selaginella moellendorffii]

MGGGSGAATNVDSIPSPRRAAIDKAQAELRLEVTVREELSKELDFLEKGGNPLDYKFGESLLPRFAAQVLSSELDDLCARNTNCSSLENGRYVEQETASCRVTGEVLENGSAGTPGHVSDGPVFKHRDHDEVLGVEGRKNQTSTKKLKQKEFGNGHAASKYLPCDTDQSSMCRFQVPPTCSADVQEVGTCVSPGNDGSVQDERKENKDISDDYARQKAMEQSTNEEINPASSPEAKQDGKGVEEQSEGDKTRDVARPSEPCPSGLSTDASVQKNEASDKQKPVSEKAQEDMILEAAETIKAAQKHTMEFSRHKPGSEPARQKSHWDFVLEEMSWMANDFMQERLWKTAAAAQVCRWTAQRLQQTKVADTDLKKNKKRIAHVLANIVTRYWQSAAALTQKPDELSIKKNDDCEAVPMDVDGPAPTEKRRPIHKYVMRASTDLELPKTPHFVPDSLFLMEQFPEESLVFIVSDGALETYRLSIEKNWDAFVSEKLLQDSLNDQRLSGICSELDLYGDDSRSFGSRTSPKLPDDDYGLHYMPGDLTISKKKRKRILKAQTPKAYDGLSQGGYCDGSNRLSSPAKRPTAGAASSIGVIPTKRMRTSAVVARQRAASTTLSPGAHRGNASSASTNSQQEDLLGFPDSRASEGDSVGPRKGGLGTSSLRFKKKKKTKAFHSDFSTDAISNSHLSKTDAELNQLEQDQLKRKSDQYSPLSSEHDTPGTPGSQGVVSMHPMKKLKSSKHSPDFAEAGPSATGLNLTTSSKIMRQNSARDRIRRNKATKGFIQSSINPPGVGIPWSATEDQAILALVHDLGPNWELVSDVLSSSSQLKGIFRKPKDCKDRHKSLLDRLSNDNGDNDEPGTSMPKGPMEEDTVKVHLERIVNIVQAHRSKQQQNDTLENQELLPVHASHLSTASQFSLSGVPLSPLDLCEHPMSNGDIPAHGYSLPQHPGLNMPGVMAPNGMRPNSAGLPLLPHLGSMVSPGSAAINAAAARDAQRMAASMRPLSAEEQRLRYTRMVTGRSLQPQANSPGNLPMASLPNSSDCSMPMLPSGNTAGLMGLNRGGLSLPRPTMPGMIPPGPSPGLMPPGGVALPASGSMMAGGIPSPANFMKRAREATQLIRSGRCTEEQKQHIIQELQHQAAQGNPQAVAALNTLSSNNMNLSNPMVSSPTQSFAPQQQQHLSPQQQSQQQQHQQYLAAVRLVNERQQQQQQQQQQQQKRIFQQPNNLLQQHQLGMLTQQQQPLLPSQQAQQLSPSHQQQQSQVLPGAQHPQQPTPTQMGQLSGQQKVQVPTQKQSLTKQPSFQQQQLLQVQQQSKVGKNNASGRGVATHSALMPGSQQTANSQLPSPPGTAQAGQAQQRSLAMQQAGKQASMSQPGKVSGQSQGGQLTQGTAGQTSQQKSLQKQSPQGASPVPAAPSTPPPQQQTTPATSAAAPPASQQPASTTTQAQIQRRQQQQQQQQQQQQPQSQQAPQARKSQQKQASALQAPGKNTTQPGLQPLGGSNSGSGPMSIGTSSSGLPPQAALAPSAAGSWKPGQNFPGMYNLTRSTSGGANQGSATTNGNGGAALNQVGTAAKLGIVPAVNGRTLAQQQQGSGAAQRPVQQQQQSLPVPVPVPTNTVNSTRPARPSSVLPSSPSFPGTPVPSLSHGVQPRQVHGGAQRPTTNGSSNPSVTASATAAPPSVVTSSSSPRHKHSVLKLSILLRMAPPNFFYSGSGSTKLEEIHGSRTHADRMECNKDDAVKAVDLAEKKFMLHDLAAARDFCVKALQLDPGLERGKQMLAVVEVHAAAAVRHHSLIILPSDLFGIGDHDWYAILRVDPRADDASIRTQYRKMARLLHPDKTRMNGAEEAIKLVNEAKTVLSDKNKKMIYDSIRSSLPSTSNDVSAPPPRTPPPPQPPPYGTPTFVAQCPFCMAQWWYYKTFENYVLLCACCLRNFIVVNFHHLAWGYPGSDSWYQRQEEEATMKRREMEWAETMRMEREREEERKREARIKEAEDSRAEFLRMKEREEERRREARIRQEKEDERRREARIKEERRREARINQDQEEERKREARIKHDQEERRREARIKERETRKQSEMDQRSKQGGAKSTNGNAGLVAVIVVLLVLGKGFILPAALLLALILVAFILSIRL

>XP_001766805 predicted protein [Physcomitrella patens subsp. patens]

MGAVAGTAAAINNNEPSPRGSAIEIAQAELRRDLAVREERKRELEVLEKRVDCLPFGRHTFMFSRSFVVCPGNLCCDHGCIEIEGGIGLRQNLTANGDASASSQLIVLGGDPDEDDCEIPLDKINQDPNWIAYRNGSISEKDGECVAGSALTSSVEFEHENEPIARCGLSLNQHAAEASCEDPISSYMSVQSTNLWVDPHVDSDGEERIWQSVASEGRVEQVEGSIAHVQKIGELGDADALPARHNVEDVSIIEASVGNSFRTRVEEGITGNQSHRIENKERLVSMEKDNSRYKVGANLDGLSRAVGGVNVLHANTSTDVPGGATLLMRAGLVKKNAVSRAGNHIRSATRDRLGQEEKRAGVYHVRIKYEDGVGENRARPFATDAELGCQHTSGAPSSQEELRAEGQAGAVVGGVPRALSRLSGVSASSNCIYPGVRIPWNKGQGPLSQMGSEGRKTGVQVKANEARADFILAEAQNLKVKKKFIGDWHSMEKSSEPARRKTHWDFVLEEMCWLAKDFFQERRWKIANAARICHSVAYERNKNEYLALDSVNRQRNRASFLALAVNKFWRMAEASLTKRKGGWVHDKSGKIEFCRNGKNEGEAVPIDVDIPDPALREALDKSEKLKKKNLYGVERYAVDYLKASGCGRMAQAVASVSHKLYWECNGILYFNDQERFSKEPLYYNVPSGAAEACRVSIEMERTRAEVEYEAKRVVAVAEAEAPAAGEASGEVSLGIDFGFSDFLVDGGSQEDEFQTNYMGEGGHARIYKKKRKKILKSNTGSKIEGIELQGLAYQSSDMSTPDRNVVTGKRSLPGESGPNNAPGSIPVKRQRSSAINLRPRASHLSISPGTTGVLSRTGPGSQNQQEGESFGMLDGSNRSDSANDTSNSKTHRIGDVGGFITNKWNKKQNPKTFPELSNSRSFEGGFPAGPPEKSADEDSHELLYHEQEQIKRKGETNWSFVVTSSDASLAFCLKLLRIAGTPTDTPETPGSQSLSGQQNVKKQKSSKQSETDAETATPAPAPNPPTLQQPPSLPSANKLSKQSSMRDRNRRLRLNKVPPSTPIGVGTPWSASEDQAILALVHDLGPNWELVSDVLSSNSQIKGIYRRPNQCKERHKSLTERSNLDGMESPEDSNSSQLQSLKGSVHGGTRVHGTPEEDSLKHLECIVQLVLKYRARKSSSENEDQKTSAAQHPSHGIAISQFCAGGPLNPLELCDRVANNGDANSHSYPIQPVQGSGLGPSLMPGSAMRPPSAGLPSVLPGSAGLHLGQSPVSSSAAMSAAAARDAQRFTTRLSPGEATRLRMAANNMSAYNARRLQQQAASVTAGLPILTGLPNPNDLPMLPTSTGAMMGALSRGLSMPRPGLSSMGSPVISNIVPAGLGGMVPPPGTLSSPSMSRRTANLLNMLRVIHVCGVPGNLEVWCKYYLCIKEHRLFQVGGCTEDQRVLILQQLQLLAQQGDNQAAAAFSNLGGDMATMIPSSPQVCKSTKVEGKFAGPPTVTLRPDEAIIILPSTSFDSVATASTAARSAAATTAAPPATSSTTTSSATTATAASSKSATKSTVTNLLFACLSCEQQQMQLQGNPTQAYYSAAAAVRMKEQQYRQQHQQKQRLMGTLPPTQAQYMLVSPHVQNLPAQQLGSKSHMLSQQPQRTHLGQQHQGSGLSSQSSQPALSTLNLQSSSASPTATGQSNSSAQQMPLPQQNQSVVQPGEATGSAKPPQGKQPQKYPTQTSQIMQHHQQHQQHTVRNSKGPMRGAIMQNLPSQTGQQSPNNLSGGSNQPLNETPGHMLIQPQLGQVQAGKASSGSHPSGSSQSHGSLAQGGTPSCSGVSQIKGLHQSQTQPQQGGVPDQQQGSSIQIATQVSLPQSGSLPTSSEQSQEPLATPSAQQTQQMQRRQMPQPQQPQRRLQSRHLSATPLVMPMPGQLGKTGMQQTNQDVSPQMVNHNPYQLGGTAMIGNPMSMSSPNSHVVSLPSTLNSSTIGAHSTQWKSSQSNNPLTGGLYNLTRANSPGSSQVSTMVISGPQLPTFAGTAAVPTLSTSGIHGVPTGSVPQNLAGKQCLSNIVLNQKIGVNPGVNGRVLSGAQQPTLSGQRSQQNGSVVISLPSGNSQMTAPGSSASTGGSPTSGAGVPPQGEGLLVPDTRDSAPGSSHSASSPMLIDNGQARQSGGVILSSPKTSVPRKPAVSAATAASDGISSGSTVSSTTSGPGPPNTEATPASSF

>XP_003064366 predicted protein [Micromonas pusilla CCMP1545]

MAGASKAKGAAAKAAEAKAAKAAAAAAAAAAEDDEPYVVDMKTIVRLEPAWVDFKPQPPPPLPPLPHEGETEEERRARLATDAETIARAKAIKDSKAVLGAKIAAGPYGSANRRKSHWDYVLVEMRWMASDFAAERDWKLAAARRMGSECAAAKGTPERRDLESEATEEGKKRCTAVAREVAAFWKKAWSRALEKPVPSAATLVPEKKPAAADGGDGGGGGDDAAAVKKETAAVAAEDGPRARRSAAAATASEDAAATPTKAEKDADAVVAIEGKPDVDATAAAAAAAAGAGAGAGAADAAATDAPAAGATDPNMQTPMATIRAKSIFPATPPPPPGVSVIEQWACVKLQGDIARLKKETIKEAMAEKEDGGKGKKGKAGKGKAAAAAAAAAAAKKGGGRSSRRGRGAKDDPESEEEEEEEDDDEEADPPAAAAAHASAAAANDDASTMPPPPPVYLPGGVPDATLDAELDFDAVAPQTPPRVLDDPPPLAYDCYASHAERWWAASIAAEAQKFADYEVRLRDWEESEKRRARAVVEAARAAAEAEAQRAHQHMLNERRRAHQAMLAQQRAAAAAAAGAAGKGKRVRGGGEDDDSEDDDMTLATLQNKGAGGSGGKKRKGGDVDAALGGRKKSHKKQKSGRGGVARPWSPTEDQLLCAIVHEFGSNWGLITDVFAASAPFKGVFRRAEQCRYRFQTLTQSAESDDPNAVRALDLDKTTARQVMQRALPVEDNTARTHFERAGLCAVKHAKLRRAAAAERAGDDYTRRAVAHASWGAHRGLRTSDPCDLADQARSIHWSPYDGALLEAARAQQQQQQAQQQQQAAAAQQQAAVQAAQMAGGAGPGPGGGGGPDGGGGGGPMDAAAAQREQQRRQQMQSQMQMQRQQQYQMQMQAQQQQQQQQQQQRQQAHQMQSQNVHMQQQQQQQAHMQQQMHMQQQMHMQAANANKGAGGVKGGAKPAGANNPPPNAPPPGAMQGTFQPVPGATATMEMPTIPGGAPASSPGRKGAGGGGGGGAAGAKGGGAGGPSPGLAPGSYKYGGGGGGGEKAEQPAGRGASTRGATTPRGKASKK

>XP_001388051 hypothetical protein [Cryptosporidium parvum Iowa II]

MLNRLYMAEKALSILRSGNAIPYPEPQGSVRSQWDFLLQEMTWMSKDYYEERRWKIYAARRLSQMVQNYWRKKMLNLERHISSKCSALIQSFWLGIADNVDPELIPNDLKPYTMVVQPAPVGEDGQANASKRRLYNYCIRQFERSLYECEKLNELTKTSNDNNKEKNVENVSENEIPASPMSIDIDHDNESCQWVVDEDLLLQLDPLVFVKTAIPPTASSTDIIDLDNIQNKGSSTNQNIGSSALGGTNANTNASNTGNTVGSSSKSSSNNSAITKYSEISDLFNEFSKKQETVVDLNDYRKYLENWADTVQVLLQGPGIINTRAEYRPPNFHLDSKNYLVAPRSNFLEMEDISLILSLFVLAVDHLPAFLNRYMASSPSNVVYRSSGGMISGSSSHESMVNDFNVESKRKSRRRAASNTKKISGVMPPPIQSSVSMPHGVSGGIGLPQSSAVSSVGGAMGGGVAGGAVSASGGISGPSTVPMVGDQSTSFDILAYADTWDTSEDIILVYFVSQYSSTPSCGVLSKKLVPTTNWNLIALSHNYFFGALYGRMKTAKQCQERWNFLNRDEIDGSSIEHVEKNVSKDKATEEEAVSNNGELSGNASNKEAASAIVMTEGNDTGNVTKSDIDRLMKREKQIRIMPNLNKRYIISSTLLKHLKEFSKSLEDSEGSENKAENGCPKVASEEVGGELTGTIDSSSMKDSKELEKMESNVTDGKAIYGDNLLYFKKKIIPKMTILDLIEKSNNVRSKTENHLESNSGSNNDNSTGSSILASQNNQGSEGTHNEVKSQNIVSNNEDGECTNRGNNKGSNNIKTVSSYQREQLELLSSIVSKFGANMRKRSLQNQQFQDSFQITSYLQTIQQNYGSNILQDGVLPPHQSHANLVAHVNQMLNQHIESTMANYNNTGGGNVGNNTSTSTNTGGVNTNPNNAGTSNANAGGNNINGNVNNNIGGGGSIVGNNSNGVINSGSGGPSTSSNMPNHTQGNSGTQMGGGAQLGSSNFTPLPSGIPAHLLAPGNLPSATSVEEYLSRLPGIDIQLKLIDYSLDRYRQSTMGGRYGGTGILMSTSSTCRKPQLPEFLLQRSVINSGANSGIVGGGQQGANIHGVGQHVGMMPPQQGMIHQGGGVSSGVGGDQQVPLQQSSYTPPVISGQPGQMILPNNQMIKMKKREINQKQVVAQASNIKRRKTNMIPPNSQPNSMMGPNGVQGEMIQSHGRIGMMQGIPPHMGGAGQGIPQPHSHPHSHHLQQQQPGSSSQLQPQQYQAQQYYPQQHPQQHPQQQQQHPQQQHHQQQIQQNQHSIPQVQHHHQQYQQQQQQQQQQQQQQQQQQQQQQQHQQQQHPHSHPHVQQQPIQGQTHTQHTSSHPSLGNSQMHLSMQQMQQQKYNQYHSQQVTPQAQSSHQQHHIGNPYQQTQNQGQYHSQQMGMMQGPVVGPDMHGGNSTAGAYGQSMGTKRGVQPGGMNHPSAQMMNTQGSHGAVGGIGSVSSSSIVGSNGNSGNSVGIGNSGNGGSMSRQVNTYMGYSGMQNRGGNGPGDMNSGMGEGVMGVGINVNGPGQAPGVASHQVSTSGRLIPPSSSMDGQRYSNASSNASSMNGVGVGGGGVSGSSSGLPSNENSISGTHKMVAGGVPQHQQQPQSMQKLGVSNPGYIGGGSTGTIGGNNSEMWVGAGNVDLVTGGGPTSSQSLPVVRHQSHSNIGSGIPHGTGMISGGGGGMHSDTHLGPSIKQNSMNQHYSNLNGDLNSNIGAAATSVGGALNGVQVTGGMDEGVSITGTGHSTTNSTNTGVISTSNLNINIADKTNNNINSNSQ

1. Alignment used in Figure 1

S._cerevisiae_Eaf1p MSSRPS-----------------SAV------------------------

S._pombe_Vid21 MKQKET-----------------KTSQIALV-------------------

P._patens_EAF1 MGAVAG-----------------TAAAIN---------------------

S._moellendorfii_EAF1 MGGGSG-----------------AATNVD---------------------

B._distachyon_EAF1 MGGMV-----------------DYGVSVS---------------------

A._thaliana_EAF1 MHGSVSGYLLVNAEVDSMGGVIDSGGGIG---------------------

A._thaliana_PIE1 MASKGG-----------------KSK-PD---------------------

B._distachyon_PIE1 MASKGA-----------------RSK-LD---------------------

S._moellendorfii_PIE1 MEAGGA-----------------AAK-PQ---------------------

P._patens_PIE1 MQFLEA-----------------NK-------------------------

H._sapiens_p400 MHHGTG-----------------PQNVQH-QLQRSRACPGSEGEEQPAHP

*

S._cerevisiae_Eaf1p ---------------------PNSAS-LSEDQSSDRSKF-PKADDLIDER

S._pombe_Vid21 -------------DGEKLSITDSFASLFTLDEEEENDSVDNEEIKLTKEK

P._patens_EAF1 -------------NNE-PS--PRGSA-IEIAQAE-----LRRDLAVREER

S._moellendorfii_EAF1 --------------SI-PS--PRRAA-IDKAQAE-----LRLEVTVREEL

B._distachyon_EAF1 --------------TK-SS--PRSAA-IEKAQEE-----LRHQYDVREER

A._thaliana_EAF1 --------------VK-TS--PRRTA-IEKAQAE-----LRQEYDVREER

A._thaliana_PIE1 --------------I---------VM-ASK--------------------

B._distachyon_PIE1 --------------H-----------------------------------

S._moellendorfii_PIE1 --------------L---------RC-YGR--------------------

P._patens_PIE1 --------------------------------------------------

H._sapiens_p400 NPPPSPAAPFAPSASP-SA--PQSPS-YQIQQLM-----NRS-PATGQNV

S._cerevisiae_Eaf1p DRKLTELYCVS--------RLNQLLELTDENKLRKEIDAFL---------

S._pombe_Vid21 HEKLLLLFWLHCKFPNGLEW--------------------L---------

P._patens_EAF1 KRELE--------------V--------------------L---------

S._moellendorfii_EAF1 SKELD--------------F--------------------L---------

B._distachyon_EAF1 RRELD--------------F--------------------L---------

A._thaliana_EAF1 RRELE--------------F--------------------L---------

A._thaliana_PIE1 --------------------------------------------------

B._distachyon_PIE1 --------------------------------------------------

S._moellendorfii_PIE1 --------------------------------------------------

P._patens_PIE1 --------------------------------------------------

H._sapiens_p400 NITLQ--------------S--------------------VGPVVGGNQQ

S._cerevisiae_Eaf1p ----------------KKNDIRRG---------IRFDEAS----------

S._pombe_Vid21 ----------------HSSSDLLPEQVSEWFNFYQKYRFKR-----GRNF

P._patens_EAF1 ----------------EKRVDCLP---------FGRHTFMFSRSFVV--C

S._moellendorfii_EAF1 ----------------EKGGNPLD---------YKFGESL-LPRFAA---

B._distachyon_EAF1 ----------------EKGGNPLD---------FKFGYVE-SVSLQSTSL

A._thaliana_EAF1 ----------------EKGGNPLD---------FKFGIAT-SHSVQSTSL

A._thaliana_PIE1 --------------------------------------------------

B._distachyon_PIE1 --------------------------------------------------

S._moellendorfii_PIE1 --------------------------------------------------

P._patens_PIE1 --------------------------------------------------

H._sapiens_p400 ITLAPLPLPSPTSPGFQFSAQPRR---------FEHGSPS-YIQVTS-PL

S._cerevisiae_Eaf1p ----------------------------------------LPK-------

S._pombe_Vid21 ------------------------------------NLSARES-------

P._patens_EAF1 PGNLCCDHGCIEI-----------EGGIG----LRQNLTANGDASASS--

S._moellendorfii_EAF1 --------------------------------------------------

B._distachyon_EAF1 TDQIAEQNGISEA-----------KGSFA----FAAS--THGDSVESSGK

A._thaliana_EAF1 TDQQAEHFVNSEV-----------KDSFA----LTAS--PHGDSVESSGR

A._thaliana_PIE1 --------------------------------------------------

B._distachyon_PIE1 --------------------------------------------------

S._moellendorfii_PIE1 --------------------------------------------------

P._patens_PIE1 --------------------------------------------------

H._sapiens_p400 SQQVQTQSPTQPSPGPGQALQNVRAGAPGPGLGLCS-SSPTGGFV-----

S._cerevisiae_Eaf1p -LLHTA-------ATPITKKKLKD-------------VNLINVPNQRLS-

S._pombe_Vid21 -VTPIV-------EEPIVPEEPDNLEG--------VS-------------

P._patens_EAF1 -------------QLIVLGGDPDEDDCEIPLDKINQDPNWIAYRNGSISE

S._moellendorfii_EAF1 ---------------QVLSSELDD--------LCARNTNCSSLENGRYVE

B._distachyon_EAF1 PGSSLCRETNMADNLMLFGGDNNGITKEKIVKRGSRRTNAGQLRQSSDGH

A._thaliana_EAF1 PGVPTISEPNTADNLLLFDSENKSVEGERNLRHPNRQNRTSESERSSKAH

A._thaliana_PIE1 --------------------------------------------------

B._distachyon_PIE1 --------------------------------------------------

S._moellendorfii_PIE1 --------------------------------------------------

P._patens_PIE1 --------------------------------------------------

H._sapiens_p400 -------------------------DASVLVRQISLSPS----SGGHFVF

S._cerevisiae_Eaf1p --------DSK---------------------------------------

S._pombe_Vid21 -------EETPLKETSLELSE-EEIITSKSPIPSPETIHKNID---VE--

P._patens_EAF1 -------K-------------DGECVAGSALTSSVEFEHENEPIARCGLS

S._moellendorfii_EAF1 -------QETASCRVTGEVLENGSAGTPGHVSDGPVFKHRD---------

B._distachyon_EAF1 N--NAKRA-------------EGSGLSRLGVKSQAYVR----------RN

A._thaliana_EAF1 TNQNTKET-------------EDSAI------FRPYAR----------RN

A._thaliana_PIE1 ------------------------------------------------SG

B._distachyon_PIE1 --------------------------------------------------

S._moellendorfii_PIE1 ------------------------------------------------RN

P._patens_PIE1 --------------------------------------------------

H._sapiens_p400 -------Q-------------DGSGLTQIAQGAQVQLQHPGTPITVRERR

S._cerevisiae_Eaf1p ------------------------------------------------MS

S._pombe_Vid21 --EKETIEPTTPVKEVE-TTAHAEEEKGPLT-------------------

P._patens_EAF1 LNQ----------------------------HAAEASCEDPISSYMS---

S._moellendorfii_EAF1 --------------------------------------------------

B._distachyon_EAF1 RSKPSRDSANVTSITSPVVPAIASEPKDAKATTKEKQADDLGVLYGSSLN

A._thaliana_EAF1 RSKISRDPARSSS-T-----------------------------------

A._thaliana_PIE1 KSKP----------------------------------------------

B._distachyon_PIE1 --------------------------------------------------

S._moellendorfii_PIE1 RSKSAKEEVSSPG-A-----------------------------------

P._patens_PIE1 --------------------------------------------------

H._sapiens_p400 PSQPHTQSGGTIHHLGPQSP-----------AAAGGAGLQPLASPSH---

S._cerevisiae_Eaf1p RELPENSENVSVKSESHFV----------PS-------------HDNSIR

S._pombe_Vid21 --------------------------------------------------

P._patens_EAF1 -----------VQSTNLW----------VDPHVDSDGE--ERIW--QSVA

S._moellendorfii_EAF1 ------------------------------------HD--------EVLG

B._distachyon_EAF1 RAGPKCEKEPNAASDEHMAMELSGTRTIHEGHCTVKHEARQGD-HNNSVA

A._thaliana_EAF1 ---------DLVQNRGGLATSISIRRGSVEGKGCIPEAANQKDMHTTSVS

A._thaliana_PIE1 ----------DNESR-----------------------------------

B._distachyon_PIE1 ------------ETR-----------------------------------

S._moellendorfii_PIE1 ---------DDARSN----GKLPGDKRL----------------------

P._patens_PIE1 --------------------------------------------------

H._sapiens_p400 -----------ITTAN------------LPPQISSII-------------

S._cerevisiae_Eaf1p -E----NMMDSLRPAEKTG-------------------------------

S._pombe_Vid21 -------------P-DS--E------------------YAARQLTEELAN

P._patens_EAF1 SEGRVEQVEGSIAHVQKIGE------------------L-----------

S._moellendorfii_EAF1 VEGRKNQTSTKKLKQKEF--------------------------------

B._distachyon_EAF1 TEAVLNDVNGKQQP-DGCGE------------------IAAAGASIETPD

A._thaliana_EAF1 C-PVFANSNGNIVP-KN--R------------------VSS--NSLNT-K

A._thaliana_PIE1 --------------------------------------------------

B._distachyon_PIE1 --------------------------------------------------

S._moellendorfii_PIE1 --------------------------------------------------

P._patens_PIE1 --------------------------------------------------

H._sapiens_p400 --------QGQLVQQQQVLQGPPLPRPLGFERTPGVLLP-----------

S._cerevisiae_Eaf1p ----------------------------------------------GMWN

S._pombe_Vid21 KSSQ---------------------------------------EEGV---

P._patens_EAF1 ----------------------------GDA--D---------------A

S._moellendorfii_EAF1 ----------------------------GNG--HAASKYLPCDTDQ---S

B._distachyon_EAF1 ATSKPVLRSSYSSLSTHDERETCADEKVDNDHLDEDMAHIHVGELGNISK

A._thaliana_EAF1 VDGEPVVRES-----TAGSKTSLLKDEAD-ISYSKSSAYLPVGESGLAGE

A._thaliana_PIE1 --------------------------------------------------

B._distachyon_PIE1 --------------------------------------------------

S._moellendorfii_PIE1 --------------------------------------------------

P._patens_PIE1 --------------------------------------------------

H._sapiens_p400 ----------------------------GAG--GAAGFGMTSPPPP---T

S._cerevisiae_Eaf1p KRPLESTMG-----------------------------------------

S._pombe_Vid21 ----DKQ-------------------------------------------

P._patens_EAF1 ----------------------LPARHNVED--VSIIEASVGNSFRTRVE

S._moellendorfii_EAF1 ----------------------SMCRFQVPPTC-SADVQEVGTC----VS

B._distachyon_EAF1 ---------GPV--------------CAVEA--ATVHKNAM----DSRCE

A._thaliana_EAF1 KAQLVSTGGSPKAATIAGQKNSSTQLNGLRD--STVEEESL----TNRGA

A._thaliana_PIE1 --------------------------------------------------

B._distachyon_PIE1 --------------------------------------------------

S._moellendorfii_PIE1 ------------------------------------VEDSVGQ-------

P._patens_PIE1 --------------------------------------------------

H._sapiens_p400 ----------------------SPSRTAVPPGLSSLPLTSVGNTGMKKVP

S._cerevisiae_Eaf1p --------------------------------------------------

S._pombe_Vid21 --------------------------------------------------

P._patens_EAF1 E-------------------------------------------------

S._moellendorfii_EAF1 P-------------------------------------------------

B._distachyon_EAF1 D-------------------------------------------------

A._thaliana_EAF1 T-------------------------------------------------

A._thaliana_PIE1 --------------------------------------------------

B._distachyon_PIE1 --------------------------------------------------

S._moellendorfii_PIE1 --------------------------------------------------

P._patens_PIE1 --------------------------------------------------

H._sapiens_p400 KKLEEIPPASPEMAQMRKQCLDYHYQEMQALKEVFKEYLIELFFLQHFQG

S._cerevisiae_Eaf1p --------------------------GEEERHEKRQ--KMQSQSLESSN-

S._pombe_Vid21 -------------------LRVV--EATEKEHEEDG-N-E----------

P._patens_EAF1 ---------------------GITG-NQSHRIENKE--RLVSMEKDNSR-

S._moellendorfii_EAF1 ---------------------GNDGSVQDERKENKD------ISDDY---

B._distachyon_EAF1 ---------------------MTIT-IGDHADD---S-NLVPMKIDRKSH

A._thaliana_EAF1 ---------------------GTNG-LESESSH---A-NNVEVNVDNERD

A._thaliana_PIE1 --------------------------------------------------

B._distachyon_PIE1 --------------------------------------------------

S._moellendorfii_PIE1 --------------------------------------------------

P._patens_PIE1 --------------------------------------------------

H._sapiens_p400 NMMDFLAFKKKHYAPLQAYLRQNDLDIEEEEEEEEEEEEKSEVINDEQ--

S._cerevisiae_Eaf1p --------------NSEMASLPIS-PRPPVPNALAHYTYYENIEYPPADP

S._pombe_Vid21 ----------ENVT--VTKPVEVATDQVESK--EVKKKEVSETTEPTAPP

P._patens_EAF1 --------------YKVGANLDGLS-RAVGGVNVLHANTSTDVPGGAT-L

S._moellendorfii_EAF1 --------------------------------------------------

B._distachyon_EAF1 EDLDISGISSKDV--NEGGQLEGLS-R-LSS---VREKSCSEQPELST-T

A._thaliana_EAF1 -LYKVDKLDSDEISMQKTLRVEGLLDQTVGE---MTKTKIEDETGQST-T

A._thaliana_PIE1 --------------------------------------------------

B._distachyon_PIE1 --------------------------------------------------

S._moellendorfii_PIE1 --------------------------------------------------

P._patens_PIE1 --------------------------------------------------

H._sapiens_p400 --------------------------QALAGSLV----------AGAG-S

S._cerevisiae_Eaf1p TEV--QPA--VKFKDPL-----IKNI---MAKEIDTSD------------

S._pombe_Vid21 VTV--AEVLEIEDKVPK-----VDEVEEVHSPEAKVTENDVENVQSGI--

P._patens_EAF1 LMRAG-----LVKKNAVSRAGNH--IRSATRDRLGQE-------------

S._moellendorfii_EAF1 ------------ARQKAMEQSTNEEINPASSPEAKQDGKGVEEQSEG---

B._distachyon_EAF1 VLV--KD--EMEISDDA-----IVEQKDTSCPEPSHLI------------

A._thaliana_EAF1 IISECIPECEMQMKSVK-----IENQSHRSTAEMQTKE------------

A._thaliana_PIE1 --------------------------------------------------

B._distachyon_PIE1 --------------------------------------------------

S._moellendorfii_PIE1 --------------EKAPGD---SKLGLASEESKLNAGKGAVT-------

P._patens_PIE1 --------------------------------------------------

H._sapiens_p400 TVETD-----LFKRQQAMPSTGM--AEQSKRPRLEVGHQGVVFQHPGADA

S._cerevisiae_Eaf1p --------------------------------------------------

S._pombe_Vid21 --------------------------------------------------

P._patens_EAF1 ---------------------EKRAGVYHVRIKYED--------------

S._moellendorfii_EAF1 --------------------------------------------------

B._distachyon_EAF1 ----------------------------------NNKETPD---------

A._thaliana_EAF1 ----------------------------------KSSETEK---------

A._thaliana_PIE1 --------------------------------------------------

B._distachyon_PIE1 --------------------------------------------------

S._moellendorfii_PIE1 --------------------------------------------------

P._patens_PIE1 --------------------------------------------------

H._sapiens_p400 GVPLQQLMPTAQGGMPPTPQAAQLAGQRQSQQQYDPSTGPPVQNAASLHT

S._cerevisiae_Eaf1p --------------------------------------------------

S._pombe_Vid21 --------------------------------------------------

P._patens_EAF1 --------------------------------------------------

S._moellendorfii_EAF1 --------------------------------------------------

B._distachyon_EAF1 --------------------------------------------------

A._thaliana_EAF1 --------------------------------------------------

A._thaliana_PIE1 --------------------------------------------------

B._distachyon_PIE1 --------------------------------------------------

S._moellendorfii_PIE1 --------------------------------------------------

P._patens_PIE1 --------------------------------------------------

H._sapiens_p400 PLPQLPGRLPPAGVPTAALSSALQFAQQPQVVEAQTQLQIPVKTQQPNVP

S._cerevisiae_Eaf1p -HYNENNVDA---LETVFLLMND--Y-IPS--------------------

S._pombe_Vid21 --------DI---EKTIQLLNNQ--E-IPSEQQIISVDKATESPVQEVAV

P._patens_EAF1 -GVGENRARP---FATDAELGCQHTSGAPS--------------------

S._moellendorfii_EAF1 --------------------------------------------------

B._distachyon_EAF1 -------------------LENN--SSH----------------------

A._thaliana_EAF1 --------RL---QDGLVVLEND--SKV----------------------

A._thaliana_PIE1 --------------------------------------------------

B._distachyon_PIE1 --------------------------------------------------

S._moellendorfii_PIE1 ----PSKLEP---LQ-----------------------------------

P._patens_PIE1 --------------------------------------------------

H._sapiens_p400 IPAPPSSQLPIPPSQPAQLALHVP---TPG--------------------

S._cerevisiae_Eaf1p --------------KI---------------------------PQALPLA

S._pombe_Vid21 DVNEKPVDEIVEPSKLQM-------------ENKLPSEKSPTIDRTGVEA

P._patens_EAF1 --------------SQ-------EELRA--------EGQAGAVVGGVPRA

S._moellendorfii_EAF1 ------------------------------DKTRDVARPSEPCPSGLSTD

B._distachyon_EAF1 -------------------------------GDSNSAHPTVVG------P

A._thaliana_EAF1 -------------------------------GSILSENPSSTLCSGIPQA

A._thaliana_PIE1 --------------------------------------------------

B._distachyon_PIE1 --------------------------------------------------

S._moellendorfii_PIE1 ---------------------------------SDAGKTQELCV------

P._patens_PIE1 --------------------------------------------------

H._sapiens_p400 --------------KVQVQASQLSSLPQMVASTRLPVDPAPPCPRPLPTS

S._cerevisiae_Eaf1p E-----------------LKYM-S---------QTLPLINL---------

S._pombe_Vid21 P-----------------LFEL-S---VSMPL-------TLIPPSKFSEP

P._patens_EAF1 L-----------------SRL-SG---VSASSNCIYPGV-RIPWNKGQGP

S._moellendorfii_EAF1 ----------------------------------------------A-SV

B._distachyon_EAF1 A-----------------LNTFPD---STP-S-------SKTYGSNVVSE

A._thaliana_EAF1 S-----------------VDTS-S---CTV-GN------SLLSGT-DIEA

A._thaliana_PIE1 --------------------------------------------------

B._distachyon_PIE1 --------------------------------------------------

S._moellendorfii_PIE1 ------------------------------------------VAT-EAKV

P._patens_PIE1 --------------------------------------------------

H._sapiens_p400 STSSLAPVSGSGPGPSPARSS-PVNRPSSATNKALSPVTSRTPGVVASAP

S._cerevisiae_Eaf1p ----------------------IPRAHKAL-TTNIINNALNEARITV--V

S._pombe_Vid21 VKPELSSEAWLLRTEMSPLHLRLKNAHKYV-LSDNWSHAYREEIVRQSL-

P._patens_EAF1 LSQM-GSEG-----------------RKTG-VQVKANE-ARADFILAEA-

S._moellendorfii_EAF1 QKNE-ASDK----------------------QKPVSEK-AQEDMILEAA-

B._distachyon_EAF1 IK-K-CGES----------------------LSTMTNK-EYEDSILRKA-

A._thaliana_EAF1 LK-H-QPSS----------------------DAVMLDT-VKEDAILEEA-

A._thaliana_PIE1 --------------------------------------------------

B._distachyon_PIE1 --------------------------------------------------

S._moellendorfii_PIE1 LD-E-TPQK-----------------DSVRFLNEEEEL-AYEDSVLSRA-

P._patens_PIE1 --------------------------------------------------

H._sapiens_p400 TKPQ-SPAQ-----------------NATS-SQDSSQDTLTEQITLENQV

S._cerevisiae_Eaf1p GSRIEELRRLGLWSL--RQPKRFIDPWKQHNTHQNILLEEAKWMQADFKE

S._pombe_Vid21 -HHLTVAKEKGIWSF--RQPKRQNE-MPRLKTHRDYVLDEMQWMSIDFSQ

P._patens_EAF1 -QNLKV-KKKFIGDW--HSMEKSSE-PARRKTHWDFVLEEMCWLAKDFFQ

S._moellendorfii_EAF1 -ETIKA-AQKHTMEF--SRHKPGSE-PARQKSHWDFVLEEMSWMANDFMQ

B._distachyon_EAF1 -CLIEV-GLKRACER--S-PCNISL-EKRRKGHWDFVLEEMAWMANDFMQ

A._thaliana_EAF1 -RIIQA-KKKRIAEL--S-CGTAPV-EVREKSQWDFVLEEMAWLANDFAQ

A._thaliana_PIE1 -------------AKRQKTLEAPKE-PRRPKTHWDHVLEEMAWLSKDFES

B._distachyon_PIE1 -------------ARRKKALEAPRE-PRKPKVHWDHVLGEMVWLAKEFES

S._moellendorfii_PIE1 -EEIWT-RQEALEQVKRRAPSKWKE-PTHAKSHWDYVLEEMVWLSKDFDK

P._patens_PIE1 ---------------DKKVQPKSKK-PARPKTHWDFVIEEMTWLAKGKPT

H._sapiens_p400 HQRIAELRKAGLWSQ--RRLPKLQE-APRPKSHWDYLLEEMQWMATDFAQ

: : : : :: * *:

S._cerevisiae_Eaf1p GHKYKVAICT---------------------------------AMAQAIK

S._pombe_Vid21 ERKWKIILAHRMANWVMDYHQA----------------------------

P._patens_EAF1 ERRWKIANAARICHSVAYERNKNE----YLALDSVNRQRNRASFLALAVN

S._moellendorfii_EAF1 ERLWKTAAAAQVCRWTAQRLQQTKV-AD---TDLKKNKKRIAHVLANIVT

B._distachyon_EAF1 ERLWRITAASQVCHWIASDGLA-KF-DQ---ASIYRKQKTVIRSLAKGVM

A._thaliana_EAF1 ERLWKMTAATQICHRVALTCQL-RF-EE---RNQHRKLKKIASVLSYAIL

A._thaliana_PIE1 ERKWKLAQAKKVALRASKGMLDQASREERKLKEEEQRLRKVALNISKDMK

B._distachyon_PIE1 ERKWKLSMAKKIAQRANMGIVDQATKDEKKQKEGEYRLRKVALNISKDVK

S._moellendorfii_PIE1 ERKWKVAQAKRVAMKVAKAKSEEESKGMRRLKGEEQRIRRIASSIAKEVK

P._patens_PIE1 TKGWIFLQSHEECTKCSKKFLKIL----LVHQEEEQRMRRVASNIAKDVK

H._sapiens_p400 ERRWKVAAAKKLVRTVVRHHEEKQLREERGKKEEQSRLRRIAASTAREIE

: : .

S._cerevisiae_Eaf1p DYWTYGEICCVKRKT-LLPGKEN-----------------------KL-S

S._pombe_Vid21 -----SDKCT-VCTPASLSKN---------KK------------------

P._patens_EAF1 KFWRMAEASLTKRKGGWVHDKSGKIEFCRNGKNEGEAVPIDVDIPDPA-L

S._moellendorfii_EAF1 RYWQSAAALTQKPDELSIKKNDD-----------CEAVPMDVDGPAPTEK

B._distachyon_EAF1 SFWRSAEAVLTAAGTAKVMQKDDSD-------MLGETKPTGIKADNKQ-G

A._thaliana_EAF1 QFWSSVEAEVPGELE-----------------------ETSLGIVK----

A._thaliana_PIE1 KFWMKVEKLVLYKHQLVRNEKKK---------------------------

B._distachyon_PIE1 KFWTKIEKLVLYKNQLEVEERKK---------------------------

S._moellendorfii_PIE1 KFWVKIEKLVVYKHQLAVEQKKK---------------------------

P._patens_PIE1 KFWLKVDKLVSYKQQLLVEERKK---------------------------

H._sapiens_p400 CFWSNIEQVVEIKLRVELEEKRK---------------------------

S._cerevisiae_Eaf1p DDGR---ISEKSGR--P-SDTSRNDS------------------------

S._pombe_Vid21 --------------------------------------------------

P._patens_EAF1 REALDKS---EKLK--KKNLYGVERYAV----------------------

S._moellendorfii_EAF1 RRPIHKY---VMRASTDLELPKTPHF------------------------

B._distachyon_EAF1 NESMETE---HSIR--P-LRSQIQDYAV----------------------

A._thaliana_EAF1 -ETCQESNCLNGIR--C-LAAGVKEYAS----------------------

A._thaliana_PIE1 -KAMDKQ---LE-----FLLGQTERYSTMLAENLVEPYK--QGQNT-PSK

B._distachyon_PIE1 -KALDKQ---LD-----FLLGQTERYSTMLAENLVDVPHL-QTQE---NG

S._moellendorfii_PIE1 -KALDKH---LN-----FLLGQTERYSSMLAVNLSGLPENNEDQKTQDT-

P._patens_PIE1 -KALDKH---LD-----FLLGQTERYSTMLAENLADNTTAHEPQSHQPFP

H._sapiens_p400 -KALNLQ---KV-----SRRGKELRPKGFDA-------------------

S._cerevisiae_Eaf1p ---------------------------------DISIAGKDDIGIIAN--

S._pombe_Vid21 -------------------------------------------PYMQENE

P._patens_EAF1 --------------------------------------------------

S._moellendorfii_EAF1 --------------------------------------------------

B._distachyon_EAF1 --------------------------------------------------

A._thaliana_EAF1 --------------------------------------------------

A._thaliana_PIE1 PLLTIESKSDEERAEQIPPEINSSAG----LESG--SPELDEDY-DLKSE

B._distachyon_PIE1 PLQTNLPSQEEEVAEE---NTNALMH----DDLD--KMEIDDDY-NSSLN

S._moellendorfii_PIE1 --------------------------GDLLDAAN--EPSTDKDF-TCD-Q

P._patens_PIE1 SLCKEVSSTDIQVLSVLSEQINVSGEAEKLEGAVRMEVEGDDEF-IVEDK

H._sapiens_p400 -----------------------LQE----SSLDSGMSGRKRKASISLTD

S._cerevisiae_Eaf1p ------VDDI-----TEKESAAANDNDENGKNEAGA--KSDFDFA-----

S._pombe_Vid21 HQKDSHEETF-----NEQIVSHFNLNDNNNNKVLSIP-RDSLQFY-----

P._patens_EAF1 -----------------------------------------------DYL

S._moellendorfii_EAF1 --------------------------------------------------

B._distachyon_EAF1 -----------------------------------------------RFL

A._thaliana_EAF1 -----------------------------------------------RFL

A._thaliana_PIE1 DETEDDEDTIEEDEKHFTKRERQE-ELEALQNEVDLPVEELLRRYTSGRV

B._distachyon_PIE1 EEPEDDEHTIDEDEAQITEAERNE-ELAALQAEADLPLDDILKLYAKTKV

S._moellendorfii_PIE1 MEEEDDEATLEADEALITEDERKE-ELNALQRESELPIEELVGLYAKGDN

P._patens_PIE1 DEQEDDEATLEADEALITEEERKE-ELLALQIESELPLEDLLIAYKLMRD

H._sapiens_p400 DEVDDEEETIEEEEANEGVVDHQT-ELSNLAKEAELPLLDLMKLYEGAFL

S._cerevisiae_Eaf1p --------------------------------------------------

S._pombe_Vid21 --------------------------------------------------

P._patens_EAF1 K-------------------------------------------------

S._moellendorfii_EAF1 --------------------------------------------------

B._distachyon_EAF1 E-------------------------------------------------

A._thaliana_EAF1 K-------------------------------------------------

A._thaliana_PIE1 SRETSPVKDENEDNLTSVSRVTSPVKDENQDNLASVGQDHGEDKNNLAAS

B._distachyon_PIE1 SRESSPDSK---DTFS-KSDLKNLMKDPSNQANGC-NHE----SG---GT

S._moellendorfii_PIE1 SEEERSTSDADPDKEL----------------------------------

P._patens_PIE1 QEDD----DDKEEKVVEGPTYVTPVEESKKGTSAAA-H----DIGSLGQP

H._sapiens_p400 PSSQ----------------------------------------------

S._cerevisiae_Eaf1p --------------------------------------------------

S._pombe_Vid21 --------------------------------------------------

P._patens_EAF1 --------------------------------------------------

S._moellendorfii_EAF1 --------------------------------------------------

B._distachyon_EAF1 --------------------------------------------------

A._thaliana_EAF1 --------------------------------------------------

A._thaliana_PIE1 EETEGNPSVRRSNDSYGHLAIS-ETHSHDLEPGMTTASVKSRKEDHTYDF

B._distachyon_PIE1 SSDEGNSS-EEVDDSYSYTEFVKKNH--GKSNGS-------------ISS

S._moellendorfii_PIE1 --------------------------------------------------

P._patens_PIE1 DSVEGCLVASETDENLAKLVVG-VTHRNT--IGAFLTQVKPQKRRHLSPK

H._sapiens_p400 --------------------------------------------------

S._cerevisiae_Eaf1p --------------------------------------------------

S._pombe_Vid21 --------------------------------------------------

P._patens_EAF1 --------------------------------------------------

S._moellendorfii_EAF1 --------------------------------------------------

B._distachyon_EAF1 --------------------------------------------------

A._thaliana_EAF1 --------------------------------------------------

A._thaliana_PIE1 NDEQEDVDFVLANGEEKDDEATLAVEEELAKADNEDHVEEIALLQKESEM

B._distachyon_PIE1 VGEQGDKDYVAAD-EGKDDEATLSEEEELAKGDSPDPLDEIKLLQKESEI

S._moellendorfii_PIE1 DDEEYDPHTAYA--SEEDDERTFDEEEKLAMAENKDVNLELEQLKMENEL

P._patens_PIE1 SLQHQIGELQVC--TADDDERTLEEEERIAMEEGDRNINEINELKLESEM

H._sapiens_p400 --------------------------------------------------

S._cerevisiae_Eaf1p --------------------------------------------------

S._pombe_Vid21 --------------------------------------------------

P._patens_EAF1 --------------------------------------------------

S._moellendorfii_EAF1 --------------------------------------------------

B._distachyon_EAF1 --------------------------------------------------

A._thaliana_EAF1 --------------------------------------------------

A._thaliana_PIE1 PIEVL-----------------------------------------LARY

B._distachyon_PIE1 PLEEL-----------------------------------------LARY

S._moellendorfii_PIE1 PVSEL-----------------------------------------LSRY

P._patens_PIE1 PLEEL-----------------------------------------LAKY

H._sapiens_p400 ----WPRPKPDGEDTSGEEDADDCPGDRESRKDLVLIDSLFIMDQFK---

S._cerevisiae_Eaf1p --------------------------------------------------

S._pombe_Vid21 --------------------------------------------------

P._patens_EAF1 --------------------------------------------------

S._moellendorfii_EAF1 --------------------------------------------------

B._distachyon_EAF1 --------------------------------------------------

A._thaliana_EAF1 --------------------------------------------------

A._thaliana_PIE1 KEDFGGKDISEDESES--SFAVSEDSIVDS---DENRQQADLDDDNVDLT

B._distachyon_PIE1 QMDGYADGVTTELENS--PTHYNEEVNTDM---SLDGQSVDILKLNNDML

S._moellendorfii_PIE1 RAEGAESSGDEKGGASDAPSDSSDEAKERMGEPSTSGRS-SLLIIKSTGR

P._patens_PIE1 RSGISGSSSDDEDDER--GVNYFSEAIERMGEPSTSGRD--YQRLSSTEV

H._sapiens_p400 --------------------------------------------------

S._cerevisiae_Eaf1p --------------------------------------------------

S._pombe_Vid21 --------------------------------------------------

P._patens_EAF1 --------------------------------------------------

S._moellendorfii_EAF1 --------------------------------------------------

B._distachyon_EAF1 --------------------------------------------------

A._thaliana_EAF1 --------------------------------------------------

A._thaliana_PIE1 E---------CK------LDPEPC-SENVEG------TF-----------

B._distachyon_PIE1 ENHEITDMLERKLVSGNALQPEIVPESSVQGCSVKEDEL-----------

S._moellendorfii_PIE1 S--K--ATGESKIFYGTEQEAEVLA--NVAR------AAQQRGERMRKRA

P._patens_PIE1 D--R--E-----MNNFNVI-----------N------TV-----------

H._sapiens_p400 -------------------------------------AA-----------

S._cerevisiae_Eaf1p --------------------------------------------------

S._pombe_Vid21 --------------------------------------------------

P._patens_EAF1 --------------------------------------------------

S._moellendorfii_EAF1 --------------------------------------------------

B._distachyon_EAF1 --------------------------------------------------

A._thaliana_EAF1 --------------------------------------------------

A._thaliana_PIE1 ----HEVAEDNDKDSSDKIADAAAAARSAQPTGFTYSTTKVRTKLPFLLK

B._distachyon_PIE1 ----TDAKVANEETGDSVIDDAAAAARSAQPTGNTFSTTSVRTKFPFLLK

S._moellendorfii_PIE1 GLDDSSRVEGSGKTGRKELDDAAAAALSAQPTGYTFSTTRVRTKIPFLLK

P._patens_PIE1 ----ETLDVGENKKSEDRLADYAAAAQSAQPTGYTFSTTQVKTKLPFLLK

H._sapiens_p400 ----E--RMNIGKPNAKDIADVTAVAEAILPKGSARVTTSVKFNAPSLLY

S._cerevisiae_Eaf1p --------------------------------------------------

S._pombe_Vid21 -----------------------------NAVFSDDIFVTT---------

P._patens_EAF1 ----------------------------ASGC-GRMAQA-VASVSH----

S._moellendorfii_EAF1 --------------------------------------------------

B._distachyon_EAF1 ----------------------------YNSQASDSVFTEAPPTPD----

A._thaliana_EAF1 ----------------------------YNNS-SISYHSAALSTPD----

A._thaliana_PIE1 HSLREYQHIGLDWLVTMYEKKLNGILADEMGL-GKTIMT-IALLAHLACD

B._distachyon_PIE1 HSLREYQHIGLDWLVAMYEKRLNGILADEMGL-GKTIMT-ISLLAHLACE

S._moellendorfii_PIE1 HSLREYQHIGLDWLVTMYEKRLNGILADEMGL-GKTIMT-IALLAHLACE

P._patens_PIE1 HSLREYQHIGLDWLVTMYEKRLNGILADEMGL-GKTIMT-IALLAHLACE

H._sapiens_p400 GALRDYQKIGLDWLAKLYRKNLNGILADEAGL-GKTVQI-IAFFAHLACN

S._cerevisiae_Eaf1p --------------------------------------------------

S._pombe_Vid21 --------------------------------------------------

P._patens_EAF1 --------------------------------------------------

S._moellendorfii_EAF1 --------------------------------------------------

B._distachyon_EAF1 --------------------------------------------------

A._thaliana_EAF1 --------------------------------------------------

A._thaliana_PIE1 KGIWGPHLIVVPTSVMLNWETEFLKWCPAFKILTYFGSAKERKLKRQGWM

B._distachyon_PIE1 KGIWGPHLIVVPTSVMLNWETEFLKWCPAFKILTYFGSAKERKQKRQGWM

S._moellendorfii_PIE1 KGIWGPHLIVVPTSVMLNWETEFMKWCPAFKVLTYFGNAKERKIKRQGWS

P._patens_PIE1 KGVWGPHLIVVPTSVMLNWETEFMKWCPAFKILTYFGNAKERKLKRQGWS

H._sapiens_p400 EGNWGPHLVVVRSCNILKWELELKRWCPGLKILSYIGSHRELKAKRQEWA

S._cerevisiae_Eaf1p --------------------------------------------------

S._pombe_Vid21 --------------------------------------------------

P._patens_EAF1 --------------------------------------------------

S._moellendorfii_EAF1 --------------------------------------------------

B._distachyon_EAF1 --------------------------------------------------

A._thaliana_EAF1 --------------------------------------------------

A._thaliana_PIE1 KLNSFHVCITTYRLVIQDSKMFKRKKWKYLILDEAHLIKNWKSQRWQTLL

B._distachyon_PIE1 KPNFFHVCITTYRLVIQDSKAFKRKKWKYLILDEAHLIKNWKSQRWQTLL

S._moellendorfii_PIE1 KANSFHVCITTYRLVIQDAKAFKRKKWKYLILDEAHLIKNWKSQRWQMLL

P._patens_PIE1 RPNSFHVCITTYRLVIQDAKAFKRKKWKYLILDEAHLIKNWKSQRWQTLL

H._sapiens_p400 EPNSFHVCITSYTQFFRGLTAFTRVRWKCLVIDEMQRVKGMTERHWEAVF

S._cerevisiae_Eaf1p --------------------------------------------------

S._pombe_Vid21 --NS----------------------------------------------

P._patens_EAF1 ---------------------------------K------LYWECN-GIL

S._moellendorfii_EAF1 -------------------------------------------VP--DSL

B._distachyon_EAF1 ---------------------------------R---------LNEFGIL

A._thaliana_EAF1 ---------------------------------N---------MCDPEIL

A._thaliana_PIE1 NFNSKRRILLTGTPLQNDLMELWSLMHFLMPHVFQSHQEFKDWFCN-PIA

B._distachyon_PIE1 NFNSKRRILLTGTPLQNDLMELWSLMHFLMPHVFQSHQEFKDWFCN-PIS

S._moellendorfii_PIE1 NFNSKRRILLTGTPLQNDLMELWSLMHFLMPHVFQSHQEFRDWFSN-PIS

P._patens_PIE1 NFNSKRRILLTGTPLQNDLMELWSLMHFLMPHVFQSHQEFRDWFCN-PIT

H._sapiens_p400 TLQSQQRLLLIDSPLHNTFLELWTMVHFLVPGIS------RPYLSS-PLR

S._cerevisiae_Eaf1p --------------------------------------------------

S._pombe_Vid21 --------------------------------------------------

P._patens_EAF1 -YFNDQERFSKEPL--YYNV-----------------PSGAA--EACRVS

S._moellendorfii_EAF1 -F--LMEQFPEESL--VFIV-----------------SDGAL--ETYRLS

B._distachyon_EAF1 -KV--STQLSQVSL--FYAV-----------------PPGAM--LAYRES

A._thaliana_EAF1 -DISMVDQLTEASL--FYSV-----------------PSGAM--EVYLKS

A._thaliana_PIE1 GMVEGQEKINKEVIDRLHNVLRPFLLRRLKRDVEKQLPSKHEHVIFCRLS

B._distachyon_PIE1 GMVEGQDKVNKEVIDRLHNVLRPFILRRLKRDVEKQLPQKHEHVIYCRLS

S._moellendorfii_PIE1 GMVEGQDKVNQDVVDRLHNVLRPFILRRLKRDVEKQLPGKHEHVVPCRLS

P._patens_PIE1 GMVEGEDQVNKELVDRLHNVLRPFLLRRLKKDVEKQLPGKFEHVIRCRLS

H._sapiens_p400 APSEESQDYYHKVVIRLHRVTQPFILRRTKRDVEKQLTKKYEHVLKCRLS

S._cerevisiae_Eaf1p --------------------------------------------------

S._pombe_Vid21 EQIQNCVLNVPM--------------------------------------

P._patens_EAF1 --------------------------------------------------

S._moellendorfii_EAF1 --------------------------------------------------

B._distachyon_EAF1 --------------------------------------------------

A._thaliana_EAF1 --------------------------------------------------

A._thaliana_PIE1 KRQRNLYEDFIASTETQATLTSGSFFGMISIIMQLRKVCNHPDLFEGRPI

B._distachyon_PIE1 RRQRNLYEDFIANSETQATLTSGNYFGMISIIMQLRKVCNHPDLFEGRPI

S._moellendorfii_PIE1 KRQRNLYEDFMASSDTQATLSGGNFLGLINVLMQLRKVCNHPDLFESRPI

P._patens_PIE1 KRQRNLYEDFMASSDTQATLSSGNFFGLINVLMQLRKVCNHPDLFEGRPI

H._sapiens_p400 NRQKALYEDVILQPGTQEALKSGHFVNVLSILVRLQRICNHPGLVEPRHP

S._cerevisiae_Eaf1p --------------------------------------------------

S._pombe_Vid21 --------------------------------------------------

P._patens_EAF1 --------------------------------------I--EMERTRAEV

S._moellendorfii_EAF1 --------------------------------------I--EKNWDAF-V

B._distachyon_EAF1 --------------------------------------V--ESLFMYHKN

A._thaliana_EAF1 --------------------------------------I--ESHLTRCEK

A._thaliana_PIE1 VSSFDMAGIDVQLSSTICSLLLESPFSKVDLEALGFLFTHLDFSMTSWEG

B._distachyon_PIE1 ISSFDMAGINMQISSSVCMVLDKGPFSQADLSDMNLVFTQNEFNMTSWEV

S._moellendorfii_PIE1 VSSFDMPGLQLQMCSELCTAVQQKPFSTVNLEVLNYLLS---EDMEAWEP

P._patens_PIE1 VSSFDMTGIKLHLSSAACSATAMGPFDGIDLGTLNLQFSRLSGTMTKWEA

H._sapiens_p400 GSSYVAGPLEYPSASLILKALERDFWKEADLSMFDLIGL--ENKITRHEA

S._cerevisiae_Eaf1p DGLL---SQEGAHDQIIS--------------------------------

S._pombe_Vid21 --------------------------------------------------

P._patens_EAF1 EYEAKR----------VVAVAEAEAPAAG-----EASGEVSL-----GID

S._moellendorfii_EAF1 SEKL---LQDSLNDQRLSGICS-ELDLYG-----DDSRS-----------

B._distachyon_EAF1 -------IGTGLKYNY----EASVCDSAA-----DLAQD-----------

A._thaliana_EAF1 -------SGSSMQEEV----DTSAYDTAG-----DIGYN-----------

A._thaliana_PIE1 DEIKAISTPSEL----IKQRVNLKDDLEAIPLSPKNRKNLQGTNIFE---

B._distachyon_PIE1 DEVADAFSPG------ITSRGSGAE----FSCSNKDGQRGIGKNIFE---

S._moellendorfii_PIE1 SELAELATPTPL----IEEIATSGEDSWN-----EQEQVTEPQNIFE---

P._patens_PIE1 HEVGMLKAPGPL----IVELAGTGEDTWDRH-QSKHKPSKEVRTVIQ---

H._sapiens_p400 ELLSKKKIPRKL----MEEISTSAAPAAR-----PAAAKLKASRLFQPVQ

S._cerevisiae_Eaf1p --------------------------------------------------

S._pombe_Vid21 --------------------------------------------------

P._patens_EAF1 FGFSDFLVDG----------------------------------------

S._moellendorfii_EAF1 --F-GSRTSP----------------------------------------

B._distachyon_EAF1 ---------N----------------------------------------

A._thaliana_EAF1 --V------T----------------------------------------

A._thaliana_PIE1 --------------------------------------------------

B._distachyon_PIE1 --------------------------------------------------

S._moellendorfii_PIE1 --------------------------------------------------

P._patens_PIE1 --------------------------------------------------

H._sapiens_p400 YG----QKPEGRTVAFPSTHPPRTAAPTTASAAPQGPLRGRPPIATFSAN

S._cerevisiae_Eaf1p --------------------------------------------------

S._pombe_Vid21 --------------------------------------------------

P._patens_EAF1 --------------------------------------------------

S._moellendorfii_EAF1 --------------------------------------------------

B._distachyon_EAF1 --------------------------------------------------

A._thaliana_EAF1 --------------------------------------------------

A._thaliana_PIE1 --------------------------------------------------

B._distachyon_PIE1 --------------------------------------------------

S._moellendorfii_PIE1 --------------------------------------------------

P._patens_PIE1 --------------------------------------------------

H._sapiens_p400 PEAKAAAAPFQTSQASASAPRHQPASASSTAASPAHPAKLRAQTTAQAST

S._cerevisiae_Eaf1p ---------------SI---------------------------------

S._pombe_Vid21 --------------------------------------------------

P._patens_EAF1 --------GSQEDEF--QTNYMG---------------------------

S._moellendorfii_EAF1 --------KLPDDDY--GLHYMP---------------------------

B._distachyon_EAF1 --------AYDEDEGETCTYLLAET-------------------------

A._thaliana_EAF1 --------AFDEDEGETSTYYLPGA-------------------------

A._thaliana_PIE1 --------------------------------------------------

B._distachyon_PIE1 --------------------------------------------------

S._moellendorfii_PIE1 --------------------------------------------------

P._patens_PIE1 --------------------------------------------------

H._sapiens_p400 PGQPPPQPQAPSHAA--GQSALPQRLVLPSQAQARLPSGEVVKIAQLASI

S._cerevisiae_Eaf1p ------------------------------DTKL----------------

S._pombe_Vid21 --------------------------------------------------

P._patens_EAF1 ------------------EGG-HARIYKKKRKKILK--------------

S._moellendorfii_EAF1 --------------------G-DLTISKKKRKRILK--------------

B._distachyon_EAF1 -----------------YDGGLLPKMGH-KKKHIMR--------------

A._thaliana_EAF1 -----------------FESSRSFNISHKKRKNLMK--------------

A._thaliana_PIE1 --------------------------------------------------

B._distachyon_PIE1 --------------------------------------------------

S._moellendorfii_PIE1 --------------------------------------------------

P._patens_PIE1 --------------------------------------------------

H._sapiens_p400 TGPQSRVAQPETPVTLQFQGS-KFTLSHSQLRQLTAGQPLQLQGSVLQIV

S._cerevisiae_Eaf1p -----------------------------------------LLKKPSSS-

S._pombe_Vid21 --------------------------------------------------

P._patens_EAF1 ---------SN--TGSKIEGIEL------QGLAYQSSDMSTPDRNVVTGK

S._moellendorfii_EAF1 ---------AQ--TPKAYDGLSQ------GGYCDGSNRLSSPAKRPTAG-

B._distachyon_EAF1 ---------QRINSARPYEIGTDVSYEPCLERKSGNQQFFSNGKRPSSF-

A._thaliana_EAF1 ---------SH--SARSYDLGDDLPY---VNNTGGSNSSSLIVKRPDSN-

A._thaliana_PIE1 --------------------------------------------------

B._distachyon_PIE1 --------------------------------------------------

S._moellendorfii_PIE1 --------------------------------------------------

P._patens_PIE1 --------------------------------------------------

H._sapiens_p400 SAPGQPYLRAP--GPVVMQTVSQ------AGAVHGALGSKPPAGGPSPA-

S._cerevisiae_Eaf1p ----------SEVVLIQH------------------------------EV

S._pombe_Vid21 --------------------------------------------------

P._patens_EAF1 RSLPGESGPNNAPGSIPVKRQRSSAINLRPRASHL--SISPGTT--GVLS

S._moellendorfii_EAF1 --------AASSIGVIPTKRMRTSAVVARQRAAST--TLSPGA----HRG

B._distachyon_EAF1 ----------V---GIPTKRIRTAA---RQRVVSPFPTSVGGT-P-HVKT

A._thaliana_EAF1 ----------INAGSVPTRRVRTAS---RHRVVSPFGCATTGNLPVPSKT

A._thaliana_PIE1 ----------------------------------------EIRK--AVFE

B._distachyon_PIE1 ----------------------------------------EIQK--ALQE

S._moellendorfii_PIE1 ----------------------------------------EIQA--ALAA

P._patens_PIE1 ----------------------------------------EIHS--SLRD

H._sapiens_p400 -------PLTPQVG-VPGRVAVNALAVGEPGTASK--PASPIGG--PTQE

S._cerevisiae_Eaf1p AASSA---------------------------------------------

S._pombe_Vid21 --------------------------------------------------

P._patens_EAF1 RTGPGSQNQQEGESFGMLDGSNRSD-SANDT----SNSKTHRIGDVGGFI

S._moellendorfii_EAF1 NASSASTNSQQEDLLGFP-DSRASE----------GDSVGPR---KGGLG

B._distachyon_EAF1 DASSGDTDSYQDDQSSLH-GGSLSWKNADYESTVDFDRQMPY---DGSEA

A._thaliana_EAF1 DASSGDTSSFQDEYSSLH-GGSAVQKGTEVESSVNFEKLLPY---DMAET

A._thaliana_PIE1 ERIQESKDRAAAIAWWNSLRCQRKP-TYSTS----LRTLLTI--------

B._distachyon_PIE1 ERMKEAKERAASIAWWNRIRCQKRP-VYGTN----IRELLTI--------

S._moellendorfii_PIE1 QRLKRRREKARQFGWLNMLRCSRHV-VYGRG----LAKHVEV--------

P._patens_PIE1 NRERQRRERLLAISVLNEFRCNQQP-LYGAD----FLKSAEI--------

H._sapiens_p400 EKTRLLKERLDQIYLVNERRCSQAP-VYGRD----LLRICALPSHGRVQW

S._cerevisiae_Eaf1p ----LIETEESKKELA-PPFK----------LSIFVDELNTFEKT-LIQD

S._pombe_Vid21 --------------------------------------------------

P._patens_EAF1 ----TNKW--NKKQN-PKTFPE-LSNSRSFEGGFPAGPP---EKSA-DED

S._moellendorfii_EAF1 ----TSSLRFKKKKK-TKAFH----------SDFSTDAI---SNSHLSKT

B._distachyon_EAF1 ----WT--KANKKKK-HKS--------------TGYKTA-----QNTA-N

A._thaliana_EAF1 ----SG--KPKKKKKTHQG--------------SAYDQT-----WHLN--

A._thaliana_PIE1 KGPLDDL-----KAN-CS-SYM-YSS-IL---------------------

B._distachyon_PIE1 RHPICDVL--EKKSN-PL-CHMEFSS-SL---------------------

S._moellendorfii_PIE1 EHPVYGIH--SIQSD-PS-RYLTFPS-AI---------------------

P._patens_PIE1 IHPVYDVH--KVNGN-PR-QYLEFSS-IL---------------------

H._sapiens_p400 RGSLDGRR--GKEAG-PAHSYT-SSSESP---------------------

S._cerevisiae_Eaf1p LPLYNGINE-ERPK-KD-DSL----------------------------P

S._pombe_Vid21 --------------------------------------------------

P._patens_EAF1 SHELLYHEQEQIKR-KG-ETNWSFVVTSSDASLAFCLKLLRIAGTPTDTP

S._moellendorfii_EAF1 DAELNQLEQDQLKR-KS-DQYSPL-------------------SSEHDTP

B._distachyon_EAF1 SYASAAVKKDYLKK-RS-ETQQF---------------------------

A._thaliana_EAF1 -PSVHVEQKDHWKK-RP-ENNF----------------------------

A._thaliana_PIE1 -ADIVLSPIERFQKMIELVEAFTFAIPAARVPSPTCW-------------

B._distachyon_PIE1 -ADLVLSSVERFNKMLGFIESFTFAIPAARAATPICW-------------

S._moellendorfii_PIE1 -AEVVKLPSSWCESLLDLLQAFVFVIPAARAPPPLIW-------------

P._patens_PIE1 -SDIVQLPLTRCESMIDLITAFVFAIPAARAPHPVAW-------------

H._sapiens_p400 -SELMLTLCRCGESLQDVIDRVAFVIPPVVAAPPSLR-------------

S._cerevisiae_Eaf1p FIPISKSV----VSLDDNGFYKLLE------------RQLID----E---

S._pombe_Vid21 --------------------------------------------------

P._patens_EAF1 ETPGSQSL----SGQQNVKKQKSS-------------KQSETD-AETATP

S._moellendorfii_EAF1 GTPGSQGV----VSMHPMKKLKSS-------------KHSPDF-AEAG-P

B._distachyon_EAF1 ---YSNGTIVTNGGQHAFKKLKMM-------------KQGIDI-SQEA--

A._thaliana_EAF1 ---DMNGL----YGPHSAKKQKTT-------------KQLVENNFDMAIP

A._thaliana_PIE1 ------------------------CSKSD-SPVFLSPSYKEKV-TDLLSP

B._distachyon_PIE1 ------------------------CKKRK-SPVLLEPAYREQC-MNEFSP

S._moellendorfii_PIE1 ------------------------CSRQS-ASSILRHDFPSNQ-LAVMSD

P._patens_PIE1 ------------------------CSHLIPASIDLKQSISEEV-LQRANS

H._sapiens_p400 -----------------VPRPPPLYSHR---MR----ILRQGL-REHAAP

S._cerevisiae_Eaf1p ------EPS-ISQLS-----------------------------------

S._pombe_Vid21 --------------------------------------------------

P._patens_EAF1 APAP-NPPT-LQQPPSLPSA------------------------------

S._moellendorfii_EAF1 --------S-ATG-LNLTTS------------------------------

B._distachyon_EAF1 ------SPV-ASQMSNMANP------------------------------

A._thaliana_EAF1 HTGSIPSPA-ASQMSNMSNP------------------------------

A._thaliana_PIE1 LLSP-IRPAIVRRQVYFPDRRLIQFDCGKLQELAMLLRKLKFGGHRALIF

B._distachyon_PIE1 ILSP-IRPAIVRRQVYFPDRRLIQFDCGKLQELAILLRRLKSEGHRALIF

S._moellendorfii_PIE1 LVAP-LRPVIVRQQLFFPDRRLLQFDCGKLQQLSVLLRRLKSEGHRALIF

P._patens_PIE1 MLVP-LRPVFVRKQLFFPDRRLLQFDCGKLQELAVLLRRLKSQGHRALIF

H._sapiens_p400 YFQQ-LRQTTAPRLLQFPELRLVQFDSGKLEALAILLQKLKSEGRRVLIL

S._cerevisiae_Eaf1p ----------------------------------------------KRRG

S._pombe_Vid21 --------------------------------------------------

P._patens_EAF1 ------------------------------------------NKLSKQSS

S._moellendorfii_EAF1 ------------------------------------------SKIMRQNS

B._distachyon_EAF1 ------------------------------------------AKIIKIIT

A._thaliana_EAF1 ------------------------------------------NKSIKFIG

A._thaliana_PIE1 TQMTKMLDVLEAFINLYGYTYMRLDGSTPPEERQTLMQRFNTNPKIFLFI

B._distachyon_PIE1 TQMTKMLDTLEEFINLYGYTYLRLDGSTQPEERQTLMQRFNTNPKYFLFI

S._moellendorfii_PIE1 TQMTKMLDILESFINLYGYNYMRLDGSTKPEQRQILMQRFNTNPKIFLFI

P._patens_PIE1 TQMTKMLDVLESFINLYGYTYMRLDGSTKPEQRQVLMQRFNTNPKIFLFI

H._sapiens_p400 SQMILMLDILEMFLNFHYLTYVRIDENASSEQRQELMRSFNRDRRIFCAI

S._cerevisiae_Eaf1p MF------------------------------------------------

S._pombe_Vid21 --------------------------------------------------

P._patens_EAF1 MRDRNRRLRLNKVPPSTPIGV-----------------------------

S._moellendorfii_EAF1 ARDRIRRNKATKG-------------------------------------

B._distachyon_EAF1 NRDRGRKVKALKM-------------------------------------

A._thaliana_EAF1 GRDRGRKIKGLKIS------------------------------------

A._thaliana_PIE1 LSTRSGGVGINLVGADTVIFYDSDWNPAMDQQAQDRCHRIGQTREVHIYR

B._distachyon_PIE1 LSTRSGGVGVNLVGADTVIFYDSDWNPAMDQQAQDRCHRIGQTREVNIYR

S._moellendorfii_PIE1 LSTRSGGVGINLVGADTVIFYDSDWNPAMDQQAQDRCHRIGQTREVHIYR

P._patens_PIE1 LSTRSGGVGINLVGADTVIFYDSDWNPAMDLQAQDRCHRIGQTREVHIYR

H._sapiens_p400 LSTHSRTTGINLVEADTVVFYDNDLNPVMDAKAQEWCDRIGRCKDIHIYR

S._cerevisiae_Eaf1p --------------------------------------------------

S._pombe_Vid21 --------------------------------------------------

P._patens_EAF1 --------------------------------------------------

S._moellendorfii_EAF1 --------------------------------------------------

B._distachyon_EAF1 --------------------------------------------------

A._thaliana_EAF1 --------------------------------------------------

A._thaliana_PIE1 LISESTIEENILKKANQKRVLDNLVIQNGEYNTEFFKKLDPMELFSGHKA

B._distachyon_PIE1 LISESTIEENILKKANQKRALDDLVIQRGSYNTEFFKKLDPMEFFSGHAP

S._moellendorfii_PIE1 LVSESTIEENILKKANQKRFLDNLVIQSGGYNTEFFKKLDPMELLSGVNT

P._patens_PIE1 LISESTIEENILKKANQKRILDDLVIQSGSYNTEFFKKLDPMELFSGLKE

H._sapiens_p400 LVSGNSIEEKLLKNGT-KDLIREVAAQGNDYSMAFLTQRTIQELFEVYSP

S._cerevisiae_Eaf1p --------------------------------------------------

S._pombe_Vid21 --------------------------------------------------

P._patens_EAF1 --------------------------------------------------

S._moellendorfii_EAF1 --------------------------------------------------

B._distachyon_EAF1 --------------------------------------------------

A._thaliana_EAF1 --------------------------------------------------

A._thaliana_PIE1 LTTKDE----KETSKHC-GADIPLSNADVEAALKQAEDEADYM-------

B._distachyon_PIE1 LNVEDQQKDRSMPSVVSNETGLALSNADVEAAIRQAEDEADYM-------

S._moellendorfii_PIE1 TKRADQ----------------PLSNADVDAALKSAEDEADYM-------

P._patens_PIE1 IKVRGTP-DKKLTSFIT-SSIKELSNAEVDAALKNAEDEADYM-------

H._sapiens_p400 MDDAGF----------------PVK-AEEFVVLSQEPSVTETIAPKIARP

S._cerevisiae_Eaf1p --------------------------------------------------

S._pombe_Vid21 YGPPT---------ENNEYCEEISE-------------------------

P._patens_EAF1 --------------------------------------------------

S._moellendorfii_EAF1 --------------------------------------------------

B._distachyon_EAF1 --------------------------------------------------

A._thaliana_EAF1 --------------------------------------------------

A._thaliana_PIE1 ---ALKRVEQEEAVDNQEFTEEPVER-PEDDELVNEDD-IKADEPAD-QG

B._distachyon_PIE1 ---ALKRLEQEEAADNQEFSEEAAGR-LEDDELVNEEA--KPDEHCSAEH

S._moellendorfii_PIE1 ---AMKKVELEEAAENQEFAEDV-S--VEEDECADDLE----DGKVSET-

P._patens_PIE1 ---AMKRVEQEEAAENQEFTEELFAGNVDEEDLADDLD----ESGKSRKL

H._sapiens_p400 FIEALKSIEYLEE-DAQKSAQEGVLG-PHTDALSSDSENMPCDEEPS---

S._cerevisiae_Eaf1p --------------------------------------------------

S._pombe_Vid21 --------------------------------------------------

P._patens_EAF1 --------------------------------------------------

S._moellendorfii_EAF1 --------------------------------------------------

B._distachyon_EAF1 --------------------------------------------------

A._thaliana_EAF1 --------------------------------------------------

A._thaliana_PIE1 -LVAAG--------PAKEE--MSLLHS-DIRDERAVITTSSQEDDTDVLD

B._distachyon_PIE1 KHQCSD---------VDNDK-SVALPV-NQLDEEKALRLAAGDGDMDMLA

S._moellendorfii_PIE1 ----------KVPSPEFETAGTPSLPE---LKEPQPL-LLDAEEEMDMLA

P._patens_PIE1 QTDIAGVDGSRVGERAPEDGGTAIITEVIPFGEFSIL-PADPDEEMDMLA

H._sapiens_p400 --------------------------------------------QLEE--

S._cerevisiae_Eaf1p --------------------------------------------------

S._pombe_Vid21 -------------------KYPITPVSRFAYA------------------

P._patens_EAF1 --------------------------------------------------

S._moellendorfii_EAF1 --------------------------------------------------

B._distachyon_EAF1 --------------------------------------------------

A._thaliana_EAF1 --------------------------------------------------

A._thaliana_PIE1 DVKQMAAAAADAGQAISSFENQLRPIDRYAIRFLELWDPIIVEAAME---

B._distachyon_PIE1 DVKQMAAAAAAAGQASSSFENQLRPIDRYAMRFLELWDPIIDKAAVN---

S._moellendorfii_PIE1 DVKQLAAAAAASGQG-NNFEDQLKPIERYAIQFLDLWNPIIDTSALE---

P._patens_PIE1 DVRQMAAAAAASGRGSISFEDQLRPVERYAMRFLELWDPRVDSMAVV---

H._sapiens_p400 ---------------LADFMEQLTPIEKYALNYLELFHTSIEQEKERNSE

S._cerevisiae_Eaf1p --------------------------------------------------

S._pombe_Vid21 --------------------------------------------------

P._patens_EAF1 --------------------------------------------------

S._moellendorfii_EAF1 --------------------------------------------------

B._distachyon_EAF1 --------------------------------------------------

A._thaliana_EAF1 --------------------------------------------------

A._thaliana_PIE1 NEAGFEEKEWELDHIEKYKEEMEAE--IDD-GEEPLVYEKWDADFATEAY

B._distachyon_PIE1 YQANVVEEEWELERIEKLKEDLEAE--IDE-DQEPLSYETWDVDFATTAY

S._moellendorfii_PIE1 TQVTYEEKEWELEQIEKLKEEQEAD--IDE-DDEPLLYESWDTSNADAAY

P._patens_PIE1 AQVSFEEKEWELDQLEKLKEEQEAE--MDE-DNEPLFYETWDTALADEAY

H._sapiens_p400 DAVMTAVRAWEFWNLKTLQE-REARLRLEQEEAELLTYTREDAYSMEYVY

S._cerevisiae_Eaf1p --------------------------------------------------

S._pombe_Vid21 --------------------------------------------------

P._patens_EAF1 --------------------------------------------------

S._moellendorfii_EAF1 --------------------------------------------------

B._distachyon_EAF1 --------------------------------------------------

A._thaliana_EAF1 --------------------------------------------------

A._thaliana_PIE1 RQQVEVLAQHQLMEDL------------------ENEARER-EAAEVAEM

B._distachyon_PIE1 RQHVEALAKKQLLEEQ------------------EKQACK--AAKELEE-

S._moellendorfii_PIE1 RQQVEVLTQQQELLQA------------------QWDAMHDEELQEADRL

P._patens_PIE1 RQQVDILAQQQVCKRA------------------LKLR-I-KALAEAAAT

H._sapiens_p400 -EDVDGQTEVMPLWTPPTPPQDDSDIYLDSVMCLMYEAT---PIPEAK--

S._cerevisiae_Eaf1p --------------------------------------------------

S._pombe_Vid21 -----------KTKLKSTCAKASRKRLFNQLELSPPE--SF-MEKKA---

P._patens_EAF1 --------------------------------------------------

S._moellendorfii_EAF1 --------------------------------------------------

B._distachyon_EAF1 --------------------------------------------------

A._thaliana_EAF1 --------------------------------------------------

A._thaliana_PIE1 VLTQNESAHVLKPKKKKKAKK----AKYKSLKKGSLAAESK------HVK

B._distachyon_PIE1 --TN-DIISHRKKSKKNKRKA----GKFKSLKRGRLSSESE------AML

S._moellendorfii_PIE1 I--EGE-------KQQKRPKK----KKLKSLERSSVEEYNFCSEEDAGAD

P._patens_PIE1 V--RGAENFTLKSKGKKKLKK----AKFKTLAEGSLMTGT----EDISIR

H._sapiens_p400 -LP---PVYVRK--------------------------------------

S._cerevisiae_Eaf1p --------------------------------------------------

S._pombe_Vid21 ---R----------------------------------------------

P._patens_EAF1 --------------------------------------------------

S._moellendorfii_EAF1 --------------------------------------------------

B._distachyon_EAF1 --------------------------------------------------

A._thaliana_EAF1 --------------------------------------------------

A._thaliana_PIE1 SVVKIEDST---DDDNEEFGYVSSSDSDMVTPLSRMHMKGKKRDLIVDTD

B._distachyon_PIE1 DETSVDTMS---IDGNAP-------SPELISDESPHHCSHKRKKMVSRNE

S._moellendorfii_PIE1 DHIDADEADEVLSIASDEGEYDMQWDN----SLR--TPFQRKRKFSRFFE

P._patens_PIE1 EEFQVDRSS-------DPSYRDQHSDM-MLLPHR--SLSQRKRKAPMLLK

H._sapiens_p400 ---E--------------------------------------RKR-HKTD

S._cerevisiae_Eaf1p ---------------------------------YGNR-------------

S._pombe_Vid21 -----------SDEN----------------QLDGNK-------IKDDN-

P._patens_EAF1 --------------------------------------------------

S._moellendorfii_EAF1 --------------------------------------------------

B._distachyon_EAF1 --------------------------------------------------

A._thaliana_EAF1 --------------------------------------------------

A._thaliana_PIE1 EEKTSKKKAKKH-KKSLPNSDIKYKQTS-ALLDELEPSKPSDSMVVDNE-

B._distachyon_PIE1 EVNSSSRSLKKF-KKA-PKSNCISESSSHKHLLEGKQLKLMDE-VNFSD-

S._moellendorfii_PIE1 EEGGENKRFRKEDSRTEY--------------------------------

P._patens_PIE1 EEVRVEAHTKKL-KKNHFGKDR-RSSGSTLQESVDQPGGLSNS-GVHGG-

H._sapiens_p400 PSAAGRKKKQRHGEA-----------------------------VVPPRS

S._cerevisiae_Eaf1p -------RNHYLR--------------------------P-PAVP-----

S._pombe_Vid21 -QKLSSVGTFSVR--------------------------P-PYPP-----

P._patens_EAF1 --------------------------------------------------

S._moellendorfii_EAF1 -------------------------------------FIQ----S-----

B._distachyon_EAF1 --------------------------------------------------

A._thaliana_EAF1 --------------------------------------------------

A._thaliana_PIE1 -LKLTN------R--GKTVGKK-----------------FITSMPIKRVL

B._distachyon_PIE1 -PKLVS---------IRSDGRI-----------------STPCMPVKRVM

S._moellendorfii_PIE1 ----F-------R--PRFGGKL-----------------TIC-TTARKSP

P._patens_PIE1 -TVLA-------G--KDKRGKL-----------------TILGMPPKKGP

H._sapiens_p400 LFDRATPGLLKIRREGKEQKKNILLKQQVPFAKPLPTFAK----P-----

S._cerevisiae_Eaf1p ----------SLRYL-QN-RTPTIWLSEDDQELVKNINTY----------

S._pombe_Vid21 ----------SSK---DI-RPEAPWLPEEDELLLLLLRRY----------

P._patens_EAF1 ---------------------GTPWSASEDQAILALVHDL----------

S._moellendorfii_EAF1 ----------SIN---PP-GVGIPWSATEDQAILALVHDL----------

B._distachyon_EAF1 -------------------SSGGGWSNFEDQALVVLVHDL----------

A._thaliana_EAF1 ----------PG----QH-GSGNPWSLFEDQALVVLVHDM----------

A._thaliana_PIE1 MIKPEKLK-KGNLWSRDCVPSPDSWLPQEDAILCAMVHEY----------

B._distachyon_PIE1 VIKPERLKRKGLIWPRDCV--PDSWTNEEDAVLCGTVHEY----------

S._moellendorfii_PIE1 VILLERDRRKEALKAKDHH---TDWMPEEDEVLCAVVHEY----------

P._patens_PIE1 LIMLEKERKKDSLRSQDHLPPASPWTHGEDAVLCAVVHEY----------

H._sapiens_p400 ----------TAE---PG-QDNPEWLISEDWALLQAVKQLLELPLNLTIV

* :* : :.

S._cerevisiae_Eaf1p ----GYNWELISAHMTHRLTYSYLSNIERRTPWQCFERFVQLNE-RFNFS

S._pombe_Vid21 ----SFNWEFVASRLTPPGLY--IPLAEKRTAWDCFERWIQVDP-RAANV

P._patens_EAF1 ----GPNWELVSDVLSSNSQI----KGIYRRPNQCKERHKSLTE-RSNLD

S._moellendorfii_EAF1 ----GPNWELVSDVLSSSSQL----KGIFRKPKDCKDRHKSLLD-RLSND

B._distachyon_EAF1 ----GQNWELVSDAINNIVQF----KSVHRQPKECKERHKVLVD-KSSGD

A._thaliana_EAF1 ----GPNWELISDAMNSTLKI----KYIYRNPTECKDRHKILMD-KTAGD

A._thaliana_PIE1 ----GPNWNFVSGTLYGMTA-GGAYRGRYRHPAYCCERYRELIQ-RHILS

B._distachyon_PIE1 ----GPVWELASEFLHSIPG-GAFYRGRYRHPVHCCERFRELIC-KHVLS

S._moellendorfii_PIE1 ----GGNWLLASDALEGMPD-GGVYRGRHRHPVKCKDRFRQLVV-ENAGT

P._patens_PIE1 ----GGNWQLASDALAGGPD-GGVYRGRHRHPVYCRERFRELLA-QNAAA

H._sapiens_p400 SPAHTPNWDLVSDVVNSCSR-------IYRSSKQCRNRYENVIIPREEGK

* : : : * . * :* : .

S._cerevisiae_Eaf1p DLKGPRAHSA-----QQWLI------------------------------

S._pombe_Vid21 QLTGSHAR-----LAQQKLDESLRHSDKVSQHLSLRD-EGTPNH--LIKH

P._patens_EAF1 GMESPEDSNSS-QLQS-----LKGSVHGG-----TRV-HGTPEEDSLK-H

S._moellendorfii_EAF1 NGDNDEPGTSM-P-------------------------KGPMEEDTVKVH

B._distachyon_EAF1 GADSAEDSGSS-QHYQF---TIPGIPKGSARQLFQRL-QGPFEEENLKSH

A._thaliana_EAF1 GADSAEDSGNS-QSYPS---TLPGIPKGSARQLFQRL-QGPMEEDTLKSH

A._thaliana_PIE1 ASDSAVNEKNLNTGSGK---ALLKVTEENIRTLLNVAAEQPDTEMLLQKH

B._distachyon_PIE1 AMDNTNSEK-VPSGTGK---AILKVSEDQTQMLLNAISEIPNNELLLQKH

S._moellendorfii_PIE1 ICGGVSSER-LLSG------AVIKVTEEDTKRLLELVQRVPDKEVLLQRH

P._patens_PIE1 ASGDPVSERSALSAATN---AQLKVTEEHTKRLLNAVLQLPDKELLLQRH

H._sapiens_p400 -SKNNRPLRTS-QIY-------------------AQD-ENATHTQLYTSH

.

S._cerevisiae_Eaf1p ---------EAHKFQQRQNRR-----------------------------

S._pombe_Vid21 NSYFLLP-------------------------------------------

P._patens_EAF1 LE-CIVQLVLKYRARKSSSENEDQKTSAAQHPSHGIAISQFC----AGGP

S._moellendorfii_EAF1 LE-RIVNIVQAHRSKQQQNDTLENQELLPVHASHLSTASQFS---LSGVP

B._distachyon_EAF1 FE-KIALLMPQVQSRRRQVNSRELKPIIQPHSSHVAALSQACPNNLSGST

A._thaliana_EAF1 FE-KICLIGKKLHYRKTQNDGRDPKQIVPVHNSQVMALSQVFPNNLNGGV

A._thaliana_PIE1 FS-CLLSSI--WRTSTRT--GND--QM--------LS-------------

B._distachyon_PIE1 FM-AILSSV--WRSKC----GHEPRRVTSTCS---SA-------------

S._moellendorfii_PIE1 FA-TVQSVKDSYKGSAKS--RNG--FV--------SN-------------

P._patens_PIE1 FV-AALAAVEKW--------------------------------------

H._sapiens_p400 FD-LMKMTAGKRSPPIKPLLGMNPFQKNPKHAS-VLAESGIN----YDKP

S._cerevisiae_Eaf1p ISPLG---------------------------------------------

S._pombe_Vid21 --------------------------------------------------

P._patens_EAF1 LNPLELCDRVANNGDANSHSYPIQPVQGSGLG-PSLMPGSAMRPPSAGLP

S._moellendorfii_EAF1 LSPLDLCEHPMSNGDIPAHGY--SLPQHPGLNMPGVMAPNGMRPNSAGLP

B._distachyon_EAF1 LMPLDLCDTISPNLDAITPGSGYQGSHANGLTL-SNHHGSIGTPSPTPN-

A._thaliana_EAF1 LTPLDVCDASTSGQDVFS-------LENPGLPM-LN-QGTPVLPTSGAH-

A._thaliana_PIE1 --------------------------------------------------

B._distachyon_PIE1 --------------------------------------------------

S._moellendorfii_PIE1 --------------------------------------------------

P._patens_PIE1 --------------------------------------------------

H._sapiens_p400 LPPIQV--------------------------------------------

S._cerevisiae_Eaf1p --------------------------------------------------

S._pombe_Vid21 --------------------------------------------------

P._patens_EAF1 SVLPGSAGLHLGQSPVSSSAAMSAAAARDAQRFTT--R-LSPGEATRLRM

S._moellendorfii_EAF1 L-LP-----HLGSMVSPGSAAINAAAARDAQRMAA--S-MRPLSAEEQRL

B._distachyon_EAF1 SRLQGSPGMVLGSNL-SSPATLS-APSRDSQKYGAPRSTSLQVDDEQQKI

A._thaliana_EAF1 PSTPGSSGVVLSNNL-PTTSGLQSASVRDGR-FNVPRG-SLPLDEQHRLQ

A._thaliana_PIE1 ---------------------LN-SPIFNRQFM-----------------

B._distachyon_PIE1 ---------------------LN-KPVRLNEK------------------

S._moellendorfii_PIE1 ---------------------VG-HP-RNLPFV-----------------

P._patens_PIE1 --------------------------------------------------

H._sapiens_p400 ------------------------ASLR-AERIAK--E-KKAL-ADQQKA

S._cerevisiae_Eaf1p ----------------------VNT-------------------------

S._pombe_Vid21 --------------------------------------------------

P._patens_EAF1 AANNMSAYNARRLQQQAASVTAGLPILTGL--------------------

S._moellendorfii_EAF1 RYTRM--VTGRSLQPQA-NSPGNLPM-ASL--------------------

B._distachyon_EAF1 QYNQV--ASGKNPQQAGVSAPGTCP-------------------------

A._thaliana_EAF1 QFNQT--LSGRNLQQPSLSTPAAV--------------------------

A._thaliana_PIE1 --------------------------------------------------

B._distachyon_PIE1 --------------------------------------------------

S._moellendorfii_PIE1 --------------------------------------------------

P._patens_PIE1 --------------------------------------------------

H._sapiens_p400 QQPAV--AQPPPPQPQ-PPPPPQQPP-PPLPQPQAAGSQPPAGPPAVQPQ

S._cerevisiae_Eaf1p ---ES------IQRGH---RRL-RWAS-----------------------

S._pombe_Vid21 --------------------------------------------------

P._patens_EAF1 --PNP--------NDL---PML-PTSTGAMM-GALSRG-LSMPRPGLSSM

S._moellendorfii_EAF1 --PNS------SDCSM---PML-PSGNTAGL-MGLNRGGLSLPRPTMPGM

B._distachyon_EAF1 ---AG------VDRGA---HVM-PAVNGIGMTAGVNRG-P----------

A._thaliana_EAF1 ---SG------SDRGH---RMV-PGGNAMGV-SGMNRN-TPMSRPGFQGM

A._thaliana_PIE1 --------------------------------GSVNHT-QDLARKPWQGM

B._distachyon_PIE1 ----------------------------------------------WSMT

S._moellendorfii_PIE1 --------------------------------SLANLC-QGCLKPPLRQA

P._patens_PIE1 --------------------------------------------------

H._sapiens_p400 PQPQPQTQPQPVQAPAKAQPAITTGGSAAVL-AG----------------

S._cerevisiae_Eaf1p --------------------------------------------------

S._pombe_Vid21 --------------------------------------------------

P._patens_EAF1 GSPVISNIVPAGLGGMVPPPGTLSSPSMSRRTANLLNMLRVIHVCGVPGN

S._moellendorfii_EAF1 IPPG-PS-------PGLMPPGGVALPASGSM-----------MAGGIPSP

B._distachyon_EAF1 --------------------SVQGVPNAVNV-----------HSGAMSSL

A._thaliana_EAF1 ASAAMPNTGNMH------TSGMVGIPNTGNI-----------HSGGGASQ

A._thaliana_PIE1 KVT-----------------------------------------------

B._distachyon_PIE1 N-Y-----------------------------------------------

S._moellendorfii_PIE1 SH------------------------------------------------

P._patens_PIE1 --------------------------------------------------

H._sapiens_p400 -------TIKTSVTGTSMPTGAVSGNVIVNT------------IAGVPAA

S._cerevisiae_Eaf1p ----------------M------------------------------FEA

S._pombe_Vid21 ------TVSRHYRPITI------------------------------FEA

P._patens_EAF1 LEVWCKYYL-CIKEHRLFQVGGCTED--------------------QRVL

S._moellendorfii_EAF1 AN----FMKRAREATQLIRSGRCTEE--------------------QKQH

B._distachyon_EAF1 GN----SVLRPRDPMQ----------------------------------

A._thaliana_EAF1 GN----SMIRPREAVQ----------------------------------

A._thaliana_PIE1 --------------------------------------------------

B._distachyon_PIE1 --------------------------------------------------

S._moellendorfii_PIE1 --------------------------------------------------

P._patens_PIE1 --------------------------------------------------

H._sapiens_p400 T-----FQSINKRLASPVAPGALTTPGGSAPAQVVHTQPPPRAVGSPATA

S._cerevisiae_Eaf1p IRKCMKK-------------------------------------RENTPR

S._pombe_Vid21 IRKILKK----------R---------------------------EFAKK

P._patens_EAF1 ILQQLQLLAQQGDNQAAAAFSNLGGDMATMIPSSPQVCKSTKVEGKFAGP

S._moellendorfii_EAF1 IIQELQHQAAQGNPQAVAALNTLSSNNMN---------------------

B._distachyon_EAF1 --IRM---VSQGSSRA-AHFSSMNPPFSK---------------------

A._thaliana_EAF1 --HMMRMQAAQGNSPGIPAFSNLSSGFTN---------------------

A._thaliana_PIE1 --------------------------------------------------

B._distachyon_PIE1 --------------------------------------------------

S._moellendorfii_PIE1 --------------------------------------------------

P._patens_PIE1 --------------------------------------------------

H._sapiens_p400 TPDLVSMATT----QGVRAVTSVTA-------------------------

S._cerevisiae_Eaf1p PNPTQ------------------------PRKPLDCKNMKVP-TP-----

S._pombe_Vid21 PTMTKR--------------------AIAPSAASTE--------------

P._patens_EAF1 PTVTLRPDEAIIILPSTSFDSVATASTAARSAAATTAAPPAT-SSTTTSS

S._moellendorfii_EAF1 ------------------------------------LSNPMVSSPTQ-SF

B._distachyon_EAF1 ------------------------------------AAAPS---PVH--Q

A._thaliana_EAF1 ------------------------------------QTTPVQAYPGH--L

A._thaliana_PIE1 -------------------------------------------SLSR-KL

B._distachyon_PIE1 -------------------------------------------RPTS-NL

S._moellendorfii_PIE1 -------------------------------------------KQGH-AL

P._patens_PIE1 --------------------------------------------------

H._sapiens_p400 ------------------------------SAVVTTNLTPVQ-TPAR-SL

S._cerevisiae_Eaf1p ------------------------AEMSLLKAQRDEALR------RDI--

S._pombe_Vid21 --------------------------------------------------

P._patens_EAF1 ATTAT-AASSK----SATKSTVTNLLFACLSCEQQQMQLQGNPTQAYYSA

S._moellendorfii_EAF1 APQQQ-Q---------------------HLSPQQQSQQQ------QHQQY

B._distachyon_EAF1 T--QKLH--QM----SQPSHVLGNPHNPRAQGT--NH------SSSHQQS

A._thaliana_EAF1 S--QQ-H--QM----SPQSHVLGNSHHPHLQSP--SQ------ATGAQQ-

A._thaliana_PIE1 LESAL-QDSGP----SQPDNTISRS-------------------------

B._distachyon_PIE1 IKTAL-ADAQA----QCPRAVLPR--------------------------

S._moellendorfii_PIE1 VAEAL-SQTTASDNGKMPENSACKA-------------------------

P._patens_PIE1 --------------------------------------------------

H._sapiens_p400 VPQVS-QATGV----QLPGKTITPAHFQLLRQQQQQQQQ------QQ---

S._cerevisiae_Eaf1p --------QLRRTVKNRLQQRQQQSQ------------------------

S._pombe_Vid21 --------------------------------------------------

P._patens_EAF1 AAAV---RMKEQQYRQQHQQKQRLMGTLPPTQAQYMLVSPHVQNLPAQQL

S._moellendorfii_EAF1 LAAVRLVNERQQQQQQQQQQQQKR--------------------------

B._distachyon_EAF1 YAGHF---AKERQFPQRMVPQQHN-D--PSGAS----AVPSVQN------

A._thaliana_EAF1 -EAFA---IRQRQIHQRYLQQQQQQF--PASGS----MMP----------

A._thaliana_PIE1 ----------------RLQETQPIN-------------------------

B._distachyon_PIE1 --------------------NQESG-------------------------

S._moellendorfii_PIE1 ----------------VKNNTASDN-------------------------

P._patens_PIE1 --------------------------------------------------

H._sapiens_p400 -------QQQQQQQQQQQQQQQQQQQ------------------------

S._cerevisiae_Eaf1p -------------QAHSSRAQS-PIP---------------SNGKSS---

S._pombe_Vid21 --------------------------------------------------

P._patens_EAF1 GSKSHMLSQQPQRTHLGQQHQGSGLSSQSSQPALSTLNLQSSSASPTATG

S._moellendorfii_EAF1 ------IFQQP--NNLLQQHQL-GMLTQQQQPLLP---------------

B._distachyon_EAF1 -----------------------GP------------HIQQQNKAPVA--

A._thaliana_EAF1 -------------------------------------HVQQPQGSSVS--

A._thaliana_PIE1 --------------------------------------------------

B._distachyon_PIE1 --------------------------------------------------

S._moellendorfii_PIE1 --------------------------------------------------

P._patens_PIE1 --------------------------------------------------

H._sapiens_p400 -------------TTTTSQVQV-PQIQGQAQSPAQIK-------------

S._cerevisiae_Eaf1p -----------------SNLA---R-NG----------------------

S._pombe_Vid21 ---------------KLPPV---------------------PSPLELSRL

P._patens_EAF1 QSNSSAQQMPLPQQ-NQSVVQP-GEATG------------SAKPPQ----

S._moellendorfii_EAF1 -SQQAQQLSPSHQQ-QQSQVLPGAQHPQ------------QPTPTQMGQL

B._distachyon_EAF1 --SSTPPSQPLHQQ-QQPPI--------------------QNPPDSFAPP

A._thaliana_EAF1 --SSSQ----NSPQ-TQPPV--------------------SPQPLSMPPV

A._thaliana_PIE1 -----------------KLGL---ELTLEFPRGNDDSLNQFPPMISLSID

B._distachyon_PIE1 -----------------RNYL---ELVLDFRTDQHAYEADFPSVVNVSIL

S._moellendorfii_PIE1 ---NDN----GDVE-S-QPPL---ELSLSFSD-PE-EMEDFRP-VNVTLR

P._patens_PIE1 --------------------------------------------------

H._sapiens_p400 ---AVGKLTPEHLIKMQKQKL---QMPP------------QPPPPQ----

S._cerevisiae_Eaf1p ------QASAPRPNQK-------------------------Q-YTEQD--

S._pombe_Vid21 ----------------------------------------KSEREAQIQ-

P._patens_EAF1 -G----KQPQKYPTQT-------------------------S--------

S._moellendorfii_EAF1 SG----QQKVQVPTQK-------------------------QSLTKQPSF

B._distachyon_EAF1 N-QPAS--TAE-PKQK------------------------KQQGQQQVR-

A._thaliana_EAF1 SPSPNINAMAQ-QKPQKSQLALHGLGRSPQSGTSGVNNQAGKQRQRQLQQ

A._thaliana_PIE1 GSDSLN---------Y--------------------------VN------

B._distachyon_PIE1 EPEPVK---------R--------------------------AI------

S._moellendorfii_PIE1 PGEPKT-------------------------------------A------

P._patens_PIE1 --------------------------------------------------

H._sapiens_p400 -A----QSAPPQPTAQ----------------------------------

S._cerevisiae_Eaf1p --------------------------------------------------

S._pombe_Vid21 ---QIQAQR--NFAQLQS--------------------------------

P._patens_EAF1 QIMQHH------QQHQQHTVRNSKGPMRGAIMQNLPSQTGQQSPNNLSGG

S._moellendorfii_EAF1 QQQQLL------QVQQQSKVGKNNASGRGVATHSALMPGSQ-QTANS-QL

B._distachyon_EAF1 ---QNQQQR--NQASQQAKLMKSL--GRG---------------------

A._thaliana_EAF1 SARQHPHQRQPTQGQQLNKQLKGM--GRG---------------------

A._thaliana_PIE1 --------------------------------------------------

B._distachyon_PIE1 --------------------------------------------------

S._moellendorfii_PIE1 --------------------------------------------------

P._patens_PIE1 --------------------------------------------------

H._sapiens_p400 -VQ-----------------------------------------------

S._cerevisiae_Eaf1p ------------------------II------------------------

S._pombe_Vid21 --------------------------------------------------

P._patens_EAF1 SNQPLNETPGHMLIQPQLGQVQAGKASSGSHPSG-SSQSHG-SLA----Q

S._moellendorfii_EAF1 PSPPGT-AQAGQAQQRSLAMQQAGKQASMSQPGKVSGQSQGGQLT----Q

B._distachyon_EAF1 ----------NMLI-PQTPAVDAAPASAVSTCSK-KQASDK-NLM----Q

A._thaliana_EAF1 ----------NMI--HQNITVDQSHLNGLTMPQG-NQATEK-GEIAVSVR

A._thaliana_PIE1 ------------------EPPGEDVLKGS---------------------

B._distachyon_PIE1 ------------------VQVDQSLLSGL---------------------

S._moellendorfii_PIE1 ------------------EPPSSSSIR-----------------------

P._patens_PIE1 --------------------------------------------------

H._sapiens_p400 --------------------------------------------------

S._cerevisiae_Eaf1p ----------------------------ESYSR-----------KLLEQK

S._pombe_Vid21 --------------------------------------------------

P._patens_EAF1 GGTPSC----------SGVSQIKGLHQSQTQ-P-----------------

S._moellendorfii_EAF1 G-TA------------GQTSQQKSL---QKQSP-----------QGASPV

B._distachyon_EAF1 HGQGSSPGNKASTSAIPQPGNQHML---YASLP-----------QSPKQL

A._thaliana_EAF1 PDQQSSVGTTTSTDLQSKPFVSPLS---SNHSQQLPKSFPGALSPSPQQQ

A._thaliana_PIE1 --------------------------------R-----------VAAENR

B._distachyon_PIE1 -------------------------------SH-----------RNAEKR

S._moellendorfii_PIE1 --------------------------------S-----------LVCETR

P._patens_PIE1 --------------------------------------------------

H._sapiens_p400 ---------------------------VQTS-------------------

S._cerevisiae_Eaf1p PDIGPEM--ALKAAKNY-------------------------YR------

S._pombe_Vid21 --------------------------------------------------

P._patens_EAF1 -QQGGV-PDQQQGSSIQ------IATQVSL-PQS----GSLPTSSEQSQE

S._moellendorfii_EAF1 PAAPSTPPPQQQTTP---------ATSAAA-PPA----SQQPASTTTQ-A

B._distachyon_EAF1 PDTSSQG--LMQGSPSH------TLLAAPQ-PPV----HSKPPSTTQQ-R

A._thaliana_EAF1 MQLHSDN--SIQGQSSPATPCNILSTSSLSIAPA-VAPSNHQHLLIHQ-K

A._thaliana_PIE1 YRNAANA--CIE-DSF------GWASNTFPANDLKSRT------------

B._distachyon_PIE1 FRIASEA--CFDGEGS------HWASSAFHVYDA-ARH------------

S._moellendorfii_PIE1 FRLASQM------TPS------IWAAAAISVVPK-PTP------------

P._patens_PIE1 --------------------------------------------------

H._sapiens_p400 -----Q-PPQQQSP--Q------L-TTVTA-PRP----GALLTGTTVANL

S._cerevisiae_Eaf1p ---TLR-EQQQQLKQHQ-IQQ--QRQQLQE--------------------

S._pombe_Vid21 --------------------------------------------------

P._patens_EAF1 PLATPSAQQTQQMQRRQMPQPQQPQRRLQSRHLSATPLVMPMPGQ-LGKT

S._moellendorfii_EAF1 QIQ-RRQQQQQQQQQQQQPQSQQAPQARKSQQKQASALQA--PGKNT---

B._distachyon_EAF1 QIN-PSQNSIQRMMMQQNLQMNS---------------------------

A._thaliana_EAF1 QRN-QVQSTAQRVVQH-NHLGNS---------------------------

A._thaliana_PIE1 -----G-TKAQSLGKHK-LSA--S---------DSAKSTK--SKH-----

B._distachyon_PIE1 -----K-SGPKSVGKHK-TSS------------ESGRPAK--SKI-----

S._moellendorfii_PIE1 -----S-QNNPPVKRQQ-PPT------------DVAKLQR--PGK-----

P._patens_PIE1 --------------------------------------------------

H._sapiens_p400 QVARLTRVPTSQLQAQGQMQTQAPQPA---QVALAKPPVVSVPAAVVSSP

S._cerevisiae_Eaf1p --------------------------------------------------

S._pombe_Vid21 --------------------------------------------------

P._patens_EAF1 GMQQTNQDVSP-QMVNHNPYQLGGTAMIGNPMSMSSPNSHVVSLPSTLN-

S._moellendorfii_EAF1 -TQPGLQ-------------PLGGSNSGSGPMSIGTSSSGLPP-QAALA-

B._distachyon_EAF1 ---DCRMDAQI-DQIQHNP-----------------------VIP-----

A._thaliana_EAF1 ---ELSKKSQA-ECM---P-----------------------RVP-----

A._thaliana_PIE1 --RKLLA-------------------------------------------

B._distachyon_PIE1 --QRTT--------------------------------------------

S._moellendorfii_PIE1 --QPRLQ-------------PV------------SKPNGMIEIRPVEMK-

P._patens_PIE1 --------------------------------------------------

H._sapiens_p400 GVTTLPMNVAGISVAIGQPQKAAGQTVVAQPVHM--------------QQ

S._cerevisiae_Eaf1p ---------------------------------------------ES---

S._pombe_Vid21 ----------QNRALR----------------------------PQNAAV

P._patens_EAF1 ------------SSTIGAHSTQWKSSQSNNPLTGGLYNLTRANSP-----

S._moellendorfii_EAF1 ------------PSAAG----SWKPGQ--NF--PGMYNLTRSTSG-----

B._distachyon_EAF1 --------------------------------------------------

A._thaliana_EAF1 --------------------------------------------------

A._thaliana_PIE1 --------------------------------------------------

B._distachyon_PIE1 --------------------------------------------------

S._moellendorfii_PIE1 ------------TS------------------------------------

P._patens_PIE1 --------------------------------------------------

H._sapiens_p400 LLKLKQQAVQQQKAIQ----------------------------PQAAQG

S._cerevisiae_Eaf1p ---SH---------------------------------------------

S._pombe_Vid21 A-------------------------------------------------

P._patens_EAF1 -GS--SQVSTMVISGPQLPTFAGTAA---VPTLSTSGIHGVPTGSVPQNL

S._moellendorfii_EAF1 -GA--NQ------------------------------------GSATTNG

B._distachyon_EAF1 --------------------------------------------------

A._thaliana_EAF1 --------------------------------------------------

A._thaliana_PIE1 ----------EQLEGAWV-------RPN-DPNLKFDF---TPG-------

B._distachyon_PIE1 -------------------------EPQDVPTAMNDFLR-APG-------

S._moellendorfii_PIE1 -------VSPSQIKPANL-------KKP-PPTAVFTT---TGA-------

P._patens_PIE1 --------------------------------------------------

H._sapiens_p400 PAAVQQKITAQQITTPGAQQKVAYAA---QPALKTQFLT-TPI-------

S._cerevisiae_Eaf1p --------------------------------------------------

S._pombe_Vid21 --------------------------AGAQQ-------------------

P._patens_EAF1 AGKQCLSNIVLNQKIGVNPGVNGRVLSGAQQPTLSGQR----SQQNGSVV

S._moellendorfii_EAF1 NGGAALNQVGTAAKLGIVPAVNGRTLAQQQQGSGAAQRPVQQQQQSLPVP

B._distachyon_EAF1 --------------------------------------------------

A._thaliana_EAF1 --------------------------------------------------

A._thaliana_PIE1 --------------------D-----------------------------

B._distachyon_PIE1 --------------------Q-----------------------------

S._moellendorfii_PIE1 --------------------SRVHHVAT--------KP-----SSSSSIQ

P._patens_PIE1 --------------------------------------------------

H._sapiens_p400 --------------------SQAQKLAGAQQ------------VQTQIQV

S._cerevisiae_Eaf1p ------------VQQLQQLQPGSQAPPPK---------------------

S._pombe_Vid21 -----HNQQLAA---FQAVA-ASQNSSNN--SSAGVSP------------

P._patens_EAF1 ISLPSGNSQMTA----PGSS-ASTGGSPT--SGAGVPP------------

S._moellendorfii_EAF1 VPVPTNTVNSTRPAR-----PSSVLPSSPSFPGTPVPSLSHGVQPRQVHG

B._distachyon_EAF1 ---------------TTSISHGTESSSP----------------------

A._thaliana_EAF1 ---------------Q-SVTNTTQTASM-------------GT-------

A._thaliana_PIE1 --------------------------------------------------

B._distachyon_PIE1 --------------------------------------------------

S._moellendorfii_PIE1 LARPSSSAKSTAVA----AA------------------------------

P._patens_PIE1 --------------------------------------------------

H._sapiens_p400 AKLPQVVQQQTPVASIQQVASASQQASPQ--TVALTQA------------

S._cerevisiae_Eaf1p -----------SSPS-----------------------------------

S._pombe_Vid21 -------------------IAGRM---V----------------------

P._patens_EAF1 ------------------QGEGLL---V----------------------

S._moellendorfii_EAF1 GAQRPTT-NGSSNPSVTASATAAPPSVVTSSSSPRHKHSVLKLSILLRMA

B._distachyon_EAF1 --GLPC-MNQQKHE--------A---------------------------

A._thaliana_EAF1 TKGMPQASNDLKNI----KAVGS--TAV----------------------

A._thaliana_PIE1 --------------------------R-----------------------

B._distachyon_PIE1 --------------------------LL----------------------

S._moellendorfii_PIE1 -------------------AAGS---TA----------------------

P._patens_PIE1 --------------------------------------------------

H._sapiens_p400 ------------------TAAGQQVQMI----------------------

S._cerevisiae_Eaf1p --------------------------------------------------

S._pombe_Vid21 --------------------------------------------------

P._patens_EAF1 --------------------------------------------------

S._moellendorfii_EAF1 PPNFFYSGSGSTKLEEIHGSRTHADRMECNKDDAVKAVDLAEKKFMLHDL

B._distachyon_EAF1 --------------------------------------------------

A._thaliana_EAF1 --------------------------------------------------

A._thaliana_PIE1 --------------------------------------------------

B._distachyon_PIE1 --------------------------------------------------

S._moellendorfii_PIE1 --------------------------------------------------

P._patens_PIE1 --------------------------------------------------

H._sapiens_p400 --------------------------------------------------

S._cerevisiae_Eaf1p --------------------------------------------------

S._pombe_Vid21 --------------------------------------------------

P._patens_EAF1 --------------------------------------------------

S._moellendorfii_EAF1 AAARDFCVKALQLDPGLERGKQMLAVVEVHAAAAVRHHSLIILPSDLFGI

B._distachyon_EAF1 --------------------------------------------------

A._thaliana_EAF1 --------------------------------------------------

A._thaliana_PIE1 --------------------------------------------------

B._distachyon_PIE1 --------------------------------------------------

S._moellendorfii_PIE1 --------------------------------------------------

P._patens_PIE1 --------------------------------------------------

H._sapiens_p400 --------------------------------------------------

S._cerevisiae_Eaf1p --------------------------------------------------

S._pombe_Vid21 --------PRLQPYAVSSSL------------------------------

P._patens_EAF1 --------PDTR--------------------------------------

S._moellendorfii_EAF1 GDHDWYAILRVDPRADDASIRTQYRKMARLLHPDKTRMNGAEEAIKLVN-

B._distachyon_EAF1 ------------SANDVTSVTSTSKLIC----------SP-K--DNLVG-

A._thaliana_EAF1 --------PALEPPSCVASVQSTASKVV----------NNSN--TDSAGN

A._thaliana_PIE1 --------------------------------------------------

B._distachyon_PIE1 -----------------AS--------------------------A----

S._moellendorfii_PIE1 -----------------AA--------------------------AAAG-

P._patens_PIE1 --------------------------------------------------

H._sapiens_p400 --------PAVT-----ATAQV----------------------------

S._cerevisiae_Eaf1p ------------------QSSLSNISNINSAPRIKSPTPQ----------

S._pombe_Vid21 --------------------------------------------------

P._patens_EAF1 ---------------------------------DSAPGSSHSASSPMLID

S._moellendorfii_EAF1 EAKTVLSDKNKKMIYDSIRSSLPSTSNDVSAPPPRTPPPPQPPPYGTPTF

B._distachyon_EAF1 NGAS-LPSSSQELLQRKISGGLPMHGQDI---------------------

A._thaliana_EAF1 DPVS-TPN--QGLAQKH--------GIK----------------------

A._thaliana_PIE1 --------------------------------------------------

B._distachyon_PIE1 ---------AEF-----------HI--------A----------------

S._moellendorfii_PIE1 -GAN-YPFAQYW-----------RP--------DSTV-------------

P._patens_PIE1 --------------------------------------------------

H._sapiens_p400 --------VQQKLIQQQVVTTASAP--------LQTPGAPNPAQVPASSD

S._cerevisiae_Eaf1p --------------------------------------------------

S._pombe_Vid21 --------------------------------------------------

P._patens_EAF1 ---------------------------------------------NGQAR

S._moellendorfii_EAF1 VAQCPFCMAQWWYYKTFENYVLLCACCLRNFIVVNFHHLAWGYPGSDSWY

B._distachyon_EAF1 ---------------------------------------------GGQWH

A._thaliana_EAF1 ---------------------------------------------GVTQR

A._thaliana_PIE1 --------------------------------------------------

B._distachyon_PIE1 --------------------------------------------------

S._moellendorfii_PIE1 --------------------------------------------------

P._patens_PIE1 --------------------------------------------------

H._sapiens_p400 ---------------------------------------------S----

S._cerevisiae_Eaf1p --------------------------------------------------

S._pombe_Vid21 -----------------------KLTP-----------------------

P._patens_EAF1 QSGGVI-L------------------------------------------

S._moellendorfii_EAF1 QRQEEEATMKRREMEWAETMRMEREREEERKREARIKEAEDSRAEFLRMK

B._distachyon_EAF1 QQQSMQ-----------------HLQP-----------------------

A._thaliana_EAF1 QQQS---------------------LP-----------------------

A._thaliana_PIE1 --------------------------------------------------

B._distachyon_PIE1 --QSLS-----------------DFGI-----------------------

S._moellendorfii_PIE1 --SSSR-----------------LAQA-----------------------

P._patens_PIE1 --------------------------------------------------

H._sapiens_p400 -------P------------------------------------------

S._cerevisiae_Eaf1p --------------------------------------------------

S._pombe_Vid21 ----------------------------------EQ-IHQLQQRK-----

P._patens_EAF1 -----------------------------------SSPKTSVPRKP----

S._moellendorfii_EAF1 EREEERRREARIRQEKEDERRREARIKEERRREARINQDQEEERKR----

B._distachyon_EAF1 ----------------------------------PHHQQTQHQQRP----

A._thaliana_EAF1 ----------------------------------SEEKRPKLPEKP----

A._thaliana_PIE1 --------------------------------------EEEEEQEVDEKA

B._distachyon_PIE1 ----------------------------------NDSEFTCFHDL----P

S._moellendorfii_PIE1 ----------------------------------PASEEASYHRKP----

P._patens_PIE1 -------------------------------------------RKV----

H._sapiens_p400 -----------------------------------SQQPKLQMRVP----

S._cerevisiae_Eaf1p --------------------------------------------------

S._pombe_Vid21 ------------------------------------------------Q-

P._patens_EAF1 AVSAATA----------------------------ASDGISSGSTVS--S

S._moellendorfii_EAF1 EARIKHD------------QEERRREARIKERETRKQSEMDQRSKQGGAK

B._distachyon_EAF1 VVQ---------------------------------------G----SL-

A._thaliana_EAF1 TVQNQKH--------LASEEQPHL-----EEAQ--------EL----SS-

A._thaliana_PIE1 NSAEIEMISCSQWYDPFF--TSGL-----DD----------------CS-

B._distachyon_PIE1 LETDTEFAPCQ--YELAS--LPGI-----EE----------------LD-

S._moellendorfii_PIE1 -----------------------------------------------AA-

P._patens_PIE1 -----------------------------------------------AA-

H._sapiens_p400 AVRL----------------------------------------------

S._cerevisiae_Eaf1p ----------------------------------EILQRFQKQ

S._pombe_Vid21 ----------------------------------TVPTTERTQ

P._patens_EAF1 TTSGPG----------PPN---------------TEATPASSF

S._moellendorfii_EAF1 STNGNAGLVAVIVVLLVLGKGFILPAALLLALILVAFILSIRL

B._distachyon_EAF1 ----------------------------------YAPSNSGSG

A._thaliana_EAF1 ----------------------------------SKPPDTKVE

A._thaliana_PIE1 ----------------------------------LASDISEIE

B._distachyon_PIE1 ----------------------------------PLSDFTDIG

S._moellendorfii_PIE1 ----------------------------------AAKNKGVSE

P._patens_PIE1 ----------------------------------KHGSEKLLT

H._sapiens_p400 ----------------------------------KTPTKPPCQ
