# Supplementary material for: AtEAF1 is a potential platform protein for Arabidopsis NuA4 acetyltransferase complex
Source: BMC Plant Biol. 2015 Mar 5;15:75. doi: 10.1186/s12870-015-0461-1 (PMC4358907; doi:10.1186/s12870-015-0461-1)
Supplement: Additional file 4: — Gene structures of AtYAF9A , AtYAF9B and AtEAF1B with positions of T-DNA insertions in the mutant lines marked. Details of the amiRNA design and phenotypes of the silenced lines. Relative expression levels of the AtEAF1A and AtEAF1B genes. [file 12870_2015_461_MOESM4_ESM.pptx]

## Slide 1
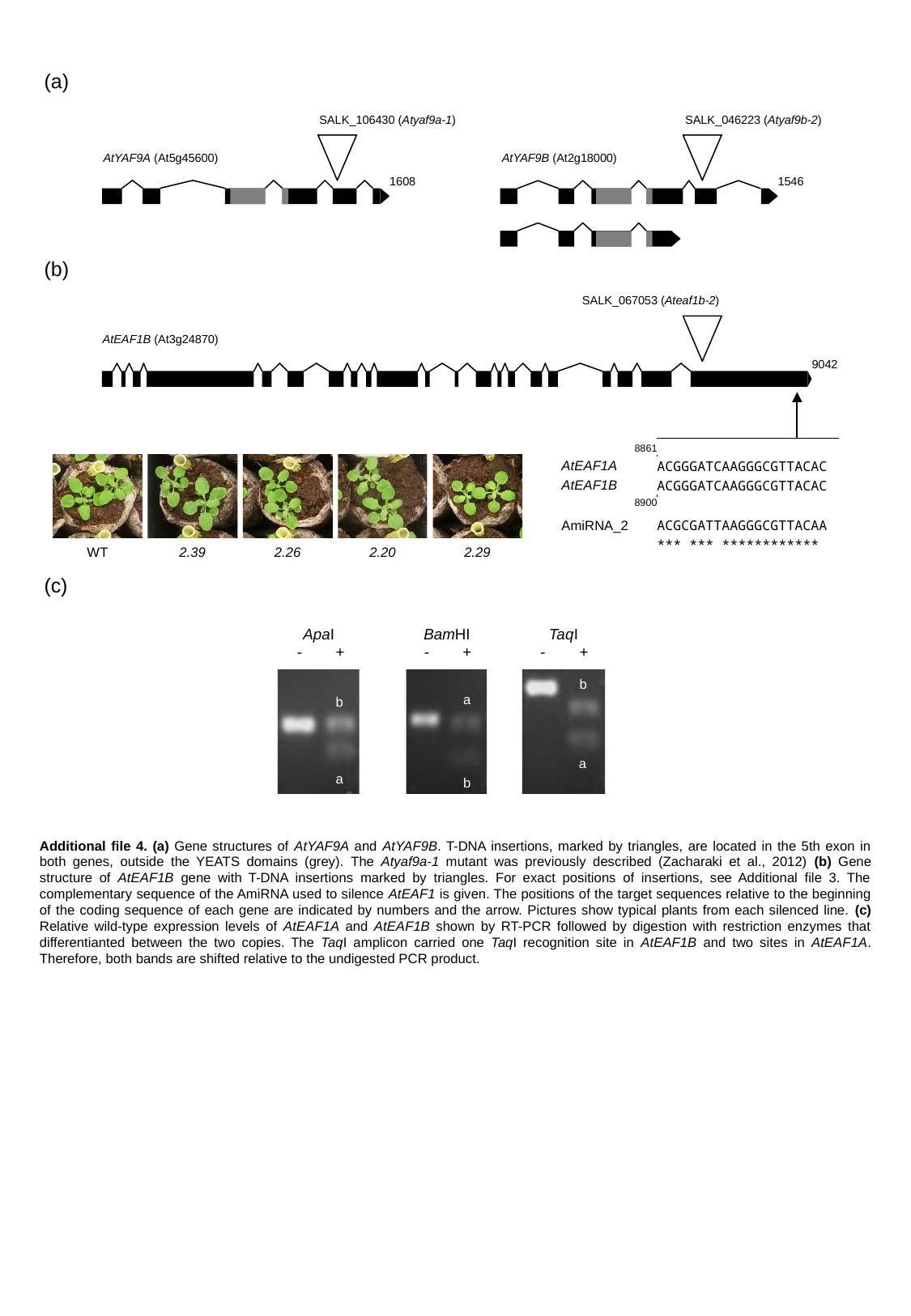

(a)
SALK_106430 (Atyaf9a-1)
SALK_046223 (Atyaf9b-2)
1608
AtYAF9A (At5g45600)
AtYAF9B (At2g18000)
1546
(b)
SALK_067053 (Ateaf1b-2)
AtEAF1B (At3g24870)
9042
| 8861 | |
| --- | --- |
| | |
| AtEAF1A | ACGGGATCAAGGGCGTTACAC |
| AtEAF1B | ACGGGATCAAGGGCGTTACAC |
| | |
| 8900 | |
| AmiRNA\_2 | ACGCGATTAAGGGCGTTACAA |
| | \*\*\* \*\*\* \*\*\*\*\*\*\*\*\*\*\*\* |
2.39
2.20
WT
2.26
2.29
(c)
| ApaI | | | BamHI | | | TaqI | |
| --- | --- | --- | --- | --- | --- | --- | --- |
| - | + | | - | + | | - | + |
b
a
a
b
b
a
Additional file 4. (a) Gene structures of AtYAF9A and AtYAF9B. T-DNA insertions, marked by triangles, are located in the 5th exon in both genes, outside the YEATS domains (grey). The Atyaf9a-1 mutant was previously described (Zacharaki et al., 2012) (b) Gene structure of AtEAF1B gene with T-DNA insertions marked by triangles. For exact positions of insertions, see Additional file 3. The complementary sequence of the AmiRNA used to silence AtEAF1 is given. The positions of the target sequences relative to the beginning of the coding sequence of each gene are indicated by numbers and the arrow. Pictures show typical plants from each silenced line. (c) Relative wild-type expression levels of AtEAF1A and AtEAF1B shown by RT-PCR followed by digestion with restriction enzymes that differentianted between the two copies. The TaqI amplicon carried one TaqI recognition site in AtEAF1B and two sites in AtEAF1A. Therefore, both bands are shifted relative to the undigested PCR product.
