# Supplementary material for: AtEAF1 is a potential platform protein for Arabidopsis NuA4 acetyltransferase complex
Source: BMC Plant Biol. 2015 Mar 5;15:75. doi: 10.1186/s12870-015-0461-1 (PMC4358907; doi:10.1186/s12870-015-0461-1)
Supplement: Additional file 5: — Uncropped scans of the Western blot films. [file 12870_2015_461_MOESM5_ESM.pptx]

## Slide 1
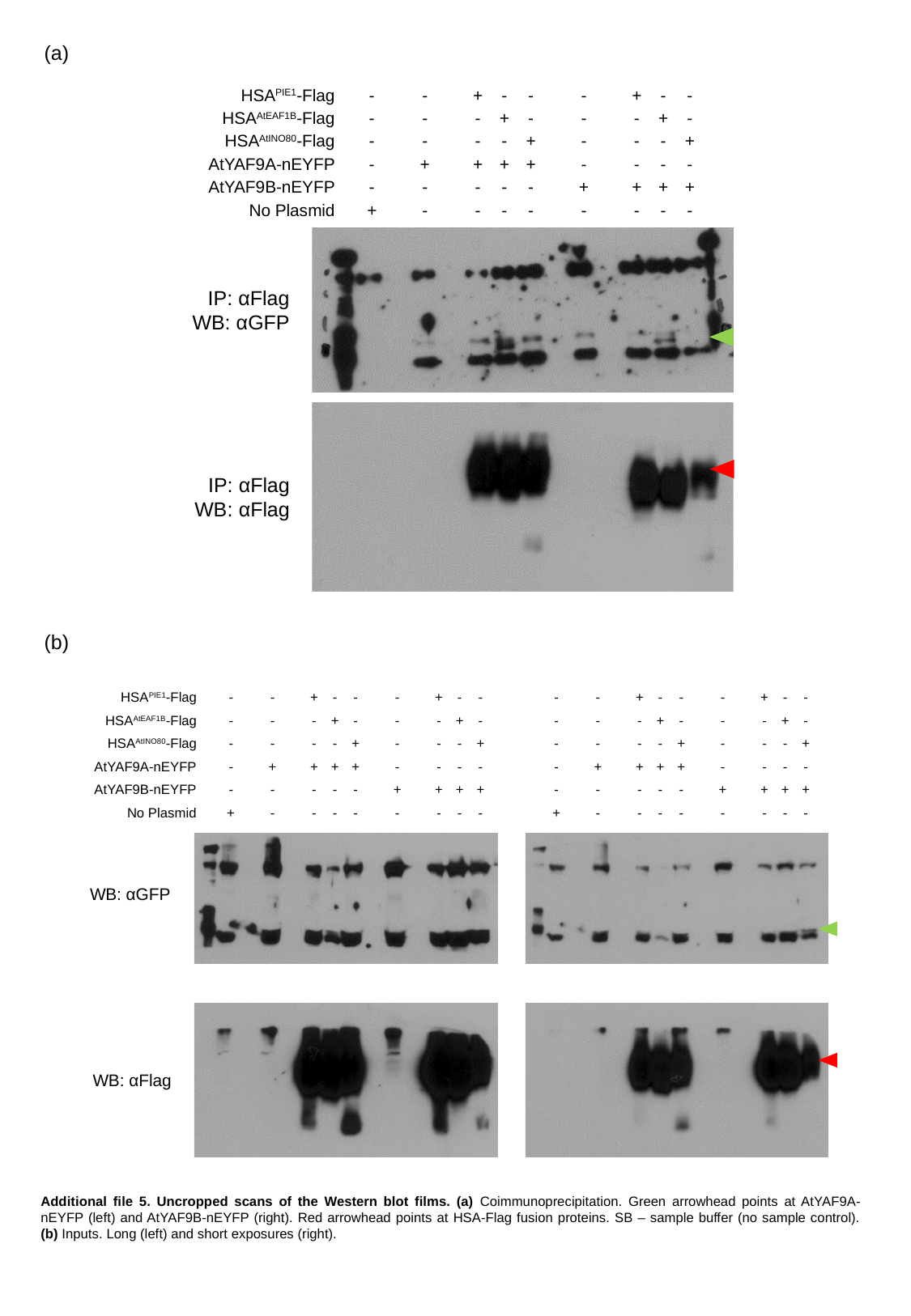

(a)
| HSAPIE1-Flag | - | | - | | + | - | - | | - | | + | - | - |
| --- | --- | --- | --- | --- | --- | --- | --- | --- | --- | --- | --- | --- | --- |
| HSAAtEAF1B-Flag | - | | - | | - | + | - | | - | | - | + | - |
| HSAAtINO80-Flag | - | | - | | - | - | + | | - | | - | - | + |
| AtYAF9A-nEYFP | - | | + | | + | + | + | | - | | - | - | - |
| AtYAF9B-nEYFP | - | | - | | - | - | - | | + | | + | + | + |
| No Plasmid | + | | - | | - | - | - | | - | | - | - | - |
IP: αFlag
WB: αGFP
IP: αFlag
WB: αFlag
(b)
| HSAPIE1-Flag | - | | - | | + | - | - | | - | | + | - | - |
| --- | --- | --- | --- | --- | --- | --- | --- | --- | --- | --- | --- | --- | --- |
| HSAAtEAF1B-Flag | - | | - | | - | + | - | | - | | - | + | - |
| HSAAtINO80-Flag | - | | - | | - | - | + | | - | | - | - | + |
| AtYAF9A-nEYFP | - | | + | | + | + | + | | - | | - | - | - |
| AtYAF9B-nEYFP | - | | - | | - | - | - | | + | | + | + | + |
| No Plasmid | + | | - | | - | - | - | | - | | - | - | - |
| - | | - | | + | - | - | | - | | + | - | - |
| --- | --- | --- | --- | --- | --- | --- | --- | --- | --- | --- | --- | --- |
| - | | - | | - | + | - | | - | | - | + | - |
| - | | - | | - | - | + | | - | | - | - | + |
| - | | + | | + | + | + | | - | | - | - | - |
| - | | - | | - | - | - | | + | | + | + | + |
| + | | - | | - | - | - | | - | | - | - | - |
WB: αGFP
WB: αFlag
Additional file 5. Uncropped scans of the Western blot films. (a) Coimmunoprecipitation. Green arrowhead points at AtYAF9A-nEYFP (left) and AtYAF9B-nEYFP (right). Red arrowhead points at HSA-Flag fusion proteins. SB – sample buffer (no sample control). (b) Inputs. Long (left) and short exposures (right).
