# Supplementary material for: AtEAF1 is a potential platform protein for Arabidopsis NuA4 acetyltransferase complex
Source: BMC Plant Biol. 2015 Mar 5;15:75. doi: 10.1186/s12870-015-0461-1 (PMC4358907; doi:10.1186/s12870-015-0461-1)
Supplement: Additional file 6: — BiFC screening for protein-protein interactions between AtARP4, AtSWC4, AtYAF9A and AtYAF9B. [file 12870_2015_461_MOESM6_ESM.pptx]

## Slide 1
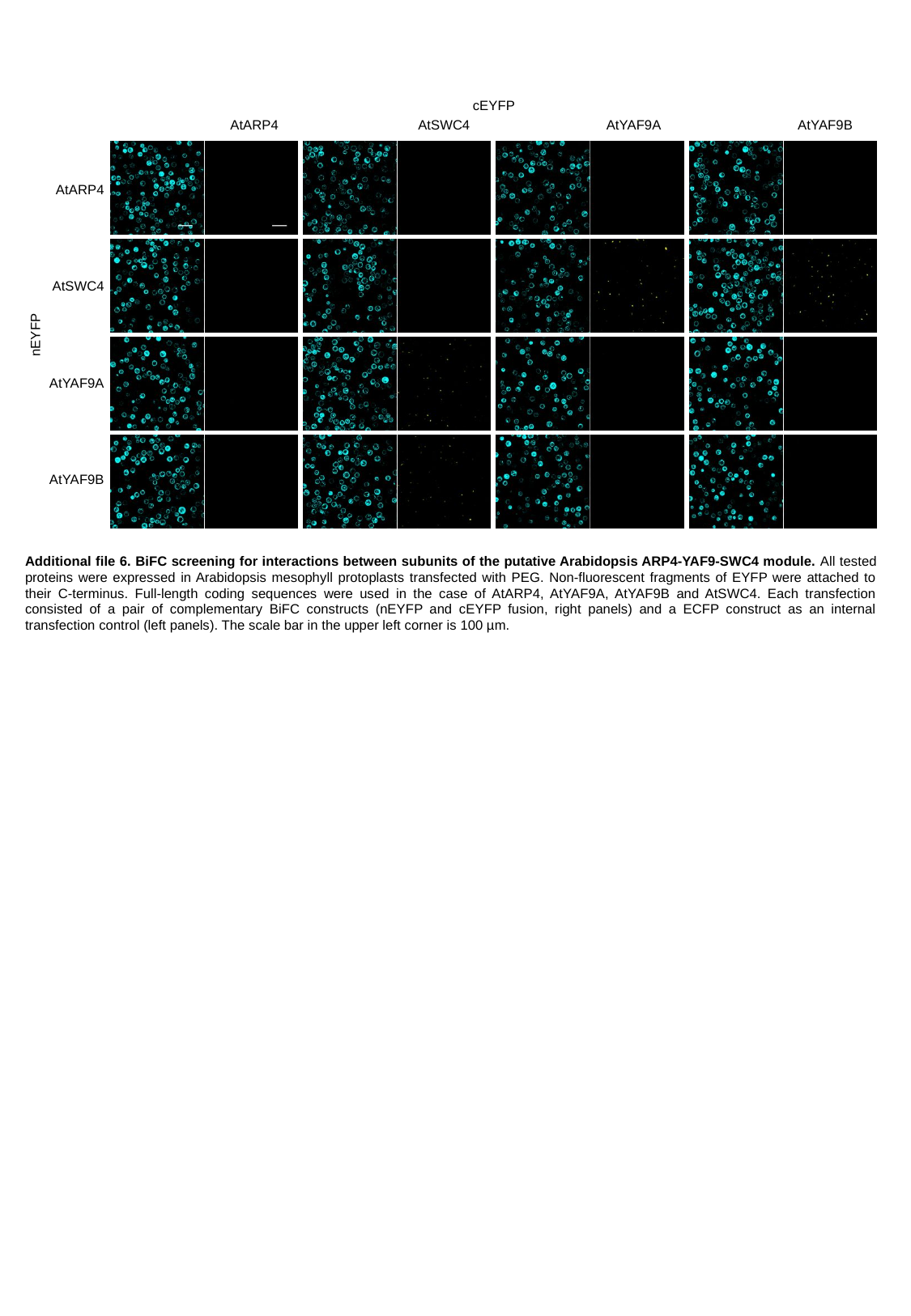

| cEYFP | | | |
| --- | --- | --- | --- |
| AtARP4 | AtSWC4 | AtYAF9A | AtYAF9B |
| nEYFP | AtARP4 |
| --- | --- |
| | AtSWC4 |
| | AtYAF9A |
| | AtYAF9B |
Additional file 6. BiFC screening for interactions between subunits of the putative Arabidopsis ARP4-YAF9-SWC4 module. All tested proteins were expressed in Arabidopsis mesophyll protoplasts transfected with PEG. Non-fluorescent fragments of EYFP were attached to their C-terminus. Full-length coding sequences were used in the case of AtARP4, AtYAF9A, AtYAF9B and AtSWC4. Each transfection consisted of a pair of complementary BiFC constructs (nEYFP and cEYFP fusion, right panels) and a ECFP construct as an internal transfection control (left panels). The scale bar in the upper left corner is 100 µm.
