# Supplementary material for: AtEAF1 is a potential platform protein for Arabidopsis NuA4 acetyltransferase complex
Source: BMC Plant Biol. 2015 Mar 5;15:75. doi: 10.1186/s12870-015-0461-1 (PMC4358907; doi:10.1186/s12870-015-0461-1)
Supplement: Additional file 7: — Western Blot analyses of bulk H4K5 acetylation levels in mutants and WT plants treated or untreated with TSA. [file 12870_2015_461_MOESM7_ESM.pptx]

## Slide 1
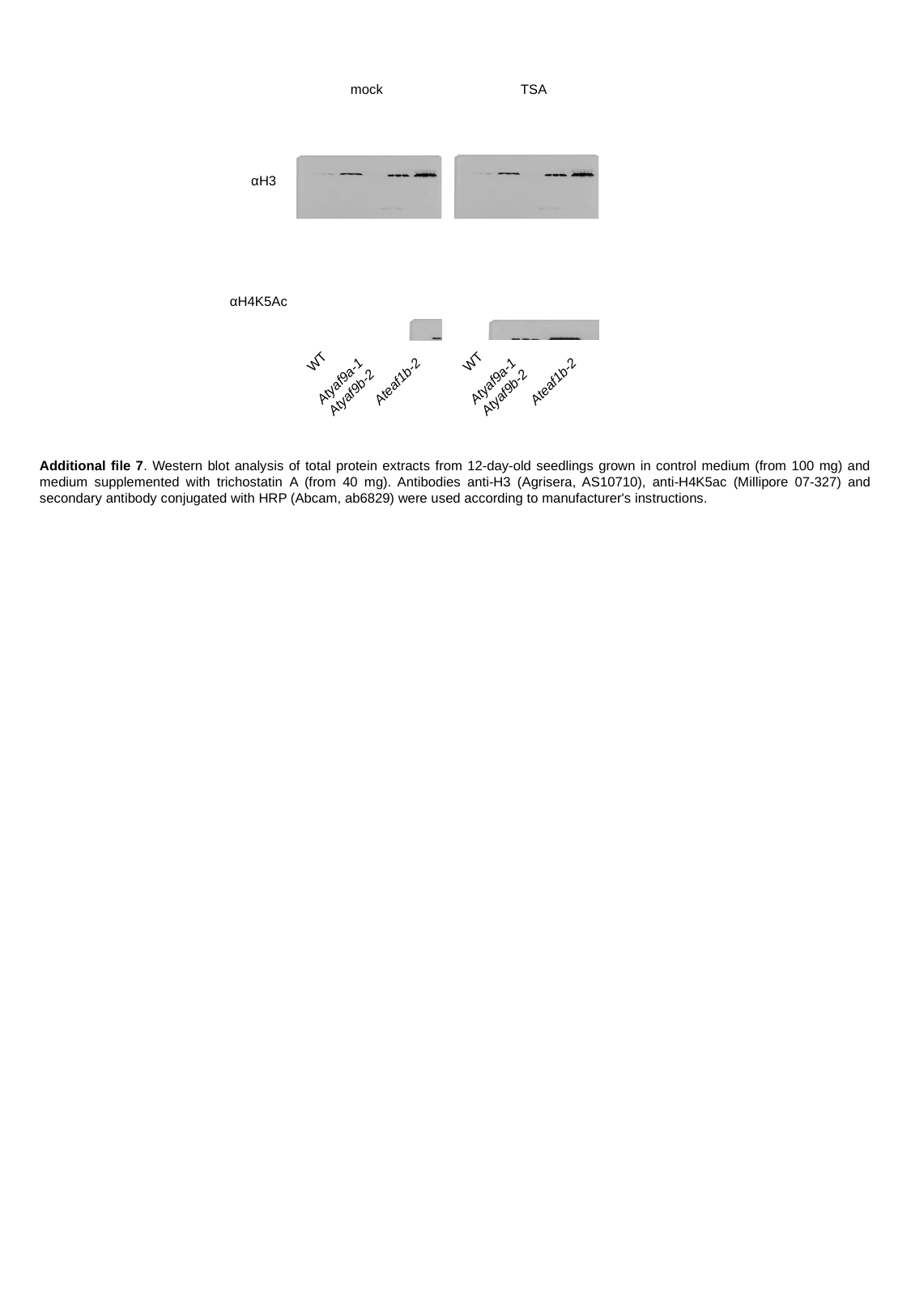

mock
TSA
αH3
αH4K5Ac
WT
Atyaf9a-1
Atyaf9b-2
Ateaf1b-2
WT
Atyaf9a-1
Atyaf9b-2
Ateaf1b-2
Additional file 7. Western blot analysis of total protein extracts from 12-day-old seedlings grown in control medium (from 100 mg) and medium supplemented with trichostatin A (from 40 mg). Antibodies anti-H3 (Agrisera, AS10710), anti-H4K5ac (Millipore 07-327) and secondary antibody conjugated with HRP (Abcam, ab6829) were used according to manufacturer's instructions.
